# W246G Mutant ELOVL4 Impairs Synaptic Plasticity in Parallel and Climbing Fibers and Causes Motor Defects in a Rat Model of SCA34

**DOI:** 10.1007/s12035-021-02439-1

**Published:** 2021-07-05

**Authors:** Raghavendra Y. Nagaraja, David M. Sherry, Jennifer L. Fessler, Megan A. Stiles, Feng Li, Karanpreet Multani, Albert Orock, Mohiuddin Ahmad, Richard S. Brush, Robert E. Anderson, Martin-Paul Agbaga, Ferenc Deák

**Affiliations:** 1 Biochemistry & Molecular Biology, University of Oklahoma Health Sciences Center, 608 Stanton L. Young Blvd, DMEI 428PP, Oklahoma City, OK 73104 USA; 2 Neuroscience Program, University of Oklahoma Health Sciences Center, 608 Stanton L. Young Blvd, DMEI 428PP, Oklahoma City, OK 73104 USA; 3 Cell Biology, University of Oklahoma Health Sciences Center, 608 Stanton L. Young Blvd, DMEI 428PP, Oklahoma City, OK 73104 USA; 4 Pharmaceutical Sciences, University of Oklahoma Health Sciences Center, 608 Stanton L. Young Blvd, DMEI 428PP, Oklahoma City, OK 73104 USA; 5 Ophthalmology, University of Oklahoma Health Sciences Center, 608 Stanton L. Young Blvd, DMEI 428PP, Oklahoma City, OK 73104 USA; 6 Dean McGee Eye Institute, University of Oklahoma Health Sciences Center, 608 Stanton L. Young Blvd, DMEI 428PP, Oklahoma City, OK 73104 USA; 7 Reynolds Center on Aging, University of Oklahoma Health Sciences Center, 608 Stanton L. Young Blvd, DMEI 428PP, Oklahoma City, OK 73104 USA; 8 Harold Hamm Diabetes Center, University of Oklahoma Health Sciences Center, 608 Stanton L. Young Blvd, DMEI 428PP, Oklahoma City, OK 73104 USA; 9 Dept. of Neuroscience & Regenerative Medicine, Medical College of Georgia, 1120 15th Str, CA4010, Augusta, GA 30912 USA

**Keywords:** Spinocerebellar ataxia-34 (SCA34), Elongation of Very Long Chain Fatty Acids-4 (ELOVL4), Cerebellum, Electrophysiology, Very Long Chain Fatty Acids (VLC-FA)

## Abstract

Spinocerebellar ataxia (SCA) is a neurodegenerative disorder characterized by ataxia and cerebellar atrophy. A number of different mutations gives rise to different types of SCA with characteristic ages of onset, symptomatology, and rates of progression. SCA type 34 (SCA34) is caused by mutations in *ELOVL4* (ELOngation of Very Long-chain fatty acids 4), a fatty acid elongase essential for biosynthesis of Very Long Chain Saturated and Polyunsaturated Fatty Acids (VLC-SFA and VLC-PUFA, resp., ≥28 carbons), which have important functions in the brain, skin, retina, Meibomian glands, testes, and sperm. We generated a rat model of SCA34 by knock-in of the SCA34-causing 736T>G (p.W246G) *ELOVL4* mutation. Rats carrying the mutation developed impaired motor deficits by 2 months of age. To understand the mechanism of these motor deficits, we performed electrophysiological studies using cerebellar slices from rats homozygous for W246G mutant ELOVL4 and found marked reduction of long-term potentiation at parallel fiber synapses and long-term depression at climbing fiber synapses onto Purkinje cells. Neuroanatomical analysis of the cerebellum showed normal cytoarchitectural organization with no evidence of degeneration out to 6 months of age. These results point to ELOVL4 as essential for motor function and cerebellar synaptic plasticity. The results further suggest that ataxia in SCA34 patients may arise from a primary impairment of synaptic plasticity and cerebellar network desynchronization before onset of neurodegeneration and progression of the disease at a later age.

**Supplementary Information:**

The online version contains supplementary material available at 10.1007/s12035-021-02439-1.

## Introduction

Lipids are fundamental biological molecules that play many important roles in the central nervous system. Errors in lipid metabolism lead to a variety of neurological diseases. Very Long Chain Saturated and Polyunsaturated Fatty Acids (VLC-SFA and VLC-PUFA, resp., ≥28 carbons) are essential for life, but their functions remain largely obscure. The enzyme ELOngation of Very Long chain fatty acids-4 (ELOVL4) catalyzes the first and rate-limiting step in VLC-SFA and VLC-PUFA biosynthesis and is the only fatty acid elongase that serves this function [1–3]. ELOVL4 is expressed in only a small number of organs, including the brain, retina, skin, Meibomian glands, and testes [4–6], each of which shows a tissue-specific profile of VLC-SFA and VLC-PUFA.

ELOVL4 and its VLC-SFA and VLC-PUFA products are of critical importance to the CNS [7]. Three distinct groups of mutations in the *ELOVL4* gene give rise to three distinct CNS diseases. Heterozygous inheritance of several different *ELOVL4* mutations in exon 6 that cause early truncation of the ELOVL4 protein and loss of the ER-retention signal in the C-terminus leads to autosomal dominant Stargardt-like macular dystrophy (STGD3), an aggressive juvenile-onset macular degeneration [8–11]. Patients with STGD3 show no CNS or skin phenotypes. Heterozygous inheritance of several different point mutations in *ELOVL4* causes autosomal dominant spinocerebellar ataxia-34 (SCA34) with or without erythrokeratodermia variabilis (EKV, a skin condition) [12–17]. SCA34 patients typically show no retinal phenotype, although a recent report identified a point mutation (c.512T>C, p.I171T) in exon 4 of *ELOVL4* as a cause of SCA34 that also presents with retinitis pigmentosa in some of the affected family members [17]. Homozygous inheritance of other recessive *ELOVL4* mutations that result in truncation of the ELOVL4 protein causes a neuro-ichthyotic syndrome characterized by severe seizures, intellectual disability, spasticity, ichthyosis, and early death [18, 19]. No human patients with homozygous inheritance of STGD3 or SCA34 alleles have been reported.

Our lipidomic analyses of mouse, rat, and baboon brains found that the main VLC-fatty acid species in the brain are VLC-SFA (primarily 28:0 and 30:0) that are incorporated into complex sphingolipids [3, 20]. Furthermore, VLC-SFA were enriched specifically in synaptic vesicles prepared from baboon hippocampus and found to modulate presynaptic transmitter release kinetics in cultured mouse hippocampal neurons [3]. In contrast, we have not been able to identify VLC-PUFA in brains of 2-, 12-, and 24-month-old mice. However, VLC-PUFA have been reported in the early postnatal rat brain and the brains of young patients afflicted with Zellweger’s disease, a peroxisomal disorder that disrupts lipid metabolism [21, 22]. Based on these studies, VLC-PUFA appear to be present only at trace levels in the healthy, mature brain. In contrast, the main VLC-fatty acids in the retina are VLC-PUFA incorporated into phosphatidylcholine in the outer segments of rod and cone photoreceptors [23–26], although VLC-SFA incorporated into non-sialylated sphingolipids also have been found [20]. VLC-PUFA in an amide linkage to sphingolipids are present in testes [27–29]. VLC-SFA are also synthesized in the Meibomian glands and skin, where they are incorporated into ω-O-acylceramides and form the water barrier of the tear film and skin, respectively [5, 30–33]. Our recent studies of W246G ELOVL4 function in the retina and skin showed that the W246G mutation selectively impaired synthesis of VLC-SFA (28:0 decreased to around 60%, and 30:0 decreased to 10% of levels in skin of WT rats), but had no significant effect on VLC-PUFA synthesis [34].

SCAs arise from defects in a large number of different genes, which produce distinct forms of SCA with characteristic times of onset and rates of disease progression [35, 36]. It has been suggested from the published SCA studies that cerebellar dysfunction is caused by altered output from the cerebellar cortex. The cerebellar Purkinje cells (PCs) are the sole output neuron of the cerebellar cortex. Thus, understanding the effects of SCA-causing mutations on PC synaptic inputs and outputs is critical to understanding the underlying disease process. The excitatory inputs to PCs are synapses formed by the parallel fibers (PF) from granule cells and climbing fibers (CF) from inferior olive neurons. Changes in synaptic plasticity at PF and CF inputs to PCs are thought to be critical to motor function and learning. Our previous studies showed that ELOVL4 is prominently expressed in the granule cells, molecular layer interneurons, and Purkinje cells of the cerebellum as well as in the inferior olivary complex [37], which provides the CF input to the cerebellum. This is consistent with the clinical and pathological findings that *ELOVL4* mutations cause SCA34, although the effects of these mutations on the cytoarchitectural organization and synaptic plasticity of the cerebellum are unknown.

To better understand the functional role of ELOVL4 and its products in synaptic function and SCA34 pathology, we generated a rodent model of the human SCA34-causing *Elovl4* mutation reported by Ozaki et al. [16] using CRISPR-Cas9 gene editing to replace one copy of the rat *Elovl4* gene with the c.736T>G (p.W246G) mutation in the Long Evans rat genome [34]. We used a range of behavioral, biochemical, anatomical, and *ex vivo* electrophysiological studies to evaluate the effect of the W246G ELOVL4 mutation on motor function, cerebellar organization, and synaptic function. This is the first report of an animal model of how mutations in ELOVL4 contribute to age-related cerebellar degeneration and loss of motor coordination in SCA34 patients. Our results also underscore the essential role of ELOVL4 products in neuronal health and aging and may have broader implications for age-related loss of motor coordination independent of ELOVL4 mutations.

## Materials and Methods

### Animals

A CRISPR/Cas9 gene editing approach was used to generate F0 founder Long-Evans rats with heterozygous knock-in of the c.736T>G, p.W246G mutation in *ELOVL4* that causes human SCA34 [16]. These founders were bred to WT Long-Evans rats to establish a breeding colony. Details of the generation of the knock-in rat line are described in Agbaga et al. [34]. All experiments were performed using WT, HET, and MUT rats, with genotype confirmed by PCR. Rats were maintained in a pathogen-free barrier facility on a 12-h light:12-h dark daily light cycle (~25–40 lux at cage level) with food and water available at all times. All animal procedures were approved by the Institutional Animal Care and Use Committee of the University of Oklahoma Health Sciences Center and conformed to the National Institute of Health Guide for the Care and Use of Laboratory Animals, US Public Health Service guidelines, and the Association for Research in Vision and Ophthalmology Resolution on the Use of Animals in Research.

### Western Blotting

The lysate of rat cerebellum was prepared by homogenizing the tissue in 20 mM Tris HCl (pH 7.5), 150 mM NaCl, 1 mM EGTA, 1% Triton X-100, and EDTA-free Protease Inhibitor Cocktail (Sigma-Aldrich, St Louis, MO). Protein concentration was determined using a Bicinchonic Acid kit (Sigma-Aldrich, St. Louis, MO) after which 25 μg of total unboiled protein lysates were resolved on 4–20% polyacrylamide gels and transferred to nitrocellulose membrane. ELOVL4 was detected using previously characterized rabbit polyclonal ELOVL4 antibodies [1] that have been confirmed by other labs [38, 39] to recognize ≈32-kDa ELOVL4 protein from mouse, rat, bovine, and porcine ELOVL4-expressing tissues and by specifically block binding by pre-incubation of the antibody with the antigenic peptide [1]. Immunolabeling of nitrocellulose membranes with this and other primary antibodies (Table 1) was performed at room temperature for 2 h in Intercept TBS Blocking Buffer (LI-COR). Following incubation with fluorescent dye-coupled secondary antibodies (Table 1), the membranes were imaged sequentially in 700-nm and 800-nm channels on an Odyssey Fc Imaging System (LI-COR) using the Image Studio acquisition program (LI-COR). The intensity of the bands (summated signal in the selected area) was quantified using Image Studio Lite (LI-COR). The intensity value of the band of interest was normalized to the loading control in the same lane.

**Table 1:** Antibodies

Antibodies		
Elongation of very long-chain fatty Acids-4 (ELOVL4; rabbit polyclonal)	Dr. M-P Agbaga.	N/A
Calbindin (mouse monoclonal)	Sigma/Millipore	Cat# C9848, clone CB-955; RRID:AB_476894
Calbindin (chicken polyclonal)	Synaptic Systems	Cat# 214 006; RRID:AB_2619903
Glial Fibrillary Acidic Protein (GFAP; mouse monoclonal)	Sigma/Millipore	Cat# MAB360, clone GA-5; RRID:AB_2109815
Glutamic Acid Decarboxylase 65/67 (GAD65, GAD67; rabbit polyclonal)	Sigma/Millipore	Cat# G5163; RRID:AB_477019
GluA1 (mouse monoclonal)	NeuroMab	Cat# 75-327, clone N355/1; RRID: AB_2315840
GluA2 (mouse monoclonal)	NeuroMab	Cat# 75-002, clone L21/32; RRID: AB_2877267
Glyceraldehyde-3-Phosphate Dehydrogenase (GAPDH; rabbit polyclonal)	ProteinTech	Cat# 10494-1-AP; RRID: AB_2263076
Inositol Phosphate 3 Receptor, Type 1 (IP3R, mouse monoclonal)	Antibodies Incorporated	Cat# 75-035, clone L24/18; RRID: AB_10000362
mGluR1a (rabbit polyclonal)	Sigma/Millipore	Cat# G9665 RRID:AB_259995
NeuN (Mouse monoclonal)	Sigma/Millipore	Cat# MAB377, clone A60; RRID:AB_2298772
Protein Kinase C Gamma (PKG, mouse monoclonal)	Invitrogen (Thermo-Fisher)	Cat# 13-3800; Clone PKC 66; RRID:AB_2533015
PSD-95 (mouse monoclonal)	NeuroMab	Cat# 75-028, clone K28/43; RRID: AB_2292909
Synaptotagmin 2 (mouse monoclonal)	Zebrafish International Resource Center	Cat# znp-1; clone znp-1; RRID:AB_10013783
Vesicular Glutamate Transporter 1 (VGluT1; mouse monoclonal)	Neuromab	Cat# 75-066, clone N28/9; RRID:AB_2187693
Vesicular Glutamate Transporter 2 (VGluT2; guinea pig polyclonal)	Sigma/Millipore	Cat# AB2251-I RRID:AB_2665454
Goat anti-rabbit IgG AlexaFluor488	Thermo/Molecular Probes	Cat# A-11008; RRID:AB_143165
Goat anti-rabbit IgG AlexaFluor568	Thermo/Molecular Probes	Cat# A11036; RRID:AB_143011
Goat anti-Mouse IgG AlexaFluor488	Thermo/Molecular Probes	Cat# A11001; RRID:AB_2534069
Goat anti-Mouse IgG AlexaFluor568	Thermo/Molecular Probes	Cat# A11004; RRID:AB_2534072
Goat anti-Chicken IgY AlexaFluor488	Thermo/Molecular Probes	Cat# A11039; RRID:AB_142294
Goat anti-Mouse IgG IR800	LI-COR	Cat# 926-32210; RRID:AB_621842
Goat anti-Rabbit IgG IR800	LI-COR	Cat# 926-32211; RRID:AB_621843
Goat anti-Mouse IgG AlexaFluor680	Jackson ImmunoResearch	Cat# 115-625-146; RRID:AB_2338935
Goat anti-Rabbit IgG AlexaFluor680	Jackson ImmunoResearch	Cat# 111-625-144; RRID:AB_2338085

### Assessment of Locomotor Function

Testing of locomotor function was performed using the rotarod test on WT, HET, and MUT rats at 2 to 6 months of age. All motor testing was performed under constant illumination conditions by the same experimenter, who was masked to the animals’ genotype. To assess motor learning, coordination, and balance, rats were placed on a rotarod apparatus with a roller diameter of 3.75 inches and five separated, equally sized individual animal compartments (IITC Life Science; Woodland Hills, CA), and presented with an accelerating rotation speed of 25–45 rpm for 5 min. Each animal was tested three times in each session and the mean total distance traveled and time to fall/completion were determined (data not shown) for each animal as measures of motor function [40–42]. Rats were trained on the accelerating assay over the course of several days to master the task prior to testing.

### Histology, Immunolabeling, and Imaging

For histology and immunolabeling, rats were anesthetized by intraperitoneal injection of ketamine (100 mg/kg body weight) and xylazine (5 mg/kg body weight) and perfused through the left ventricle with 0.1 M phosphate buffered saline (PBS, pH 7.2), followed by 4% paraformaldehyde in PBS for 20 min. Following perfusion, the brain was removed from the head, hemisected along the sagittal plane, and immersed in 4% paraformaldehyde in 0.1 M PBS or 0.1 M cacodylate buffer at 4 °C for several days. Following fixation, brains were rinsed extensively in PBS, cryoprotected in 30% sucrose in PBS, and then embedded in Optimal Cutting Temperature medium (OCT; Sakura Tissue Tek; VWR, West Chester, PA) and frozen. Sagittally oriented frozen sections (10- to 15-μm thickness) were prepared on a cryostat, collected onto Superfrost Plus slides (Fisher Scientific, Pittsburgh, PA), and stored at −20 to −30 °C until use.

Sections for morphometric analyses were stained using 1% toluidine blue and imaged using a Leica M205-MFC imaging system. Morphometric measurements were obtained from lobules III, V, and X using sagittal sections that ranged from 0 to 1320 μm from the midline. Images were imported into FIJI (NIH Image) software, image scale was calibrated, and brightness and contrast were adjusted to highlight cortical layering and Purkinje cell bodies. The linear extent of each cortical layer was measured along a perpendicular line extending from the cortical surface to the inner margin of the granule cell layer. Analysis of the linear density of Purkinje cells was performed in the same tissue sections by counting the number of Purkinje cell bodies in well-oriented, linear regions of the Purkinje cell layer, avoiding curved areas of the proximal and distal regions of the lobules.

Immunolabeling was performed as described previously [3, 37]. Antibody information is provided in Table 1. Sections were rehydrated in Hank’s buffered salt solution (HBSS) and non-specific labeling was blocked using a solution containing 2–10% normal goat serum, 5% bovine serum albumin, and 1% fish gelatin in 0.1–0.5% Triton X-100 in HBSS (“blocker”). In some experiments, antigen retrieval in 10 mM citrate buffer (pH 6.0; heated to 95 °C) was performed for 30–60 min prior to blocking. Blocker was removed and one or more primary antibodies were applied overnight at room temperature. Sections were rinsed in HBSS and appropriate secondary antibodies were applied for 60–75 min at room temperature. Sections were rinsed and mounted using Prolong Gold + DAPI or Prolong Diamond + DAPI to retard photobleaching. Specificity of the labeling method was confirmed by omitting primary antibodies and by substituting normal rabbit serum for primary antibody.

Fluorescence microscopy was performed using an Olympus IX70 inverted fluorescence microscope fitted with a QiCAM CCD camera controlled via QCapture software (QImaging), or a Leica M205-MFC microscope controlled via LasX software. Image scale was calibrated and figures were prepared using Photoshop software. If needed, adjustments to brightness and contrast were applied equally to all pixels in the image to highlight specific labeling.

### Electrophysiology

#### Slice Preparation

Both male and female rats at 3–4 months of age were used. The rats were decapitated using a guillotine and the cerebellum was quickly removed and cooled with ice-cold artificial CSF (ACSF) containing the following: 126 mM NaCl, 2.5 mM KCl, 1.25 mM NaH_2_PO_4_, 26 mM NaHCO_3_, 1.0 mM CaCl_2_, 1.0 mM MgCl_2_, 2.0 mM pyruvic acid, 0.4 mM ascorbic acid, and 10 mM D-glucose (final pH 7.4, oxygenated with 95% O_2_/5% CO_2_). The cerebellum was trimmed and the tissue block containing the cerebellar vermis was glued to the stage of a vibrating tissue slicer. Sagittal slices of about 350 μm were collected in oxygenated cold sucrose slicing solution containing 240 mM sucrose, 25 mM NaCl, 2.5 mM KCl, 1.25 mM NaH_2_PO_4_, 26 mM NaHCO_3_, 0.4 mM ascorbic acid, 10 mM D-glucose, 10 mM MgCl_2_, and 2 mM pyruvic acid (final pH 7.4) using a HM650V vibrating microtome (Thermo Scientific, Burlington, ON). The slices were transferred to a holding chamber containing oxygenated ACSF at room temperature and allowed to recover for at least 1 h.

#### Multi-electrode Array Recordings

For recording network activity and extracellular field potentials, the slice was transferred to and positioned on a multi-electrode array system (Alpha MED Scientific, Inc., Osaka, Japan) with a hexagonal electrode arrangement of 61 electrodes and electrode spacing of 70 μm (MED-P2H07A). Under the dissecting microscope, three layers of the cerebellar cortex, including the molecular layer, Purkinje cell layer, and granule cell layer, and the white matter could readily be identified in each folium. To secure contact between the slice and electrodes and to improve mechanical stability, a piece of nylon mesh and a slice anchor harp were placed on top of the slice. The slice was maneuvered to ensure that the hexagonal array was positioned within a folium and that the electrodes covered all three layers of the cortex. The chamber was perfused with oxygenated ACSF at a rate of 2 mL/min at 32 °C. After the slice had settled in the recording chamber, before applying any stimulation, six hundred (600) traces of network activity were recorded, each for a 1-s duration, under physiological conditions with continuous ACSF perfusion [3]. Network activity data were analyzed as previously described [3]. Briefly, raw MED64 Mobius workflow files were opened and spikes were extracted in Mobius (© WitWerx, Inc.). Positive and negative spike threshold was set to + 0.021 and −0.021 mV, respectively. Spike traces were extracted along with 1 ms of baseline before and after the spike event, without down sampling. Raw data were filtered using a Bessel high-pass (2-pole) with a cutoff frequency of 1000 Hz and a DC filter with a typical spike length set to 1 ms. The resulting file containing all extracted spikes within that slice recording was then processed in Microsoft Excel using a visual basic macro that was coded and validated by us to extract each of the final parameters for statistical comparison.

All evoked responses were obtained by stimulating one electrode in the molecular layer and one electrode in the granular cell layer to evoke responses in the molecular layer. Input/output curves (I/O curves) were generated by applying increasing stimulus currents to the pathway from 5 to 100 μA and recording the responses. The threshold stimulus for generating fEPSPs was determined as 30–40% of the stimulus strength needed to generate the maximum fEPSP amplitude during the I/O curve measurement. The pathway was stimulated once every 30 s until a stable baseline lasting at least 10 min was observed. Long-term potentiation (LTP) was induced in the molecular layer using 600 low-frequency stimulation pulses at 1 Hz applied for 10 min. Long-term depression (LTD) of the CF was induced using 600 low-frequency stimulation pulses at 1 Hz applied for 10 min to an electrode that evoked responses at the boundary of Purkinje and molecular layers. Next, baseline stimulation was resumed and the fEPSPs recorded for at least 60 more minutes. Finally, another I/O curve was recorded and generated as described above. For all recordings, the MED-64 system and Mobius software were used (Alpha MED Scientific, Inc.). LTP/LTD was calculated as the percent increase/decrease of the mean fEPSP descending slope (10–90 section) after 1-Hz stimulation and normalized to the mean fEPSP descending slope of baseline recordings (during 3 min prior to 1-Hz stimulus).

## Results

### Generation of the SCA34 Rat Model

**Fig. 1:**
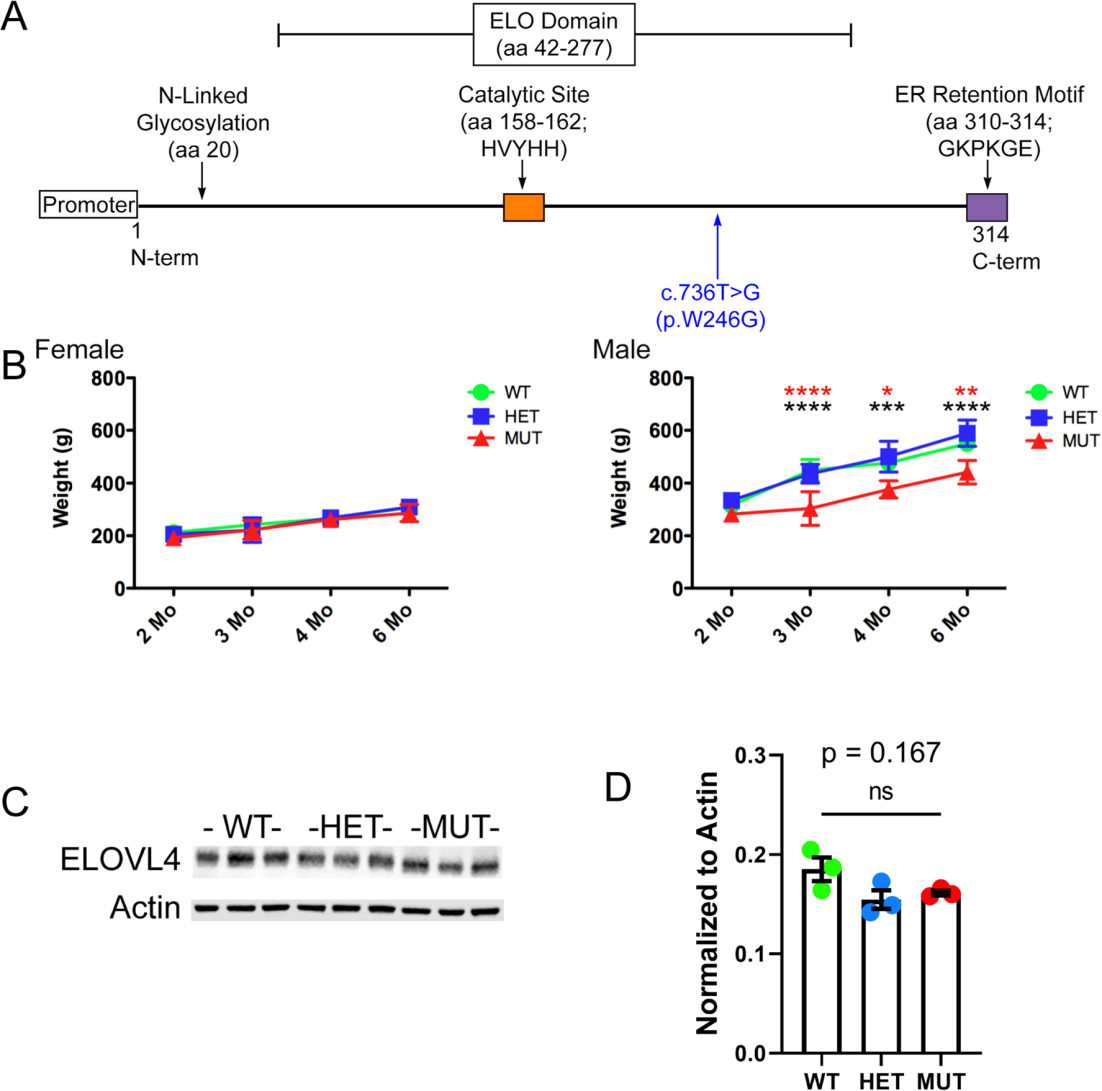
Knock-in of the W246G ELOVL4 mutation, physical differences, and ELOVL4 expression in WT, HET, and MUT rats. **A** Cartoon depicting the overall structure and functional domains of ELOVL4 and the location of the W246G ELOVL4 mutation. **B** The W246G ELOVL4 mutation does not affect the size of female rats at any age between 2 months and 6 months of age. Male MUT rats are significantly smaller than age-matched WT and HET rats from 3 months of age (females, 2 months: n = 26 WT, 22 HET, 24 MUT; 3 months: n = 17 WT, 13 HET, 17 MUT; 4 months: n = 9 WT, 9 HET, 10 MUT; 6 months: n = 17 WT, 14 HET, 10 MUT. Males, 2 months: n = 5 WT, 8 HET, 4 MUT; 3 months: n = 5 WT, 8 HET, 4 MUT; 4 months: n = 4 WT, 7 HET, 3 MUT; 6 months: n = 7 WT, 10 HET, 4 MUT. Data are shown as mean ± SD. 1-way ANOVA + Bonferroni post-hoc test. *p< 0.05; **p<0.01; ***p<0.001; ****p<0.0001). **C** Western blot of cerebellum from WT, HET, and MUT rats (n=3 each). **D** Quantification of ELOVL4 levels (normalized to Actin loading control) showed no differences across genotypes. Analysis performed on samples from 3 different rats of each genotype (data are shown as mean ± SEM. Statistical analysis by 1-way ANOVA with Tukey’s post-hoc test)

We used the CRISPR-Cas9 gene editing approach to knock in the c.736T>G (p.W246G) mutation that causes human SCA34 [16] into exon 6 of the rat *Elovl4* gene (Ensembl: ENSRNOG00000009773) to generate heterozygous Long-Evans F0 founder rats (Fig. 1A). See Agbaga et al. (2020) for a more detailed description [34]. The characterization of the model is reproduced (34) in Supplemental Figures 1-6. The F0 founder rats were bred to wild-type (WT) Long-Evans rats to establish heterozygote breeders. For consistency and clarity, this mutation is referred to as the “W246G ELOVL4 mutation.” Editing of a single copy of the WT *Elovl4* allele to the c.736T>G (p.W246G) mutant allele was confirmed by genotyping and by Sanger and whole-genome DNA sequencing, which did not find any aberrant indels, insertions, or off-target editing effects [34]. Heterozygous W246G ELOVL4 knock-in rats were fertile and passed the 736T>G allele to their offspring to produce heterozygote (HET) and homozygote (MUT) offspring. MUT rats were viable and fertile, in contrast to mice with *Elovl4* knockout or homozygous expression of STGD3 alleles, which die from dehydration shortly after birth due to loss of the skin water barrier [30–33]. Phenotypically, MUT rats presented with EKV characterized by reddened skin and patchy loss of hair [34], consistent with the finding of EKV in some human SCA34 patients [14, 15]. Age-matched female rats showed no differences in weight across genotypes from 2 to 6 months of age. In contrast, male rats exhibited a sex-related difference in weight, with male MUT rats being significantly smaller than age-matched male WT and HET rats from 2 to 6 months of age (Fig. 1B). Immunoblotting showed no differences in ELOVL4 levels in the WT, HET, and MUT cerebellum (Fig. 1C, D) using a well-characterized antibody that recognizes WT and full-length MUT ELOVL4 [1]. Finally, assessment of the effect of the W246G ELOVL4 mutation on VLC-SFA and VLC-PUFA synthesis showed that the mutation significantly reduced, but did not eliminate, VLC-SFA production in the skin (remaining 28:0 + 30:0 levels around 35% compared with WT), and had no significant effect on VLC-PUFA synthesis in the retina (34; Supplemental Figs. 5 and 6).

**Fig. 2:**
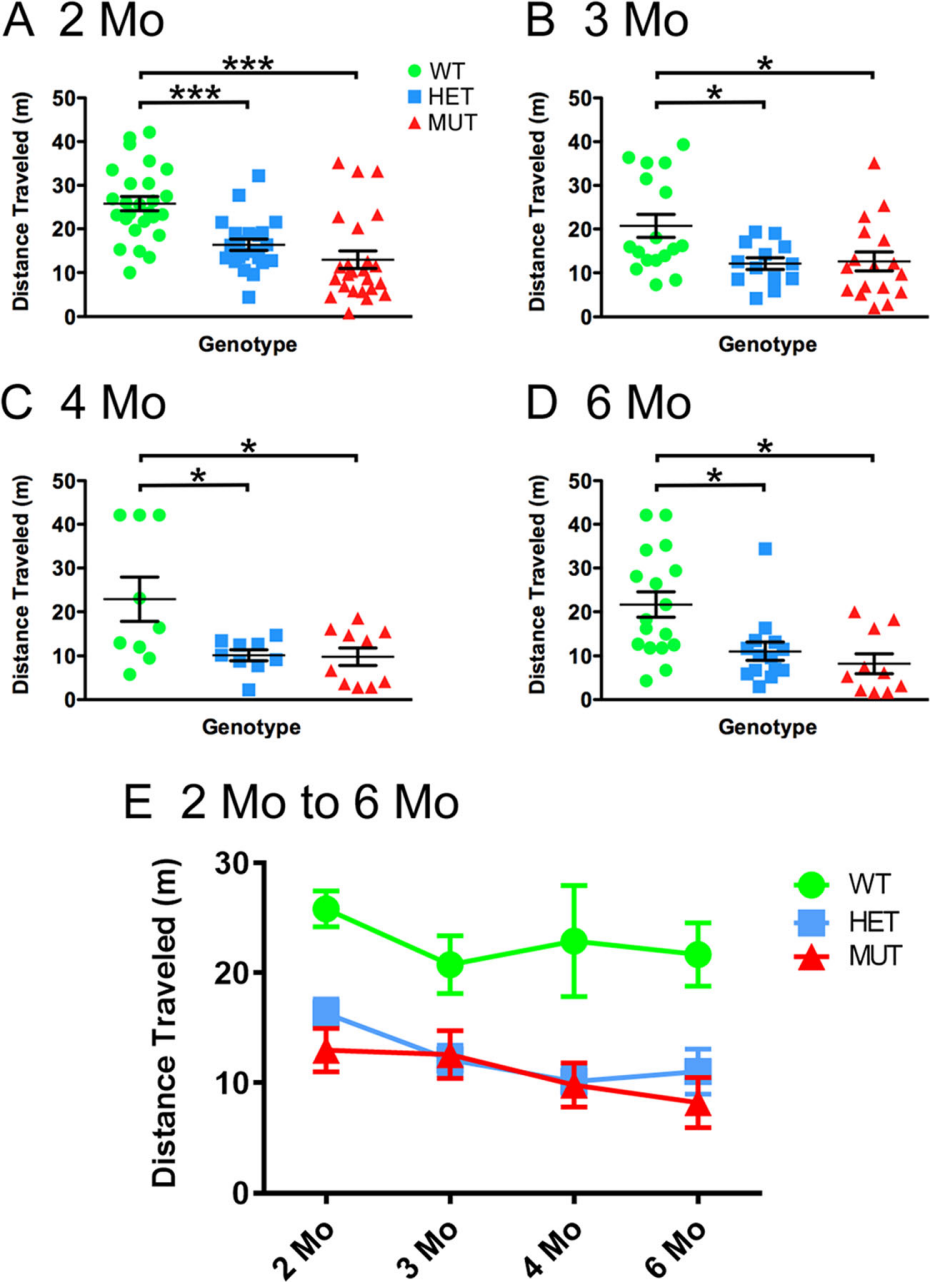
The W246G ELOVL4 mutation impaired motor function assessed by rotarod test. (A–D) Female HET and MUT rats showed consistent impairment of motor performance on the rotarod test compared to age-matched WT female rats from 2 to 6 months of age. Distance traveled variable shown. (E) Data from panels (A–D) re-plotted to show performance over time (2 months: n = 26 WT, 22 HET, 24 MUT. 3 months: n = 17 WT, 13 HET, 17 MUT. 4 months: n = 9 WT, 9 HET, 10 MUT. 6 months: n = 17 WT, 14 HET, 10 MUT; data are shown as mean ± SEM. 1-way ANOVA + Bonferroni post-hoc test. *p< 0.05; **p<0.01; ***p<0.001)

The W246G ELOVL4 mutation did not cause overt impairment of motor function, as male and female HET and MUT rats could walk, rear on their hind legs, and breed, consistent with the relatively late onset and slow progression of SCA34 in humans with the W246G ELOVL4 mutation. To better assess effects on motor performance, cohorts of female WT, HET, and MUT rats were tested at 2, 3, 4, and 6 months of age using the rotarod assay. The rotarod test revealed significant motor impairment in female HET and MUT rats compared with WT rats at all ages examined (Fig. 2), indicating that the deficits observed at 2 months were not transient. Interestingly, HET and MUT female rats performed equally poorly in the test. These studies indicate that the W246G ELOVL4 mutation caused persistent motor impairment in HET and MUT rats starting from early ages. We also tested the performance of male rats on the rotarod task at 2 and 6 months of age. Male rats of all genotypes showed poor performance at both ages (Supplemental Fig. 7). Statistical analysis showed no significant differences across male rats regardless of genotype. The reason for this sex difference in rotarod performance is unclear; however, the difference in motor behavior between male and female rats was striking and consistent.

### The W246G ELOVL4 Mutation Does Not Disrupt ELOVL4 Distribution or Cerebellar Cytoarchitecture Out to At Least 6 Months of Age

**Fig. 3:**
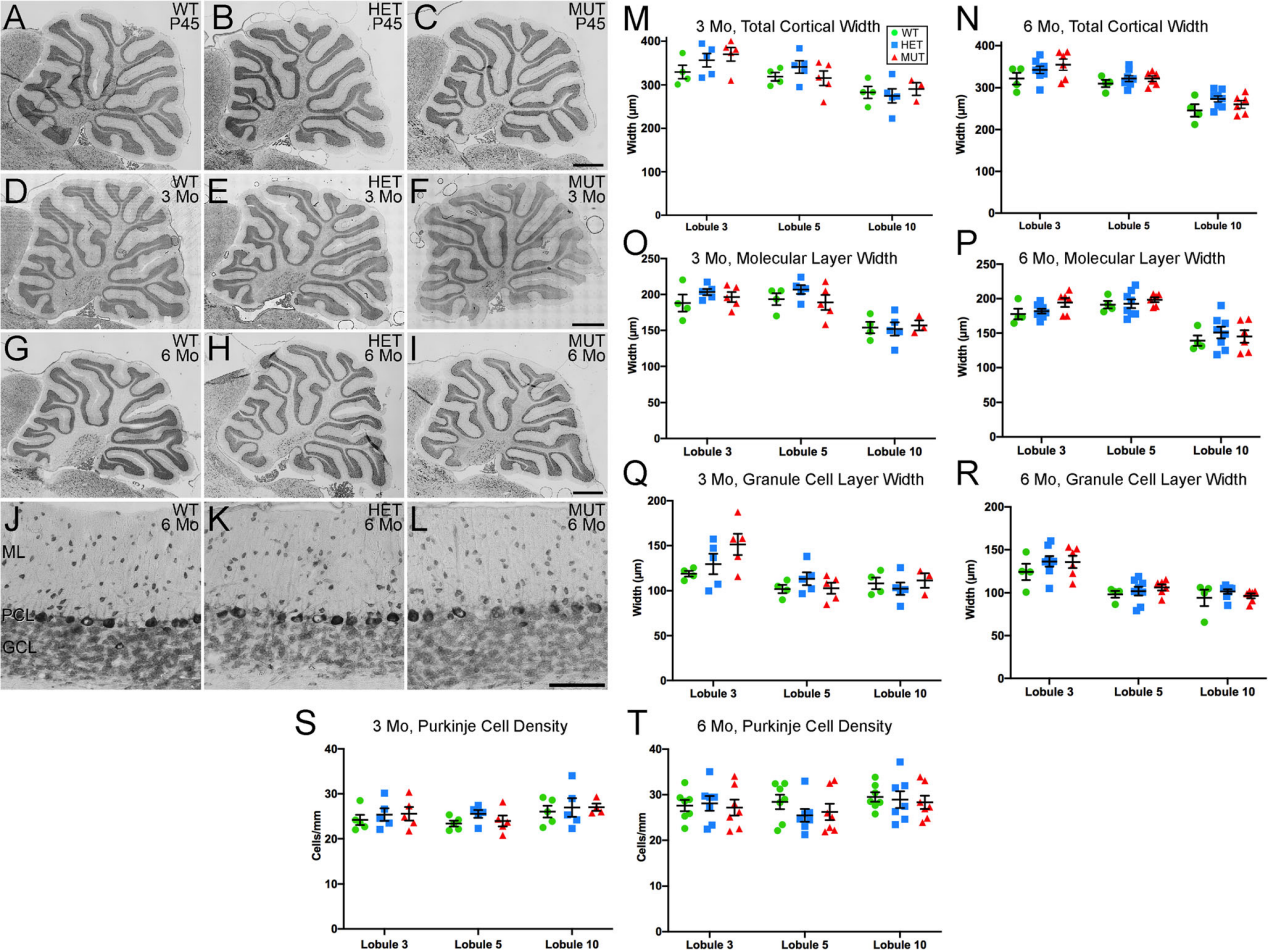
Gross cerebellar structure and morphometric analysis. (A–I) Gross cerebellar structure in WT, HET, and MUT rats at postnatal day 45 (P45), 3 months, and 6 months of age is comparable. Scale bar = 1 mm for each row. (J–L) Lamination and cellular distribution in the cerebellar cortex is normal in WT, HET, and MUT rats. Six months of age shown, scale bar = 100 μm. (M–T) Morphometric analysis of cortical layer thickness in WT, HET, and MUT rats indicates no substantial reduction in any cortical layer at 3 months or 6 months, although a significant difference in Purkinje cell layer width between WT and MUT rats was noted in lobule 3 at 6 months (3 months: n = 3–5 animals/genotype. 6 months: n = 4–8 animals/genotype. Data are shown as mean ± SEM. 1-way ANOVA with Tukey’s post-hoc test. *p< 0.05). (U, V) Linear density of Purkinje cells shows no difference among WT, HET, and MUT rats (3 months: n = 5 animals/genotype. 6 months: n = 7 animals/genotype. Data are shown as mean ± SEM. 1-way ANOVA with Tukey’s post-hoc test)

Cerebellar atrophy is a common feature of SCA34 [14, 16]. Loss of Purkinje cells in particular is a prominent feature of many forms of SCA [43]. To assess whether the W246G ELOVL4 mutation caused neurodegeneration or disrupted cerebellar cytoarchitecture, we conducted a detailed morphological assessment of the cerebellar cortex. Gross cerebellar structure was not affected by the W246G ELOVL4 mutation (Fig. 3(A–I)). Lobule organization was similar in WT, HET, and MUT rats through at least 6 months of age. The cerebellar cortex showed the normal three-layered structure comprised of a thick, outer molecular layer housing interneurons and synapses, a well-organized monolayer of Purkinje cells, and a densely packed granule cell layer adjacent to the deep white matter of the arbor vitae (Fig. 3(J–L)). Analysis of cortical layer thickness in lobules 3, 5, and 10 showed no thinning of the molecular or granule cell layers at 3 or 6 months (Fig. 3(M–R)). The linear density of Purkinje cells also showed no differences across ages or genotypes (Fig. 3(S, T)), indicating that there was no substantial loss of Purkinje cells over this time frame.

**Fig. 4:**
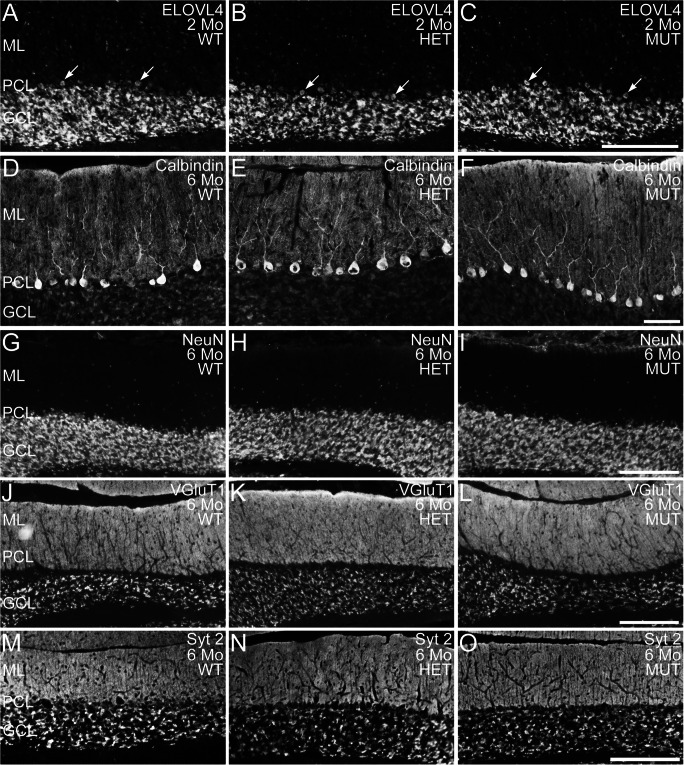
Distribution of ELOVL4 and cell- and synapse-specific markers is normal in WT, HET, and MUT rat cerebellum. (A–C) Distribution of ELOVL4 in the cerebellar cortex of WT, HET, and MUT rats is comparable. Labeling is present in granule cells in the granule cell layer (GCL), Purkinje cells (arrows) in the Purkinje cell layer (PCL), and inhibitory neurons in the molecular layer (ML). (D–F) Purkinje cells identified by labeling for calbindin are organized in a monolayer in the Purkinje cell layer (PCL) and show similar structure in the WT, HET, and MUT cerebellum. (G–I) Distribution of NeuN, a marker for granule cells in the granule cell layer (GCL), is comparable across all genotypes. A small population of NeuN-positive cells is also present in the molecular layer (ML), as appropriate. (J–L) Distribution of Vesicular Glutamate Transporter 1 (VGluT1), a marker for excitatory parallel fiber and mossy fiber synapses, is comparable across all genotypes. (M–O) Distribution of synaptotagmin 2 (Syt2), a marker for inhibitory synapses, is comparable across all genotypes. Scale bars = 200 μm for each row

**Fig. 5:**
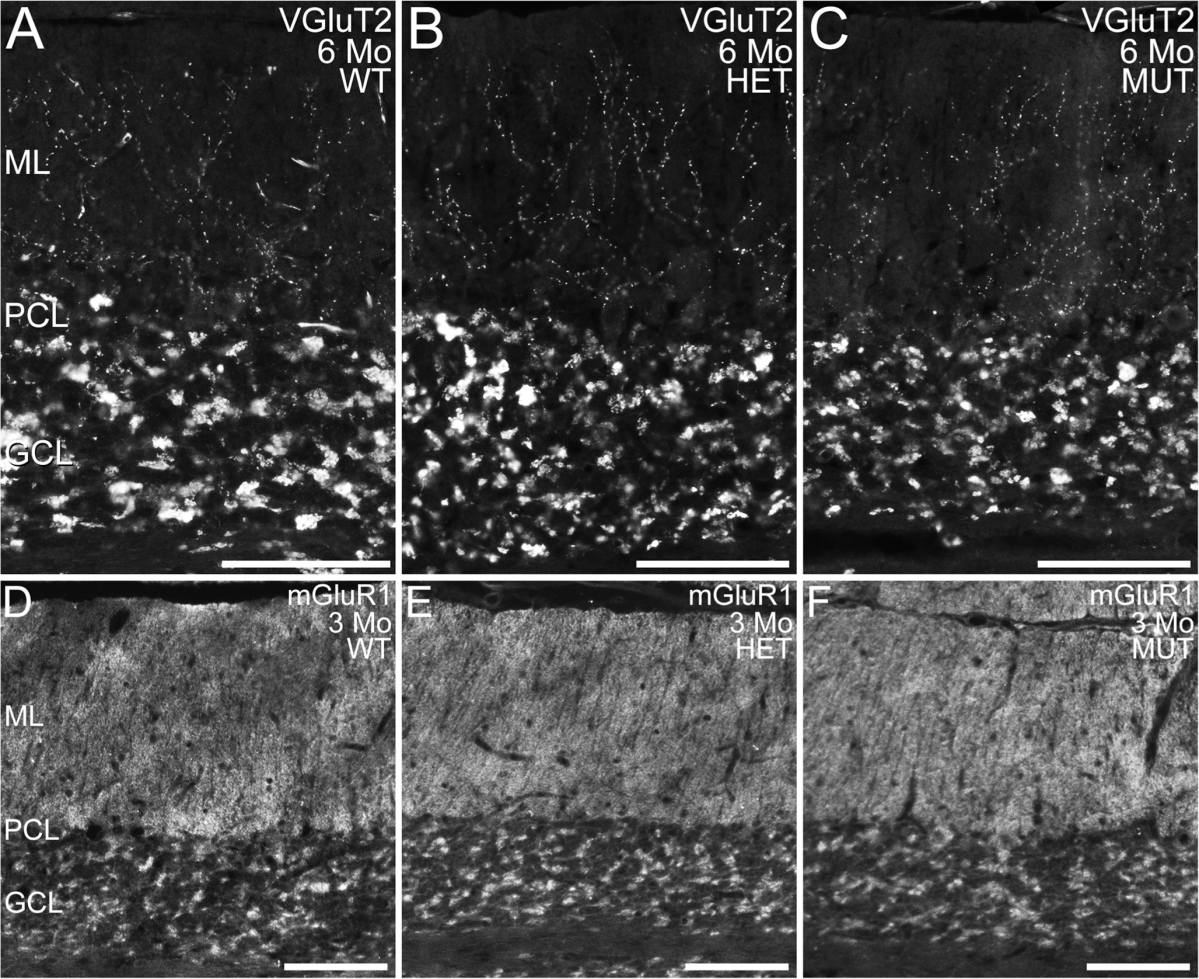
Distribution of VGluT2 and mGluR1 in WT, HET, and MUT rat cerebellum. (A–C) Distribution of VGluT2, a marker for climbing fiber synapses, is comparable in the cerebellar cortex of WT, HET, and MUT rats. Labeling is present in climbing fiber synapses onto Purkinje cell dendrites in the molecular layer (ML) and in the synaptic glomeruli of the granule cell layer (GCL). PCL, Purkinje cell layer. (D–F) Distribution of mGluR1 is comparable in the WT, HET, and MUT cerebellum. PCL, Purkinje cell layer. Scale bars = 100 μm for panels (A–C); 200 μm for panels (D–F)

To further assess cerebellar integrity, immunolabeling for ELOVL4 and a variety of well-known cell- and synapse-specific markers was performed (Fig. 4). Notably, as the ELOVL4 antibody recognizes both wild-type and mutant protein, we were able to detect the distribution of WT and W246G mutant ELOVL4 protein using the same antibody (see Fig. 1). The W246G ELOVL4 mutation did not alter ELOVL4 distribution in the cerebellum. ELOVL4 was present in granule cells, inhibitory interneurons in the molecular layer, and Purkinje cells in the cerebellum of rats of all genotypes (Fig. 4(A–C)), similar to ELOVL4 distribution in the mouse cerebellum [37]. Purkinje and granule cells labeled for calbindin and NeuN, respectively, showed normal morphology and distribution in WT, HET, and MUT rats at all ages examined (Fig. 4(D–I); Supplemental Figs. 8, 9). Labeling for VGluT1, a marker for mossy and parallel fiber synapses, and synaptotagmin 2, a marker for GABAergic synapses, both showed numerous synapses throughout the molecular layer and in the synaptic glomeruli of the granule cell layer in all genotypes at all ages, as appropriate (Fig. 4(J–O); Supplemental Figs. 10, 11). Labeling for VGluT2, a specific marker for climbing fiber synapses onto Purkinje cells, confirmed that climbing fiber synapses were present in the molecular layer of WT, HET, and MUT cerebellar cortex, as expected (Fig. 5). The distribution of mGluR1 [44], which is critical to Purkinje cell synaptic plasticity [45], was unaffected by the W246G ELOVL4 mutation (Fig. 5). Similarly, Protein Kinase C gamma (PKCɣ) and Inositol Phosphate 3 Receptor (IP3R), which are also important to Purkinje cell synaptic plasticity [46, 47], showed no substantial differences in distribution or expression levels in the cerebellum (Supplemental Figs. 12, 13). Labeling for astrocytes and microglia showed the normal distribution and morphology of these cell populations across genotypes of all ages, with no sign of overt neuroinflammation (Supplemental Figs. 14, 15).

**Fig. 6:**
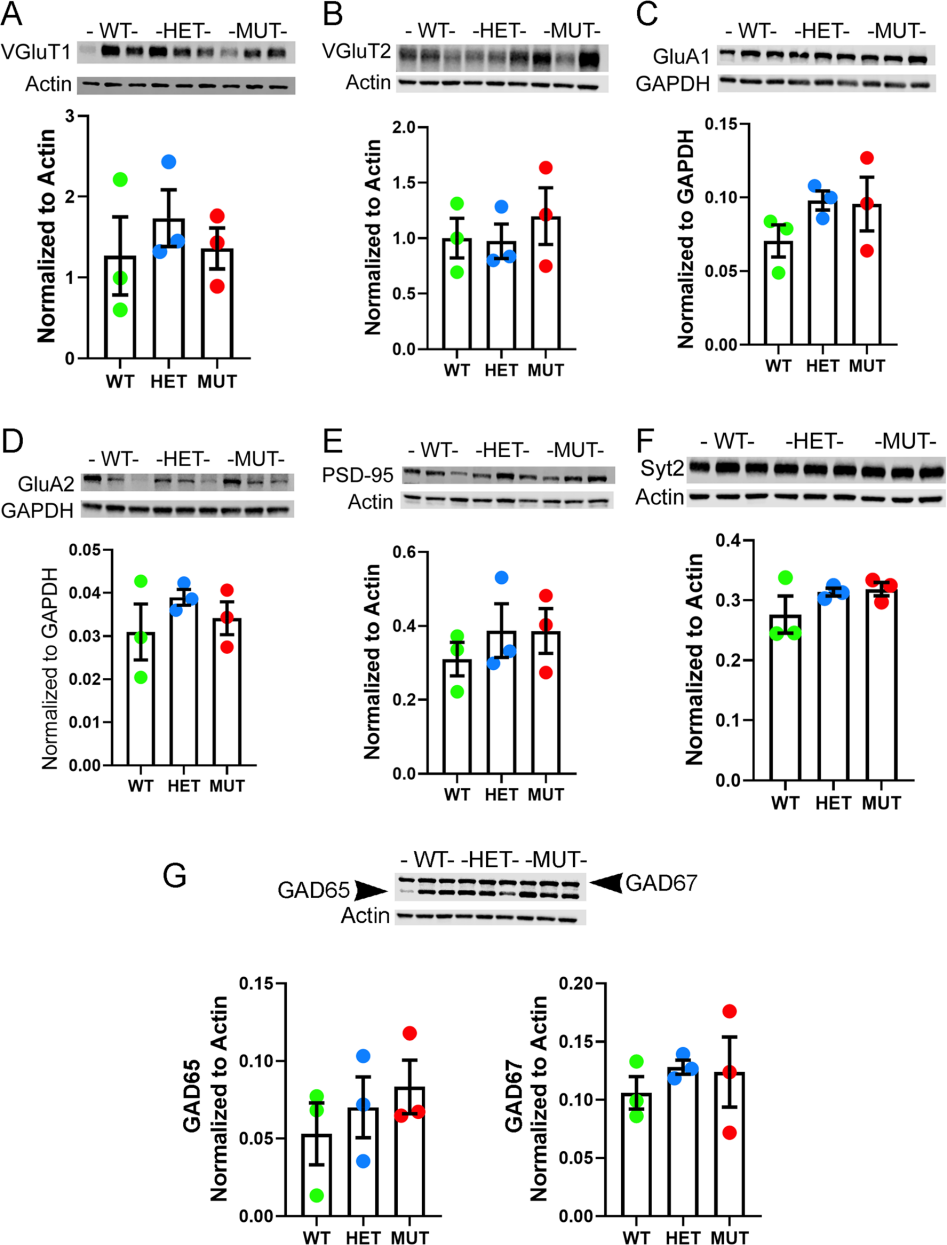
The W246G ELOVL4 mutation does not significantly alter levels of synaptic proteins in the WT, HET, and MUT cerebellum. Immunoblots for **A** VGluT1, **B** VGluT2, **C** GluA1, **D** GluA2, **E** PSD-95, **F** Syt 2, and **G** GAD-65 and GAD-67 are shown along with their quantification (normalized to loading control: Actin or GAPDH). Quantitation was performed on samples obtained from 3 different rats of each genotype (data are shown as mean ± SEM. Statistical analysis by 1-way ANOVA with Tukey’s post-hoc test)

To further assess the effects of the W246G ELOVL4 mutation at the synaptic level, western blot analysis of several important synaptic proteins associated with glutamatergic and GABAergic synapses was performed (Fig. 6). We examined the following proteins: excitatory presynaptic markers—VGluT1, VGluT2; AMPA glutamate receptors—GluA1, GluA2, and the postsynaptic density protein PSD-95; and inhibitory synaptic markers—Syt2, GAD65, and GAD67. No significant differences were present among WT, HET, and MUT cerebellum for any of the glutamatergic and GABAergic synaptic proteins examined.

Together, these data indicate that the motor deficits observed in HET and MUT rats did not arise from any gross disruption of the cellular or synaptic organization of the cerebellum or neurodegeneration.

### The W246G ELOVL4 Mutation Alters Neuronal Network Communication in the Cerebellar Cortex

**Fig. 7:**
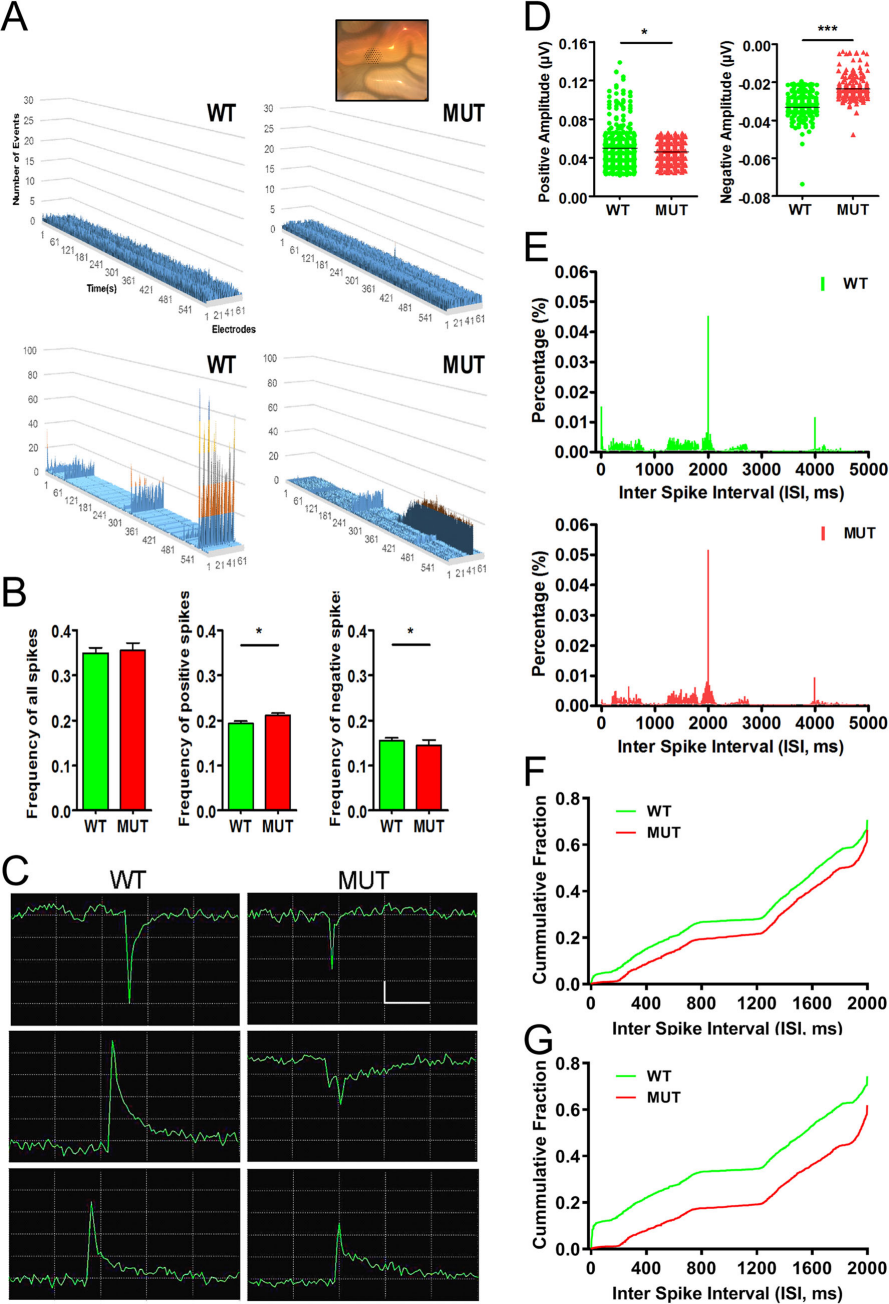
Decreased amplitude and frequency domain shift of spontaneous activity in MUT cerebellar slices. **A** Representative plots of spontaneous network activity in WT (left panel) and MUT (right panel) cerebellar slices recorded from 61 locations (*X* axis = time, *Y* axis = number of events, *Z* axis = recording electrode #). WT and KI slices show very low activity (upper panels) or high activity (lower panels). Inset shows a picture of the cerebellar slice on the hexagonal multi-electrode array. **B** No difference in the average frequency of spontaneous network activity in the cerebellum of WT (n=7 slices from 5 rats) and MUT (n=8 slices from 6 rats). Note an increased average frequency of positive spikes and reduced frequency of negative spikes in the MUT cerebellum (shown as mean ± SEM, analysis by Mann-Whitney test. *p<0.05). **C** Representative traces from spontaneous events of WT (left panel) and MUT (right panel). Note higher amplitude in positive spikes (lower panel) and negative spikes (upper panel) of the WT traces. An extremely large positive spike in WT and a complex spike in MUT are shown in middle panel traces. Scale bars *y*=20 μV, *x*=1 ms. **D** Analysis of spontaneous event amplitudes. Note the decreased amplitude of positive spikes (left panel, *p<0.05, Wilcoxon signed rank test) and decreased amplitude of negative spikes (right panel, ***p<0.001, Wilcoxon signed rank test) in the MUT compared with WT cerebellum. **E** Resting spontaneous activity in WT and MUT slices. Distribution of interspike interval (ISI) in 5 ms bins from WT (upper; n=7) and MUT (lower; n=8) slices during 10-min recordings. **F** Cumulative histogram of ISI analysis of events with 2 s or less time between them. Data shown in **E** re-analyzed using 1-ms bins. MUT slices show-reduced activity at high frequencies (above 20 Hz) compared with WT slices (p<0.0001, Mann-Whitney test). ***G*** Cumulative histogram of ISI of spontaneous activity in PF–PC connections in WT (n=4) and MUT slices (n=5) analyzed using 1-ms bins (p<0.0001, Mann-Whitney test)

Cerebellar cortical function is essential for motor learning, and it is thought to involve fine tuning of the extensive PF-Purkinje cell synaptic network in coordination with the “error reporting” of CF-Purkinje cell input [48]. To better understand the origins of the motor impairment associated with the W246G ELOVL4 mutation, we assessed neuronal network properties in the cerebellum using multi-electrode array electrophysiological recording from cerebellar slices [49] prepared from WT and MUT rats (Fig. 7, see Methods for details). Briefly, an array of 61 electrodes arranged hexagonally at 70-μm intervals was carefully positioned under the slice to cover all cerebellar cortical layers. The overall spontaneous network activity was recorded for 10 min under resting conditions [3]. Assessing entire 10-min-long recordings and plotting the events per second for all 61 electrodes revealed two major differences in network activity in WT and MUT slices (Fig. 7A). First, slices generally showed either very low activity (less than average of 1 event per sec, upper panels) or high activity (lower panels on Fig. 7A). There was no significant difference between WT and MUT slices in the distribution of low to high activity (20/9 for WT and 15/10 for MUT, chi-square value is 0.4732, p=0.491511). However, there was a striking failure of PF synchronization in MUT slices compared with the almost uniform synchronization of spontaneous activity in WT slices recorded on all electrodes simultaneously (Fig. 7A, lower two panels). Interestingly, in many MUT slices, we detected robust, persistent activity (Fig. 7A, lower two panels). Next, we analyzed spike frequency, amplitude, and interspike interval (ISI) from these recordings. Although average spike frequency did not differ in the cerebellar cortex between WT and MUT slices (Fig. 7B left panel), further analysis revealed an increased average frequency of positive spikes (Fig. 7B middle panel) and a reduced frequency of negative spikes in the MUT cerebellum (Fig. 7B right panel). In addition, the amplitude of network events was significantly decreased in MUT cerebellar slices compared with WT (Fig. 7C, negative spike traces and positive spike traces; and Fig. 7D). The overall distribution of events as shown by the ISI histogram was similar in WT and MUT slices (Fig. 7E, p=0.219, Wilcoxon ranked test), with both showing peak activity at two frequencies of 0.5 Hz and 0.26 Hz. We noticed more activity at the highest frequencies in the WT slices and selectively analyzed the 1 Hz or higher activity signals (Fig. 7F). Indeed, MUT cerebellar slices had a marked reduction in activity above 20 Hz (ISI <0.05 s; Fig. 7F; p<0.0001, Mann-Whitney test). Next, to determine whether PF activity was altered under resting conditions, we analyzed the spontaneous activity from the same electrodes that were used later to record PF LTP. Activity in MUT PF differed from WT PF: the difference was mostly in the high >20-Hz frequencies, but interestingly, the 0.6–4-Hz activity corresponding to delta band was also reduced (Fig. 7G; p<0.0001, Mann-Whitney test). These observations from the spontaneous network activity indicate that W246G ELOVL4 mutation alters and desynchronizes neuronal network communication in the cerebellar cortex.

### The W246G ELOVL4 Mutation Severely Impairs Synaptic Plasticity at PF and CF Inputs to Purkinje Cells

Differences in neuronal network activity between WT and MUT cerebellum as well as the motor learning deficits of HET and MUT rats in the absence of neurodegeneration are consistent with the notion that synaptic function is impaired by the W246G ELOVL4 mutation. Therefore, we tested whether the W246G ELOVL4 mutation affected neurotransmission and long-term synaptic plasticity in the cerebellum. Long-term potentiation (LTP) and long-term depression (LTD) are hours-long increases and decreases, respectively, in synaptic strength in response to strong synaptic stimulation, and are important to motor learning. ELOVL4 is highly expressed in cerebellar granule cells (Fig. 4) and cells in the inferior olivary complex [37], and its VLC-SFA products that are incorporated into sphingolipids are known to modulate synaptic release in hippocampus [3]. To assess the functional effects of the W246G ELOVL4 mutation on long-term synaptic plasticity, we tested the function of PF and CF inputs to Purkinje cells in cerebellar slices prepared from 3- to 4-month-old WT and MUT rats.

**Fig. 8:**
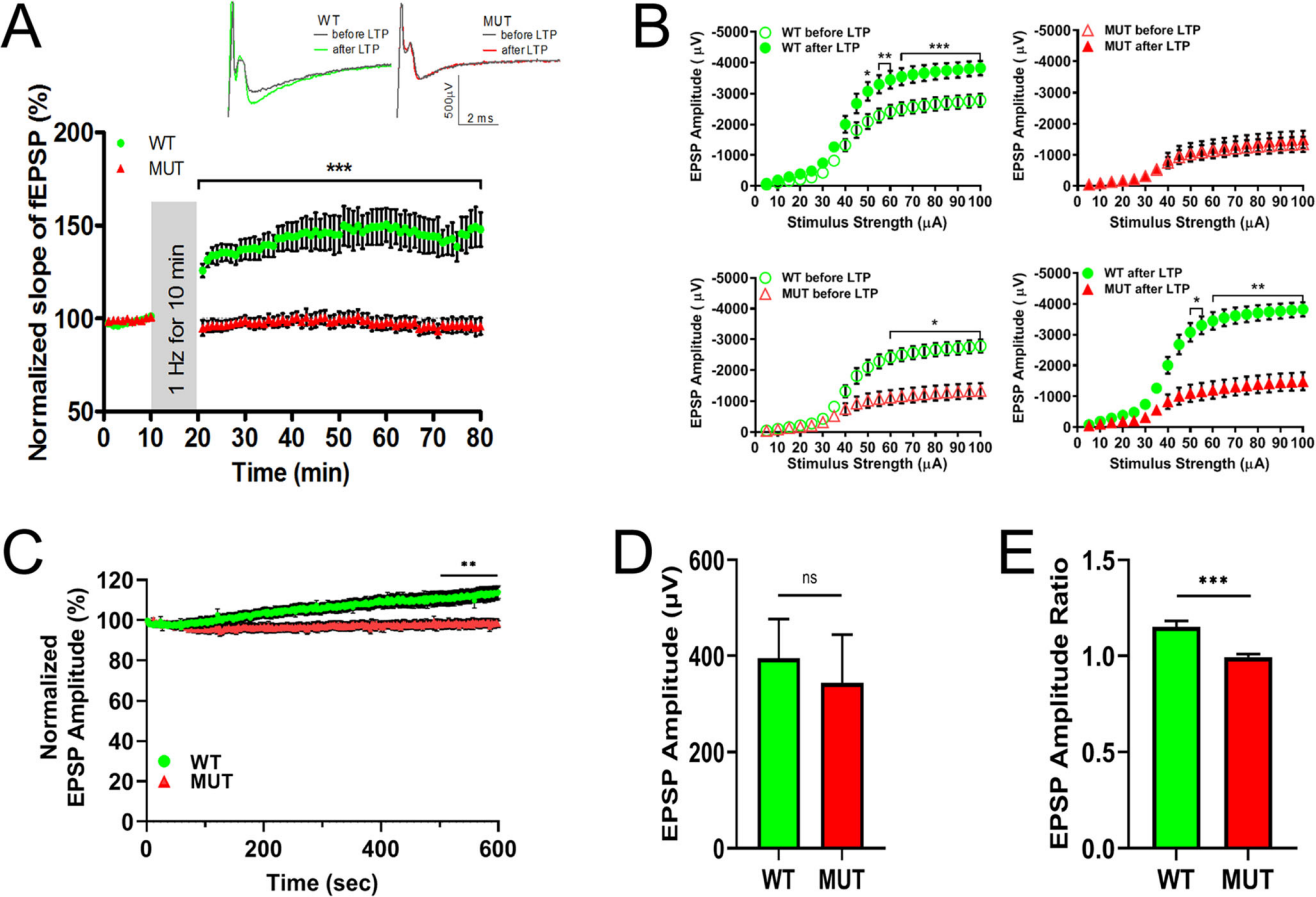
LTP at PF-Purkinje cell synapse is impaired in MUT rats. **A** LTP induced in WT rats by stimulation of the PFs in the molecular layer at 1 Hz for 10 min induces LTP in WT rat cerebellar slices (green circles, n = 14 slices from 6 rats), but not in cerebellar slices from MUT rats (red triangles, n = 16 slices from 6 rats). Each data point represents the average of two successive test responses. The vertical gray bar indicates the period of 1-Hz stimulation. Typical traces show EPSPs before (black line) and after (green line for WT, red line for MUT) 1-Hz stimulation in slices from WT (left) and MUT (right) rats (data are shown as mean ± SEM; ***p < 0.001, two-way repeated measures ANOVA with Bonferroni post hoc test [RM ANOVA]). **B** Baseline and potentiated synaptic strength in PF-PC synapses. Graph depicts amplitude of fEPSP evoked from WT slices (green, n=14 from 6 rats) and MUT slices (red, n=13 from 6 rats) by stepwise increase in the stimulus from 5 to 100 μA. WT slices show increased amplitudes after 1 h LTP (upper left panel, ***p < 0.001, two-way RM ANOVA). MUT slices show no increase in amplitudes after 1-h LTP (upper right panel, ns). Comparison of amplitudes between WT and MUT slices before LTP (lower left panel, *p < 0.05, two-way RM ANOVA) and after LTP (lower right panel, **p < 0.01, two-way RM ANOVA). **C** Changes in the synaptic responses during the 1-Hz stimulation trains. Normalized fEPSP amplitude in WT slices (green circles, n=14 slices from 6 rats) increased significantly compared with MUT slices (red triangles, n=14 slices from 6 rats). Each data point represents one fEPSP response as recorded every second and normalized to the amplitude of first response (data are shown as mean ± SEM, **p < 0.01, two-way ANOVA, last 100 s). **D** fEPSP average amplitude of the first response in WT and MUT slices (same data set as panel **C**; shown as mean ± SEM, ns, Mann-Whitney test). **E** Ratio of average amplitude of the last five responses (#596–600) divided by the average of the first five responses (#1–5) in WT and MUT slices (data are shown as mean ± SEM, ***p < 0.001, Mann-Whitney test)

The most numerous excitatory synaptic inputs to Purkinje cells are from PFs, which drive simple spikes in Purkinje cells [50]. To assess the function of PF-Purkinje cell synapses, acute ex vivo cerebellar slices from WT or MUT rats were stimulated using one electrode located in the molecular layer to stimulate PFs. The field potentials of excitatory post-synaptic potential (fEPSP) responses in the molecular layer were recorded every 30 s using a MED64 multi-electrode array system (MEA). A stimulation paradigm of 1 Hz for 10 min was used to induce PF LTP (modified from [51–54]). This stimulation paradigm does not require CF co-stimulation and indeed induced robust LTP in slices from WT, but not MUT, rats (Fig. 8). As expected, WT slices showed a significant increase in the slope of PF fEPSP 1 h after stimulation. However, no increase in the PF fEPSP slope was present in slices from MUT rats, indicating severe impairment of LTP at PF-Purkinje cell synapses (Fig. 8A, upper panel, WT = 145.4 ± 9.2% normalized slope, n=14 slices from 6 rats; MUT = 96.2 ± 3.8%, n=16 slices from 6 rats; mean ± SEM; ***p < 0.001, two-way repeated measures ANOVA with Bonferroni post-hoc test [RM ANOVA]). We also measured the basic physiological properties of the LTP at PF-Purkinje cell synapses by recording the characteristics of synaptic responses to a range of increasing stimuli (5–100 μA) before and after recording LTP. This “Input–Output” test allowed us to assess general physiological properties of PF-Purkinje cell synapses and to evaluate how LTP affects them. We found that, at stimulus intensity greater than 60 μA, MUT slices showed a significantly smaller fEPSP amplitude than WT slices before LTP (Fig. 8B; lower left panel, *p < 0.05, two-way RM ANOVA). Furthermore, after LTP induction, while the response for WT slices increased significantly (Fig. 8B; upper left panel, ***p < 0.001, two-way RM ANOVA), MUT slices showed no change in response (Fig. 8B; upper right panel, ns, two-way RM ANOVA), indicating that MUT animals had severely impaired synaptic plasticity. Accordingly, the difference between WT and MUT slices in synaptic response to stepwise stimuli became even more pronounced after LTP was induced (Fig. 8B; lower right panel, **p < 0.01, two-way RM ANOVA).

As the baseline synaptic strength differed between WT and MUT PF fiber recordings, we next scrutinized the synaptic responses during the 1-Hz stimulus trains. We found that the applied low-intensity stimuli evoked similar responses from both genotypes (Fig. 8D), as the average amplitude of the first response was 395 ± 81.6 μV for WT and 344.3 ± 100 μV for MUT (p=0.66, Mann-Whitney test). During the stimulus train, WT responses increased significantly, but MUT responses remained stable (Fig. 8C, **p < 0.01, two-way ANOVA, last 100 s). This also was reflected in the ratio of average amplitude of the last five responses divided by the average of the first five responses (Fig. 8E), which increased during the 1-Hz stimulation only in WT slices (***p < 0.001, Mann-Whitney test). Taken together, these observations support the conclusion that PF synapses with the W246G ELOVL4 mutation are selectively defective in LTP, although they respond perfectly well to low-intensity single stimulation. Importantly, we tested for any sex-related differences in parallel fiber synaptic plasticity and found that both males and female MUT rats had similar LTP impairment (Suppl. Fig. 16). Thus, we have combined the data for both sexes in Fig. 8.

**Fig. 9:**
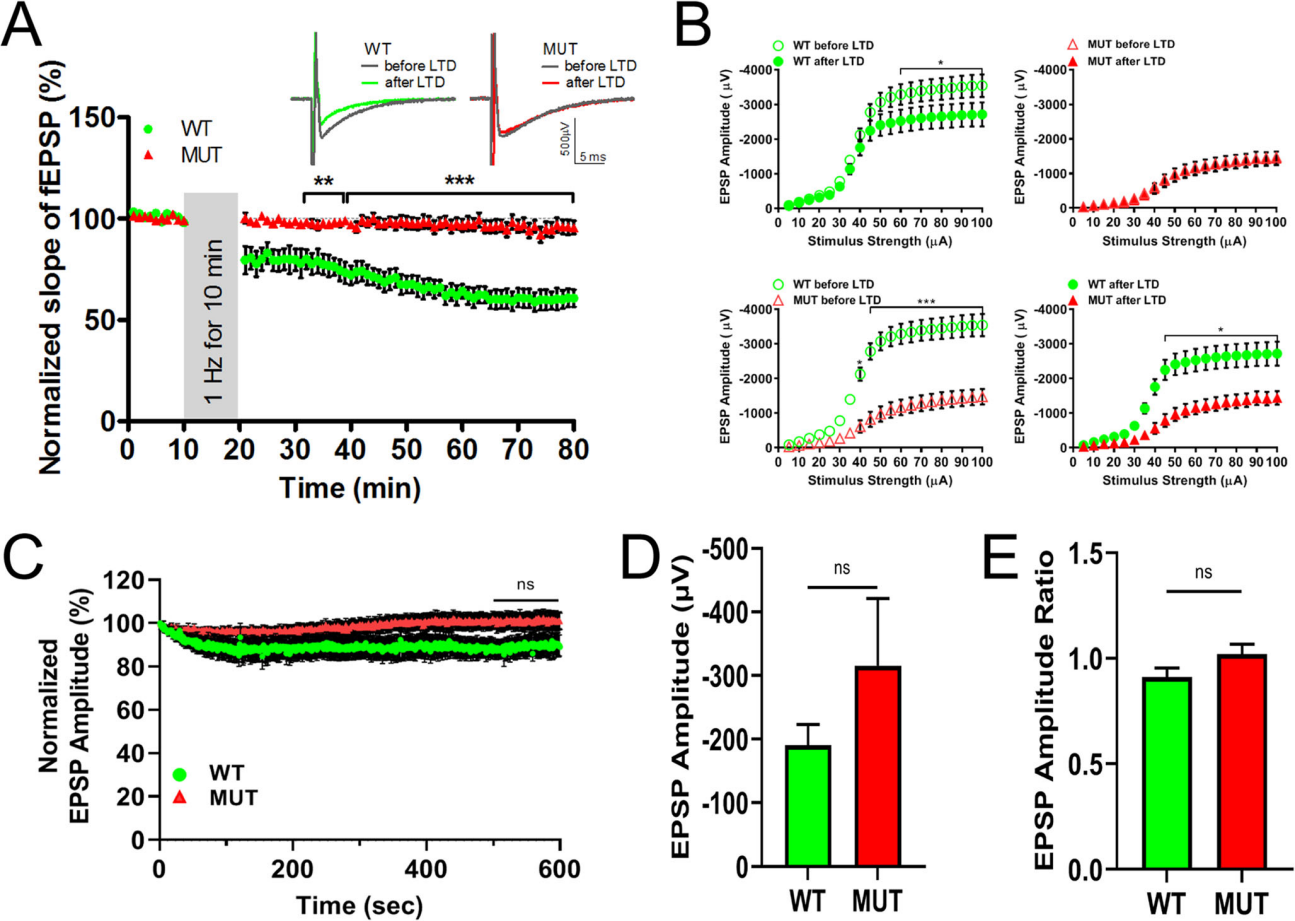
LTD at CF-PC synapses is impaired in MUT rats. **A** Stimulating the CFs in the granule cell layer at 1 Hz for 10 min induces LTD in WT cerebellar slices (green circles, n = 10 slices from 5 rats), but not in cerebellar slices from MUT rats (red triangles, n = 14 slices from 6 rats). Each data point represents the average of two successive test responses. The vertical gray bar indicates the period of 1-Hz stimulation. Representative traces depict fEPSPs before (black line) and after (green line for WT and red line for MUT) application of the 1-Hz LTD protocol in WT and MUT slices (left and right inset, respectively) (data are shown as mean ± SEM; **p < 0.01, ***p < 0.001, two-way repeated measures ANOVA with Bonferroni post hoc test [RM ANOVA]). **B** Baseline and depressed synaptic strength in CF-PC synapses. Graph depicts amplitude of fEPSP evoked from WT slices (green, n=8 from 5 rats) and MUT slices (red, n=12 from 6 rats) by stepwise increase in the stimulus from 5 to 100 μA. Note the decreased baseline CF synaptic strength and lack of LTD in MUT slices after 1-Hz stimulus. WT slices show decreased amplitudes after 1-h LTD (upper left panel, *p < 0.05, two-way RM ANOVA). MUT slices show no changes in amplitudes after 1-h LTD (upper right panel, ns). Comparison of amplitudes between WT and MUT slices before LTD (lower left panel, ***p < 0.001, two-way RM ANOVA) and after LTD (lower left panel, *p<0.05, two-way RM ANOVA). **C** Changes in the synaptic responses during the 1-Hz stimulation trains. fEPSP amplitude in WT slices (green circles, n=10 slices from 5 rats) did not change significantly compared with MUT slices (red triangles, n=12 slices from 6 rats). Each data point represents one fEPSP response as recorded every second and normalized to the amplitude of first response (data are shown as mean ± SEM, ns, two-way ANOVA, last 100 s). **D** Average amplitude of the first fEPSP response in WT and MUT slices (data are shown as mean ± SEM, ns, Mann-Whitney test). **E** Ratio of average amplitude of the last five fEPSP responses (#596–600) divided by the average of the first five responses (#1–5) in WT and MUT slices (data are shown as mean ± SEM, ns, Mann-Whitney test)

A second key excitatory synaptic input to the Purkinje cells comes from the CFs, which drive complex spikes in Purkinje cells and are critical to motor learning [50]. The CF-Purkinje cell synapse has a high release probability [55] and shows pronounced LTD in response to tetanic stimulation. To assess the function of CF-Purkinje cell synapses, slices from WT or MUT rats were given tetanic stimulation to activate CFs at low frequency (1 Hz for 10 min) and CF fEPSP responses were recorded at the boundary of Purkinje cell and molecular layers every 30 s using the MEA system (modified from [51]). This stimulation paradigm induced strong LTD in slices from WT rats as expected, but did not induce LTD in slices from MUT rats (Fig. 9A). The level of depression was significantly higher in slices from WT rats than in slices from MUT rats 1 h after induction, indicating severe impairment in LTD in the presence of the ELOVL4 W246G mutation (WT = 60.5 ± 4.6% normalized slope, n=10 recordings from 5 rats; MUT = 95.98 ± 3.4%, n=14 recordings from 6 rats; mean ± SEM; ***p < 0.001, two-way RM ANOVA). We also recorded the Input–Output characteristics of synaptic responses to increasing stimuli (5–100 μA) before and after recording LTD to measure physiological properties of CF to PC synapses. We found that, at stimulus intensity greater than 50 μA, MUT slices showed a significantly smaller fEPSP amplitude than WT slices before LTD (Fig. 9B; lower left panel, ***p < 0.001, two-way RM ANOVA). However, after LTD induction, while the response for WT slices decreased significantly (Fig. 9B; upper left panel, *p < 0.05, two-way RM ANOVA), MUT slices showed no change in response (Fig. 9B; upper right panel, ns, p > 0.05, two-way RM ANOVA), indicating that MUT animals had impaired LTD. Moreover, after LTD induction, we found that WT and MUT slices still responded differently to strong stimuli (>50 μA), with MUT slices showing a significantly smaller amplitude than WT slices (Fig. 9B; lower right panel, *p < 0.05, two-way RM ANOVA). Finally, we analyzed the synaptic responses from CF during the 1-Hz stimulation (Fig. 9C–E), as described above for PF recordings. No significant difference was present between WT and MUT slices during the 600 pulses at 1 Hz.

Importantly, we tested for any sex-related differences in synaptic plasticity of climbing fibers and found that both male and female MUT rats had similar LTD impairment (Suppl. Fig. 16). Thus, we have combined the data for both sexes in Fig. 9. Sex was not a differentiating factor in WT rat synaptic physiology either, as presented in Suppl. Fig. 17.

These experiments together indicate that the W246G ELOVL4 mutation affected both LTP at PF-Purkinje cell synapses and LTD at CF-Purkinje cell synapses, which are thought to play a central role in cerebellar learning [45, 48, 53]. In view of no changes in the major synaptic signal transduction pathways in this rat model of SCA-34 analyzed so far, further studies should address the specific role of ELOVL4 in cerebellar synaptic plasticity and the cause of ataxia.

## Discussion

These studies establish the W246G ELOVL4 knock-in rat as a model of ELOVL4 mutations that cause autosomal dominant SCA34 in humans. Importantly, heterozygous inheritance of the W246G ELOVL4 mutation results in motor deficits, mimicking the autosomal dominant inheritance pattern of human SCA34. These studies also demonstrate that homozygous inheritance of SCA34-causing mutations in ELOVL4 leads to motor impairment similar to that in heterozygotes, rather than the much more severe neuro-ichthyosis that arises from homozygous inheritance of STGD3-causing mutations in exon 6 [3] or recessive ELOVL4 mutations that cause early truncation of the protein [18, 19]. Our electrophysiological studies reveal severe impairment of long-term synaptic plasticity at PF and CF inputs to Purkinje cells, in agreement with our previous findings that deletion of ELOVL4 alters synaptic transmission in hippocampal neurons [3]. Neuroanatomical studies indicate that motor and synaptic impairments arise prior to any significant neurodegeneration, suggesting that SCA34 originates from synaptic dysfunction rather than neurodegeneration.

### Motor Impairment Does Not Arise from Disrupted Cerebellar Cytoarchitecture or Early Neurodegeneration

Motor impairment is the hallmark of SCA34. Motor learning deficits were evident in HET and MUT rats on the rotarod test, consistent with ataxia observed in human SCA34 patients. However, the appearance of these deficits by 2 months of age was unexpected as human patients with the W246G ELOVL4 mutation have an average age of disease onset ranging from the third to sixth decade [16], similar to the SCA34-causing L168F and I171T mutations in ELOVL4 [14, 17]. However, other SCA34-causing extremely rare mutations (Q180P, T233M) lead to earlier disease onset in the mid-teens or early twenties [12, 13].

Morphometric measurements in the cerebellar cortex showed no evidence of atrophy by six months of age, well after the appearance of motor deficits in these animals. Similarly, extensive morphological and immunolabeling analyses revealed no gross disruptions in the layering, cellular organization, or synaptic organization of the cerebellum in HET or MUT rats out to at least 6 months of age. These studies provide strong evidence that the motor and synaptic impairments observed in HET and MUT rats precede any substantial neurodegeneration. Early functional impairment prior to neurodegeneration has been reported in a mouse model of SCA38 with homozygous knockout of *ELOVL5*, with motor impairment appearing by 3 months of age, followed by thinning of the cerebellar cortex by 6 months of age, without Purkinje cell loss out to at least 18 months of age [56]. Early appearance of motor impairment prior to neurodegeneration has also been reported in animal models of SCA6 [57, 58].

### Relationship of ELOVL4 Function to Functional Synaptic Impairment

The different diseases caused by the various *Elovl4* mutations are likely to arise from aberrant synthesis of its VLC-SFA and VLC-PUFA products. Previous studies of the STGD3-causing 5 base-pair deletion of *Elovl4* [9, 59] showed that this mutation impairs synthesis of both VLC-SFA in skin [30–33] and VLC-PUFA in retina [32, 60, 61]. Our previous lipid analyses of baboon hippocampus showed that the 28:0 and 30:0 VLC-SFA products of ELOVL4 are incorporated into complex sphingolipids and are enriched selectively in synaptic vesicles [3]. No VLC-PUFA were detected in these studies. Studies performed using *S*^*+*^*Elovl4*^*mut/mut*^ mice (5 bp deletion that causes STGD3) that express WT *Elovl4* in the skin to prevent neonatal lethality showed that 28:0 and 30:0 VLC-SFA are key modulators of hippocampal synaptic function [3]. Homozygous *S*^*+*^*Elovl4*^*mut/mut*^ mice developed intractable seizures by postnatal day 18 and died by postnatal day 21 [3], suggesting abnormal neuronal activity. This phenotype is strikingly similar to human patients homozygous for recessive *ELOVL4* mutations that cause ELOVL4-neuroichthyosis [18, 19]. Electrophysiological recording of hippocampal slices from *S*^*+*^*Elovl4*^*mut/mut*^ mice showed bursting, epileptiform spontaneous activity [3]. Hippocampal neurons cultured from *S*^*+*^*Elovl4*^*mut/mut*^ mice showed accelerated presynaptic release kinetics that could be restored to WT kinetics by supplementation of the culture medium with VLC-SFA (28:0 + 30:0) [3]. Together, these studies establish that the VLC-SFA products of ELOVL4 can modulate synaptic function.

In contrast, our recent study of the W246G ELOVL4 mutation showed that this SCA34-causing mutation selectively impaired synthesis of VLC-SFA, but not VLC-PUFA [34]. Specifically, the W246G ELOVL4 mutation reduced, but did not eliminate, synthesis of the major VLC-SFA species (28:0 and 30:0) in the skin of MUT rats compared with WT and HET rats, consistent with EKV in MUT rats. In contrast, the W246G ELOVL4 mutation did not affect VLC-PUFA levels in the retina, consistent with the absence of retinal degeneration in HET and MUT rats. Importantly, we have shown here that maximal synaptic strengths of both PF and CF are reduced in SCA34 MUT cerebellum compared with WT synaptic connections (Figs. 8 and 9, before induction input/output curves), which mirrors the change in photopic b-wave in the retina of MUT rats [34]. These findings suggest that selective decrease of VLC-SFA synthesis by the W246G ELOVL4 mutation may contribute to SCA34. Homozygous inheritance of recessive *ELOVL4* alleles that would lead to a severely truncated and presumably inactive ELOVL4 protein results in a much more severe neuro-ichthyotic disease characterized by seizures, spasticity, intellectual disability, ichthyosis, and early death [18, 19]. Similarly, homozygous inheritance of the 5 base-pair deletion, which would severely impair synthesis of both VLC-SFA and VLC-PUFA, leads to severe seizures [3] similar to the ELOVL4 neuro-ichthyotic syndrome.

### The W246G ELOVL4 Mutation Disrupts Synaptic Plasticity at Excitatory Synapses onto Purkinje Cells

The finding that the W246G ELOVL4 mutation did not lead to gross disruption of cerebellar architecture or neurodegeneration suggests that the causal insult in SCA34 is likely to be a functional synaptopathy. An early functional synaptopathy also would be consistent with our early-onset motor deficits in the absence of structural disruption of cerebellar architecture or evidence of neurodegeneration. Cerebellar slice recordings from MUT rats showed that the W246G ELOVL4 mutation decreased transmission from PFs and CFs to Purkinje cells only moderately, but severely altered plasticity of these synapses in the cerebellum. Proper synaptic strength regulation is strikingly diminished in MUT cerebellar slices; neither PF LTP nor CF LTD could be evoked there. Moreover, disruption of cerebellar cortical synchronization, especially at higher frequencies, was combined with run-away focal PF activity only in MUT slices (Fig. 7). These changes were accompanied by a downregulation of delta band activity in the PF pathway and diminished gamma activity above 20 Hz in cerebellar circuits in the MUT rats. It is noteworthy that cerebellar delta band activity can modulate prefrontal cortical execution of motor task planning [62]. The output from Purkinje cells (especially in Crus 1) is also known to represent the phase difference of prefrontal cortical and hippocampal oscillations [63]. Interestingly, *theta* band activity, known to increase precise millisecond timing of PC firing independently of inhibitory inputs [64, 65], was spared in the MUT cerebellum.

Based on these findings, we suggest that the W246G ELOVL4 mutation is likely to cause synaptic dysfunction that leads to the development of the ataxia and other CNS symptoms characteristic of SCA34. Aberrant presynaptic release from PFs and CFs could disrupt the critical synchrony of signaling between these two synaptic populations, impairing motor learning and output. Alternatively, if the inhibitory input to Purkinje cells is altered relative to PF synaptic strength, it could then cause the observed pattern of cortical desynchronization, similarly to the serious breakdown of interfocal neighborhood interactions as originally predicted by Pellionisz and Szentagothai [66].

Plasticity at CF-Purkinje cell synapses is well characterized and the changes to CF-evoked complex spikes provide critical components of cerebellar learning [67]. Our results showed that CF stimulation at 1 Hz produced robust LTD in cerebellar slices from WT rats, but no induction of LTD in slices from MUT rats. Climbing fiber stimulation alone can induce postsynaptic LTD, which requires post-synaptic activation of mGluR1, protein kinase Cɣ (PKCɣ), and Ca^2+^ influx [55, 67, 68], and in turn leads to reduced numbers of GluA2-containing AMPA receptors in the postsynaptic membrane [68] and decreased amplitude of the synaptic response. The elimination of LTD by the W246G ELOVL4 mutation is strikingly similar to the elimination of LTD reported in a number of mouse strains with knockout or loss-of-function mutations in postsynaptic mGluR1, its downstream signaling molecules PKCɣ, phospholipase C-β4, PICK1, or the GluA2-containing AMPA receptor [45, 69–73]. Furthermore, mutations in PKCɣ and mGluR1 are known to cause human SCA14 [74, 75] and SCA44 [76]. As we have not detected changes in mGluR1 expression pattern (Fig. 5), downstream disruption in the mGluR1 signaling pathway is a leading candidate to cause the absence of CF LTD in MUT rat cerebellum. One obvious signal pathway involved in this effect is the PLC-DAG-PKC pathway. Interestingly, there were no differences in expression levels of the PKC gamma in WT, HET, and MUT rats (Suppl. Fig. 14). The other branch of this signaling pathway causes the release of calcium from internal calcium stores via inositol-(1,4,5)-tris phosphate (IP3), but there was no difference in the cerebellar expression pattern or levels of the IP3 receptors in WT, HET, and MUT rats (Suppl. Fig. 15). Other SCA models show similar CF deficits and disruption of glutamate transmission, leading to PC dysfunction [77]. Because CF LTD induction depends on an increase of postsynaptic Ca^2+^, a reduced pool of available Ca^2+^ in MUT cerebellum also could result in failure of LTD induction. Alterations in Ca^2+^ transients could have important functional roles [55, 78], as CF LTD decreases CF-evoked Ca^2+^ transients in Purkinje cells [78]. Thus, reduced Ca^2+^ transients in MUT rats could also play a role in the failure of LTD induction. Similar threshold mechanisms for Ca^2+^ have been widely reported in hippocampus and neocortex, where increased Ca^2+^ influx leads to LTP and reduced Ca^2+^ levels induce LTD. Interestingly, haploinsufficiency in CAMTA1, a member of the calmodulin-binding transcription activators, results in early-onset ataxia in humans [79–81], and nervous system deletion of CAMTA1 in mice causes severe ataxia with Purkinje cell degeneration and cerebellar atrophy [82].

We focused our studies on LTP of PF, as previously it was reported that blockade of PF LTD by T-588, a calcium release blocker [83], did not impair motor learning on rotarod [84]. Repetitive stimulation of PFs at low frequency similar to the stimuli used in this study evokes postsynaptic LTP at PF-Purkinje cell synapses by increasing postsynaptic AMPA receptor numbers via inhibition of AMPA receptor-PICK1 interaction to potentiate the postsynaptic response [85]. Induction of postsynaptic LTP at PF-Purkinje cell synapses requires low levels of postsynaptic Ca^2+^and protein phosphatase activation [86, 87]. However, it was reported that Purkinje cell-specific knockout of the protein phosphatase PP2B impairs potentiation and cerebellar motor learning, but induction of PF LTD was not affected [87]. It is suggested that motor learning results from the interaction of both LTP and LTD, and they originate from different cellular layers [88]. We see in our studies that the MUT rats show motor deficits and absence of LTP or LTD along with smaller evoked responses.

Further studies are needed to address effect of ELOVL4 mutations on AMPA receptor trafficking in Purkinje cells, and other forms of cerebellar synaptic plasticity as pure presynaptic LTP of PF [89] or mGLuR1-dependent PF plasticity of long-latency patches organized in parasagittal bands [90]. Remarkably, a mouse model of SCA type 2 caused by polyglutamine expansion in ataxin-2 had deficiency of C24/26-sphingomyelins contrasted with excess C18/20-sphingomyelin [91]. These changes in sphingomyelins were caused mostly by decreased levels of Elovl4 expression in the cerebellum [91]. This suggests that impaired VLC-SFA synthesis may be a significant factor in other forms of SCAs, even when there is no ELOVL4 mutation detected.

In summary, we have generated a rat model of SCA34 that recapitulates the phenotype found in humans. The animals develop ataxia and show impaired synaptic function in CF and PF inputs into Purkinje cells. In turn, the impaired synaptic plasticity at these two major excitatory inputs to Purkinje cells degrades the operation of cerebellar cortex as an effective forward controller of movements [88]. These synaptic functional changes occur in the absence of overt anatomical changes or of detectable changes in major synaptic protein expression levels in the cerebellum and prior to neurodegeneration, suggesting that the phenotype originates as a synaptic plasticity deficit that likely also causes ataxia as observed in SCA34 patients.

## Conclusion

Different ELOVL4 mutations cause different tissue-specific disorders in humans. Here we report the first animal model of SCA34 caused by W246G ELOVL4 mutation that led to age-related impaired synaptic and motor function, prior to the onset of overt neurodegeneration and cerebellar atrophy. Our results suggest that in SCA34 patients, neuronal deficits likely arise from the impaired ability of W246G ELOVL4 to synthesize VLC-SFA, which we have previously shown to be enriched in synaptic vesicles and are necessary for modulating presynaptic release kinetics. Taken together, our findings suggest that onset of ataxia in SCA34 is associated with a synaptopathy present from early ages that over time leads to cerebellar degeneration and the severe motor impairments that characterize the disease. These findings support the essential role of ELOVL4 and its VLC-FA products in physiological neuronal function.

## Supplementary Information

Supplemental Fig. 1. Generation and characterization of c.736 T>G, p.W246G knock-in Long Evans rat using the CRISPR/Cas9 system. **a.** Schematic depiction of targeting strategy. The genomic region of rat *Elovl4* locus is diagrammed (gene is oriented from left to right; total size is 28.21 kb). Solid bars represent open reading frame (exons); open bars represent untranslated regions. The sgRNA cut site and the single strand oligonucleotide donor sequencing with homology arms and the mutation site is shown by the red arrow. **b.** Genotyping of WT, HET, and MUT SCA34-KI rats by StyI restriction analysis. 704 bp PCR products were generated from WT, HET, and MUT rats using PCR primers. The amplicons were run undigested or purified and digested with StyI restriction enzyme. Left panel: Undigested amplicons ran at 704 bp for WT, HET, and MUT rats, as expected. Right panel: Digestion with StyI restriction enzyme showed the WT PCR product is resistant to Sty I digestion. In contrast, HET rats show two bands, one corresponding to the WT PCR product and a smaller, digested fragment arising from the mutant PCR product containing the StyI digestion site. MUT rats show only a single band corresponding to the digested, mutant PCR product with no WT PCR product, as expected. (From: Agbaga et al., 2020[34]). (PNG 1092 kb)

High resolution image (TIF 1408 kb)

Supplemental Fig. 2. Sanger sequencing confirms appropriate gene editing. Sequencing from the 5’-3 primer direction (left to right on the figure), Sanger DNA sequencing of WT, HET, and MUT rat DNA sequences confirms the single point mutation c.736 T>G in the rat *Elovl4* genome. Box and arrows indicate site of gene editing. **a.** MUT. **b** HET. **c**. WT. (From: Agbaga et al., 2020[34]). (PNG 1558 kb)

High resolution image (TIF 5705 kb)

Supplemental Fig. 3. Whole genome sequence analysis. Reading from the 3’-5 primer direction (right to left on the figure), whole genome sequencing of WT and MUT rats confirms knockin of the 736 T>G, p.W246G mutant *Elovl4* without any major off target effects in MUT rats (MUT). The box highlights the position of the 736 T>G mutation. Each gray bar represents a NextGen sequence. Colored bases differ from the WT sequence. Bases matching the WT are shown in gray to highlight only mutant bases. Examples of whole genome sequencing from two WT (WT 1 and WT2) and two MUT (MUT1 and MUT2) rats are shown. (From: Agbaga et al., 2020[34]). (PNG 385 kb)

High resolution image (TIF 1852 kb)

Supplemental Fig. 4. Gross physical phenotype of SCA34-KI rats. **a**. Gross appearance of WT and HET SCA34-KI rats is similar. MUT SCA34-KI rats show hair loss and erythrokeratodermia variabilis (EKV). **b.** Comparison of the underside of WT and MUT SCA34-KI rats showing marked hair loss and EKV. **c.** Hair loss around the eyes nose and ears (arrows) on a MUT SCA34-KI rat. **d.** WT rat pups show normal eyelids with full opening (P45 shown). **e.** MUT SCA34-KI rats show stiff, swollen eyelids at early ages that open incompletely (P45 shown), which resolves by about P60. Hair loss around the eye is also common in MUT rats. **f.** Adult MUT rats show complete opening of the lids. (From: Agbaga et al., 2020[34]). (PNG 3491 kb)

High resolution image (TIF 4144 kb)

Supplemental Fig. 5. The W246G mutation in ELOVL4 impairs VLC-SFA synthesis. **a.** Analysis of VLC-SFA in skin. Levels of VLC-SFA (28:0 and 30:0) and total VLC-SFA (28:0+30:0) were significantly reduced in the skin of MUT rats compared with WT and HET rats. **b.** Levels of 26:0, the direct precursor for VLC-SFA synthesis, did not differ significantly across genotypes. However, levels of 24:0 were significantly elevated in the skin of HET and MUT rats compared with WT rats. (Data are shown as mean +/- St. Dev. Analysis by 1-way ANOVA with Tukey's post-hoc test. *, p<0.05; **, p<0.01: ***, p<0.001). (From: Agbaga et al., 2020[34]). (PNG 64 kb)

High resolution image (TIF 255 kb)

Supplemental Fig. 6. W246G ELOVL4 retains the ability to synthesize VLC-PUFA. Analysis of retinal glycerophospholipids from WT, HET, and MUT SCA34-KI rats shows that the W246G mutant form of ELOVL4 retains the capacity to synthesize VLC-PUFA at normal levels. No differences in total retinal levels of VLC-PUFA were present among WT, HET, and MUT rats. **a:** VLC-PUFA were detected specifically in the phosphatidylcholine fraction (PC), but total VLC-PUFA levels showed no differences among WT, HET, and MUT rat retina. However, significant differences were detected in non-VLC-FA (PC 34:01 and PC 40:06) among genotypes. **b:** No VLC-PUFA were detected in the phosphatidylethanolamine (PE) fraction of WT, HET, or MUT rat retina. However, statistically significant differences were detected in PE 40:06 and PC 44:12. **c:** No VLC-PUFA were detected in the phosphatidylserine (PS) fraction of WT, HET, or MUT rat retina. No significant differences were detected in any lipid species in the PS fraction. (Analysis by 1-way ANOVA with Tukey's post-hoc test. Data are shown as mean +/- St. Dev. *, MUT differs from WT; $, HET differs from WT; #, MUT differs from WT). (From: Agbaga et al., 2020[34]). (PNG 123 kb)

High resolution image (TIF 527 kb)

Supplemental Fig. 7. Male WT, HET, and MUT rats all show poor performance on the rotarod test (distance traveled variable shown). (***A***) Rotarod performance of male WT, HET, and MUT rats at 2 months of age. (***B***) Rotarod performance of WT, HET, and MUT male rats at 6 months of age. (n= 5 WT, 7 HET, 4 MUT). Analysis by 1-way ANOVA with Tukey’s post-hoc test showed no significant difference across genotypes. (PNG 114 kb)

High resolution image (TIF 247 kb)

Supplemental Fig. 8. Purkinje cell organization in WT, HET, and MUT rat cerebellum is comparable from P45 to 6 months. Immunolabeling for calbindin shows that Purkinje cells form a monolayer in the Purkinje cell layer (PCL), as appropriate, and have comparable organization across age-matched WT, HET, and MUT rats. (**A-C**) Postnatal day 45 (P45). (***D-F***) 2 months of age. (***G-I***) 6 months of age. Panels G-I of this figure match panels A-C shown in Figure 5. ML, molecular layer; GCL, granule cell layer. Scale bars = 200 μm for each row. (PNG 1872 kb)

High resolution image (TIF 2787 kb)

Supplemental Fig. 9. Granule cells in WT, HET, and MUT rat cerebellum are comparable from P45 to 6 months. Immunolabeling for NeuN, a granule cell marker, shows that granule cells are distributed appropriately in the granule cell layer (GCL) in WT, HET, and MUT rat cerebellum from P45 to 6 months of age. A small population of NeuN-positive cells is also present in the molecular layer (ML), as appropriate. (***A-B***) P45. (***D-F***) 2 months. (***G-I***) 6 months. Panels G-I of this figure match panels G-I shown in Figure 5. PCL, Purkinje cell layer. Scale bars = 200 μm for each row. (PNG 1441 kb)

High resolution image (TIF 1941 kb)

Supplemental Fig. 10. Immunolabeling for Vesicular Glutamate Transporter 1 (VGluT1) in WT, HET, and MUT rat cerebellum is comparable from P45 to 6 months of age. Immunolabeling for VGluT1 shows that mossy fiber inputs project to the glomeruli of the granule cell layer (GCL) and that PFs are distributed throughout the entire molecular layer (ML) of WT, HET, and MUT cerebellum from P45 to 6 months of age, as appropriate. (***A-C***) P45. (***D-F***) 2 months. (***G-I***) 6 months. Panels G-I of this figure match panels J-L shown in Figure 5. PCL, Purkinje cell layer. Scale bars = 200 μm for each row. (PNG 1781 kb)

High resolution image (TIF 2514 kb)

Supplemental Fig. 11. Immunolabeling for synaptotagmin 2 (Syt2) in WT, HET, and MUT rat cerebellum is comparable from P45 to 6 months of age. Syt2 is present in the glomeruli of the granule cell layer (GCL) and throughout the molecular layer (ML), as appropriate. (***A-C***) P45. (***D-F***) 2 months. (***G-I***) 6 months. Panels G-I of this figure match panels M-O shown in Figure 5. PCL, Purkinje cell layer. Scale bars = 200 μm for each row. (PNG 2581 kb)

High resolution image (TIF 3672 kb)

Supplemental Fig. 12. (***A-C***) Distribution of Protein Kinase Cɣ (PKCɣ) immunolabeling is comparable in WT (A), HET (B), and MUT (C) cerebellum. Scale bar = 100 μm for all panels. (***D***) Western blotting revealed no significant difference in PKG levels in WT, HET, and MUT cerebellum. (PNG 997 kb)

High resolution image (JPG 925 kb)

Supplemental Fig. 13. (***A-C***) Distribution of Inositol Phosphate 3 Receptor (IP3R) immunolabeling is comparable in WT (A), HET (B), and MUT (C) cerebellum. Scale bar = 100 μm for all panels. (***D***) Western blotting revealed no significant difference in IP3R levels in WT, HET, and MUT cerebellum. (PNG 933 kb)

High resolution image (JPG 845 kb)

Supplemental Fig. 14. Immunolabeling for Glial Fibrillary Acidic Protein (GFAP) in WT, HET, and MUT rat cerebellum is comparable from P45 to 6 months of age. Immunolabeling for GFAP shows that astrocytes are distributed appropriately, with no evidence of gliosis, in the WT, HET, and MUT rat cerebellum. (***A-C***) P45. (***D-F***) 2 months. (***G-I***) 6 months. ML, molecular layer; PCL, Purkinje cell layer; GCL, granule cell layer; bv, blood vessel. Scale bars = 200 μm for each row. (PNG 1572 kb)

High resolution image (TIF 2274 kb)

Supplemental Fig. 15. Microglia show “quiescent” ramified morphology in WT, HET, and MUT rat cerebellum. Tomato lectin (TL) labels blood vessels (bv) and microglial cells (arrowheads). Microglia show ramified morphology with symmetrically distributed processes typical of quiescent glia performing surveillance functions in the cerebellum of WT, HET, and MUT rats. (***A-C***) WT. (***D-F***) HET. (***G-I***) MUT. Molecular layer (ML). All images shown at 6 months of age. Scale bar = 200 μm for all panels. (PNG 1519 kb)

High resolution image (TIF 1690 kb)

Supplemental Fig. 16. Impaired synaptic plasticity in both male and female mutant rats. (***A***) LTP induced by stimulation of the PFs in the molecular layer at 1 Hz for 10 min induces LTP in female WT rat cerebellar slices (*upper panel,* green circles, ♀WT = 155.17 ± 17.38% normalized slope, n = 6 slices from 3 rats), but not in cerebellar slices from female MUT rats (*upper panel,* red triangles, ♀MUT = 97.87 ± 7.42% normalized slope, n = 8 slices from 3 rats). Similarly, the stimulation of the PFs at 1 Hz induces LTP in male WT rat cerebellar slices (*lower panel,* green circles, ♂WT = 140.45 ± 8.18% normalized slope, n = 8 slices from 3 rats), but not in cerebellar slices from male MUT rats (*lower panel,* red triangles, ♂MUT = 93.12 ± 5.94% normalized slope, n = 8 slices from 3 rats). (***B***) Stimulating the CFs in the granule cell layer at 1 Hz for 10 min induces LTD in female WT cerebellar slices (*upper panel,* green circles, ♀WT = 59.72 ± 6.66% normalized slope, n = 6 slices from 3 rats), but not in cerebellar slices from female MUT rats (*upper panel,* red triangles, ♀MUT = 92.13 ± 4.54% normalized slope, n = 8 slices from 3 rats). Similarly, the stimulation of the CFs at 1 Hz induces LTD in male WT rat cerebellar slices (*lower panel,* green circles, ♂WT = 63.13 ± 4.85% normalized slope, n = 4 slices from 2 rats), but not in cerebellar slices from male MUT rats (*lower panel,* red triangles, ♂MUT = 92.12 ± 8.14% normalized slope, n = 6 slices from 3 rats). Each data point represents the average of two successive test responses. The vertical gray bar indicates the period of 1 Hz stimulation. Data are shown as mean ± SEM. * p < 0.05, ** p < 0.01, ***p < 0.001, **** p < 0.0001, two-way repeated measures ANOVA with Bonferroni post-hoc test. (PNG 110 kb)

High resolution image (TIF 174 kb)

Supplemental Fig. 17. Synaptic plasticity responses do not differ in female and male rats. (***A***) LTP induced by stimulation of the PFs in the molecular layer at 1 Hz for 10 min does not induce LTP in female MUT rats (*upper panel,* red triangles, ♀MUT = 97.87 ± 7.42% normalized slope, n = 8 slices from 3 rats) nor in cerebellar slices from male MUT rats (*upper panel,* red and black triangles, ♂MUT = 93.12 ± 5.94% normalized slope, n = 8 slices from 3 rats). The stimulation of the PFs at 1 Hz induces LTP in female WT rat cerebellar slices (*lower panel,* green circles, ♀WT = 155.17 ± 17.38% normalized slope, n = 6 slices from 3 rats), and also in male WT rat cerebellar slices (*lower panel,* green and black circles, ♂WT = 140.45 ± 8.18% normalized slope, n = 8 slices from 3 rats). (***B***) Stimulating the CFs in the granule cell layer at 1 Hz for 10 min does not induce LTD in cerebellar slices from female MUT rats (*upper panel,* red triangles, ♀MUT = 92.13 ± 4.54% normalized slope, n = 8 slices from 3 rats) nor in cerebellar slices from male MUT rats (*upper panel,* red and black triangles, ♂MUT = 92.12 ± 8.14% normalized slope, n = 6 slices from 3 rats). The stimulation of the CFs at 1 Hz induces LTD in female WT cerebellar slices (*lower panel,* green circles, ♀WT = 59.72 ± 6.66% normalized slope, n = 6 slices from 3 rats), and also in male WT rat cerebellar slices (*lower panel,* green and black circles, ♂WT = 63.13 ± 4.85% normalized slope, n = 4 slices from 2 rats). Each data point represents the average of two successive test responses. The vertical gray bar indicates the period of 1 Hz stimulation. Data are shown as mean ± SEM. p>0.05 for all panels, two-way repeated measures ANOVA with Bonferroni post-hoc test. (PNG 99 kb)

High resolution image (TIF 162 kb)

## Data Availability

All data generated or analyzed during this study are included in this published article (and its supplementary information files). Any other information needed on the datasets used and/or analyzed during the current study is available from the corresponding author on reasonable request.

## Abbreviations

AMPA: α-amino-3-hydroxy-5-methyl-4-isoxazolepropionic acid
CF: climbing fibers
EKV: erythrokeratodermia variabilis
ELOVL4: fatty acid elongase-4 or elongation of very long chain fatty acids-4
fEPSP: field excitatory postsynaptic potential
GABA: γ-aminobutyric acid
GAD65/67: glutamate decarboxylase-65/67
GFAP: glial fibrillary acidic protein
IP3: inositol-(1,4,5)-tris phosphate
KI: knock-in
LTD: long-term depression
LTP: long-term potentiation
MEA: multi-electrode array
mGluR1: metabotropic glutamate receptor subtype 1
PF: parallel fibers
PSD-95: postsynaptic density protein-95
SCA34: autosomal dominant spinocerebellar ataxia-34
STGD3: autosomal dominant Stargardt-like macular dystrophy
SYT2: synaptotagmin 2
VGluT1/2: vesicular glutamate transporter (VGLUT)/(VGLUT2)
VLC-FA: very long chain fatty acids
VLC-PUFA: very long chain polyunsaturated fatty acids
VLC-SFA: very long chain saturated fatty acids
