# Supplementary figures and images for: W246G Mutant ELOVL4 Impairs Synaptic Plasticity in Parallel and Climbing Fibers and Causes Motor Defects in a Rat Model of SCA34
Source: Mol Neurobiol. 2021 Jul 5;58(10):4921–43. doi: 10.1007/s12035-021-02439-1 (PMC8497303; doi:10.1007/s12035-021-02439-1)

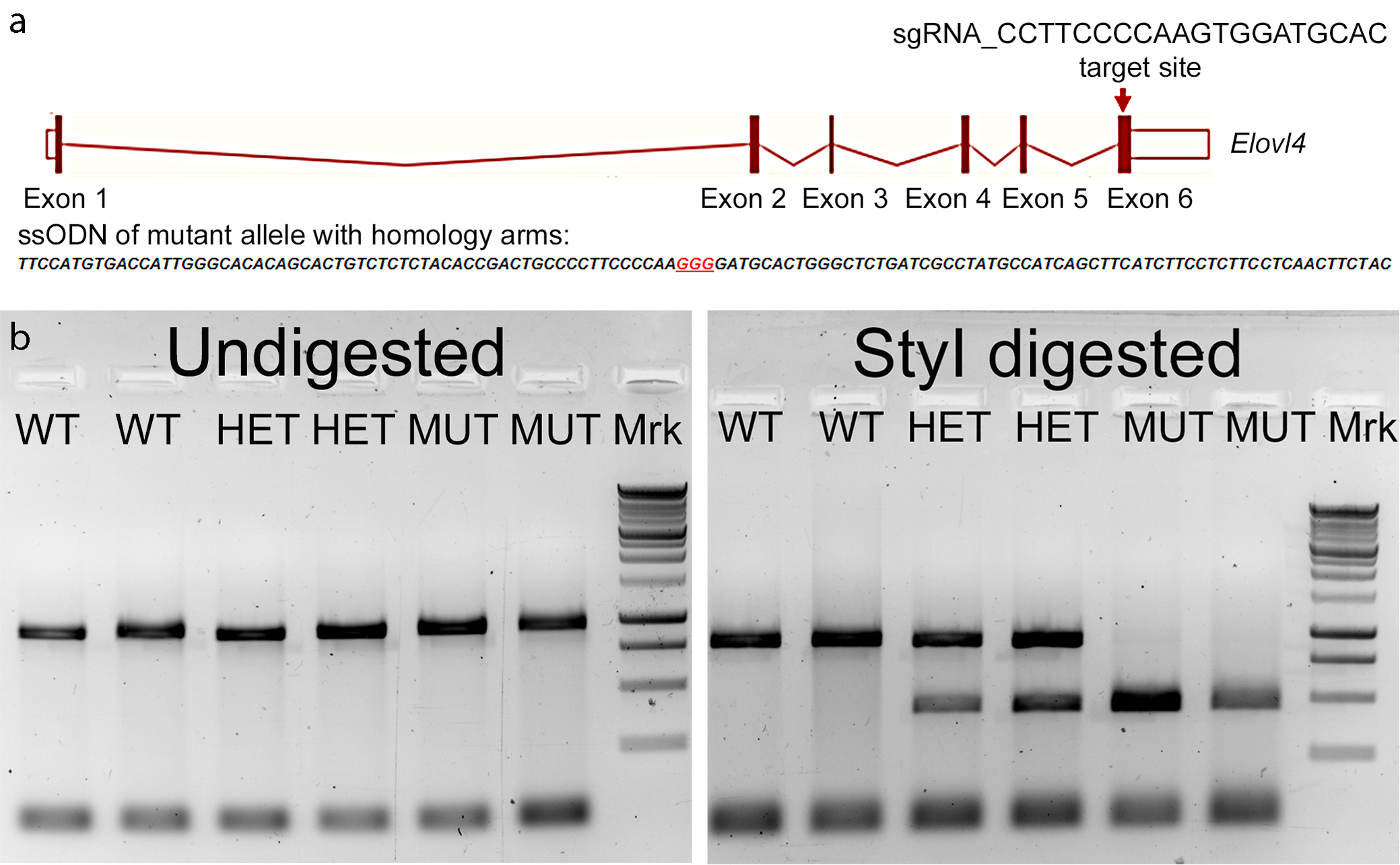

Supplement: Supplementary file 1 — Generation and characterization of c.736 T>G, p.W246G knock-in Long Evans rat using the CRISPR/Cas9 system. a. Schematic depiction of targeting strategy. The genomic region of rat Elovl4 locus is diagrammed (gene is oriented from left to right; total size is 28.21 kb). Solid bars represent open reading frame (exons); open bars represent untranslated regions. The sgRNA cut site and the single strand oligonucleotide donor sequencing with homology arms and the mutation site is shown by the red arrow. b. Genotyping of WT, HET, and MUT SCA34-KI rats by StyI restriction analysis. 704 bp PCR products were generated from WT, HET, and MUT rats using PCR primers. The amplicons were run undigested or purified and digested with StyI restriction enzyme. Left panel: Undigested amplicons ran at 704 bp for WT, HET, and MUT rats, as expected. Right panel: Digestion with StyI restriction enzyme showed the WT PCR product is resistant to Sty I digestion. In contrast, HET rats show two bands, one corresponding to the WT PCR product and a smaller, digested fragment arising from the mutant PCR product containing the StyI digestion site. MUT rats show only a single band corresponding to the digested, mutant PCR product with no WT PCR product, as expected. (From: Agbaga et al., 2020[34]). (PNG 1092 kb) [file 12035_2021_2439_Fig10_ESM.png]

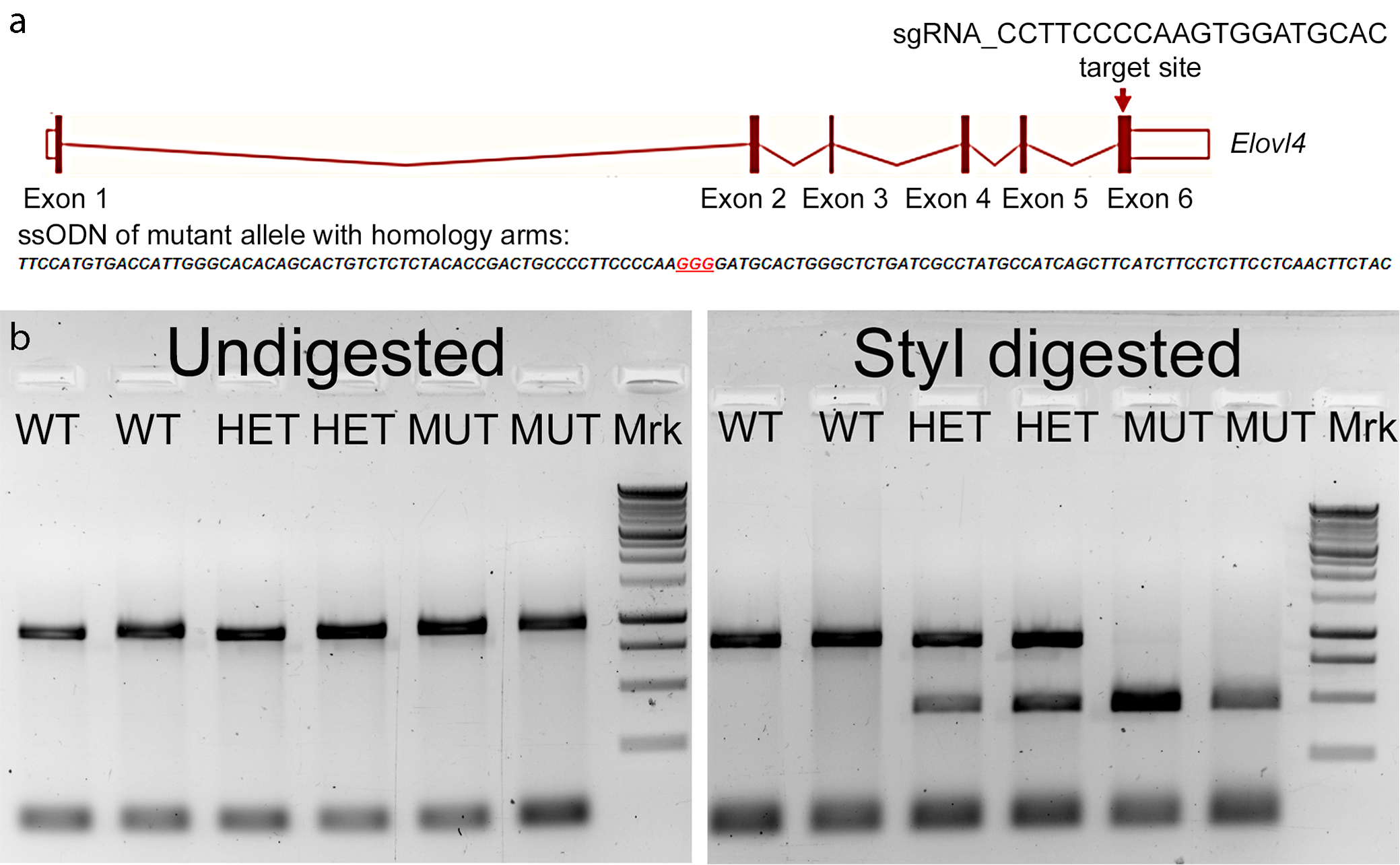

Supplement: Supplementary file 2 — High resolution image (TIF 1408 kb) [file 12035_2021_2439_MOESM1_ESM.tif]

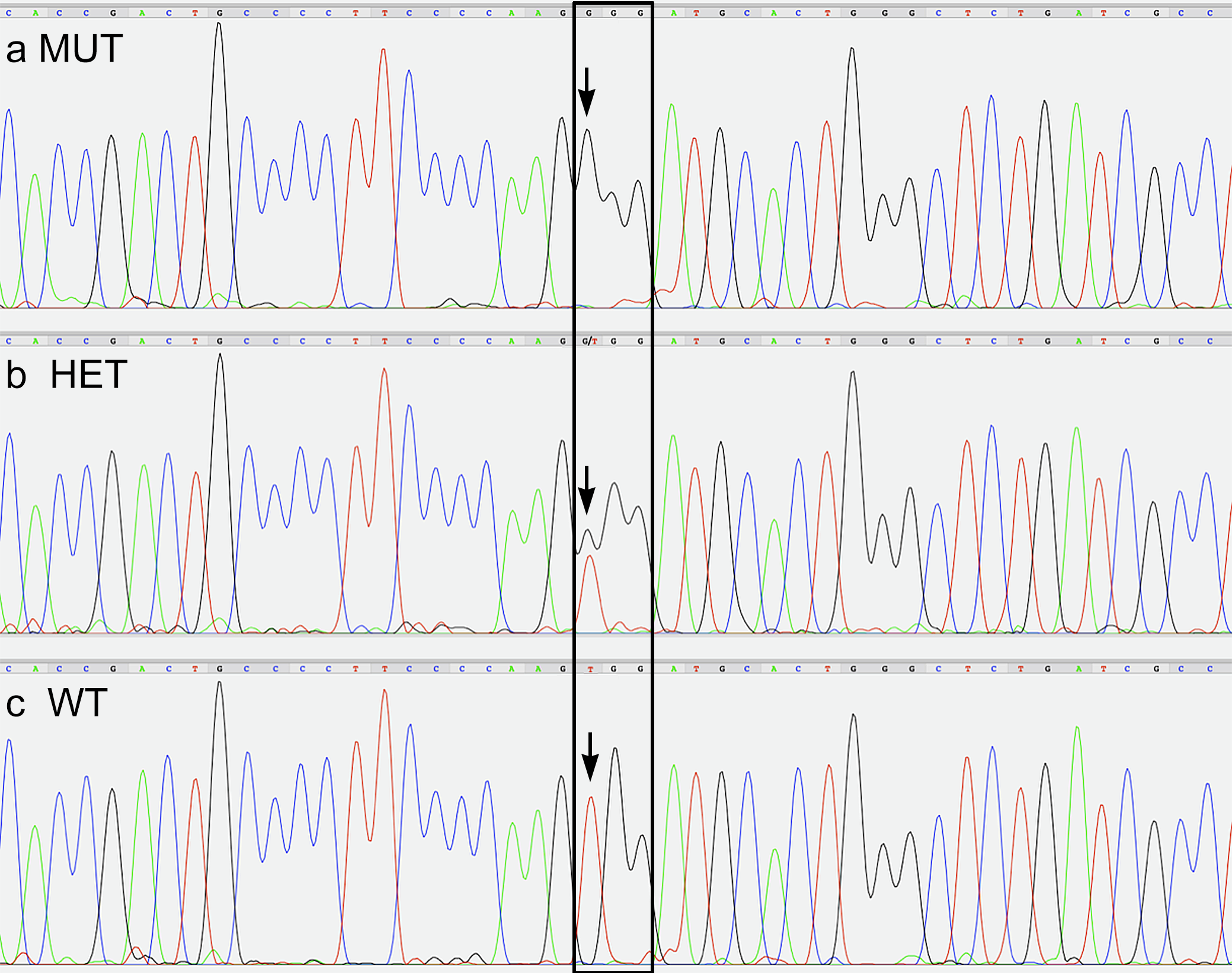

Supplement: Supplementary file 3 — Sanger sequencing confirms appropriate gene editing. Sequencing from the 5’-3 primer direction (left to right on the figure), Sanger DNA sequencing of WT, HET, and MUT rat DNA sequences confirms the single point mutation c.736 T>G in the rat Elovl4 genome. Box and arrows indicate site of gene editing. a. MUT. b HET. c. WT. (From: Agbaga et al., 2020[34]). (PNG 1558 kb) [file 12035_2021_2439_Fig11_ESM.png]

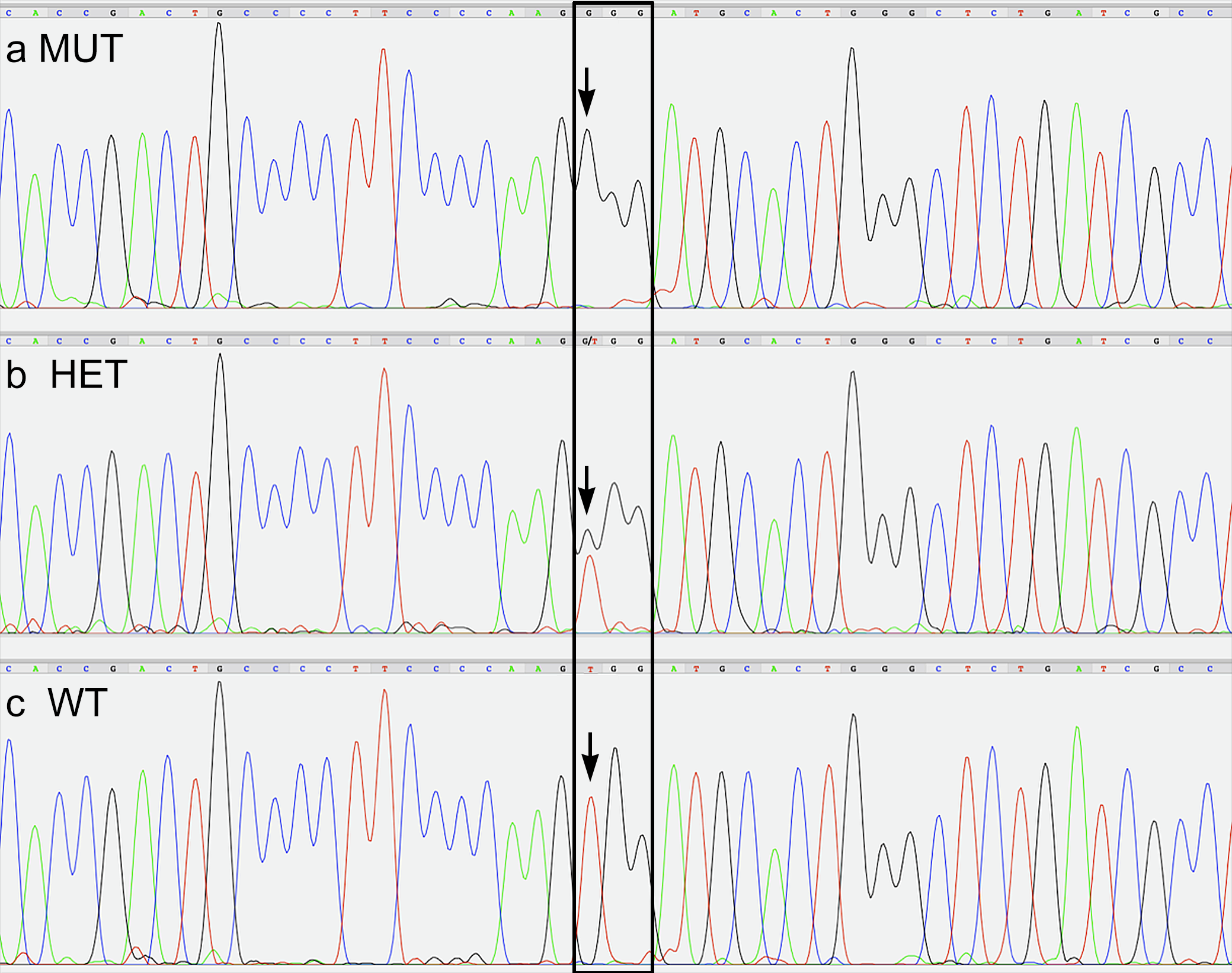

Supplement: Supplementary file 4 — High resolution image (TIF 5705 kb) [file 12035_2021_2439_MOESM2_ESM.tif]

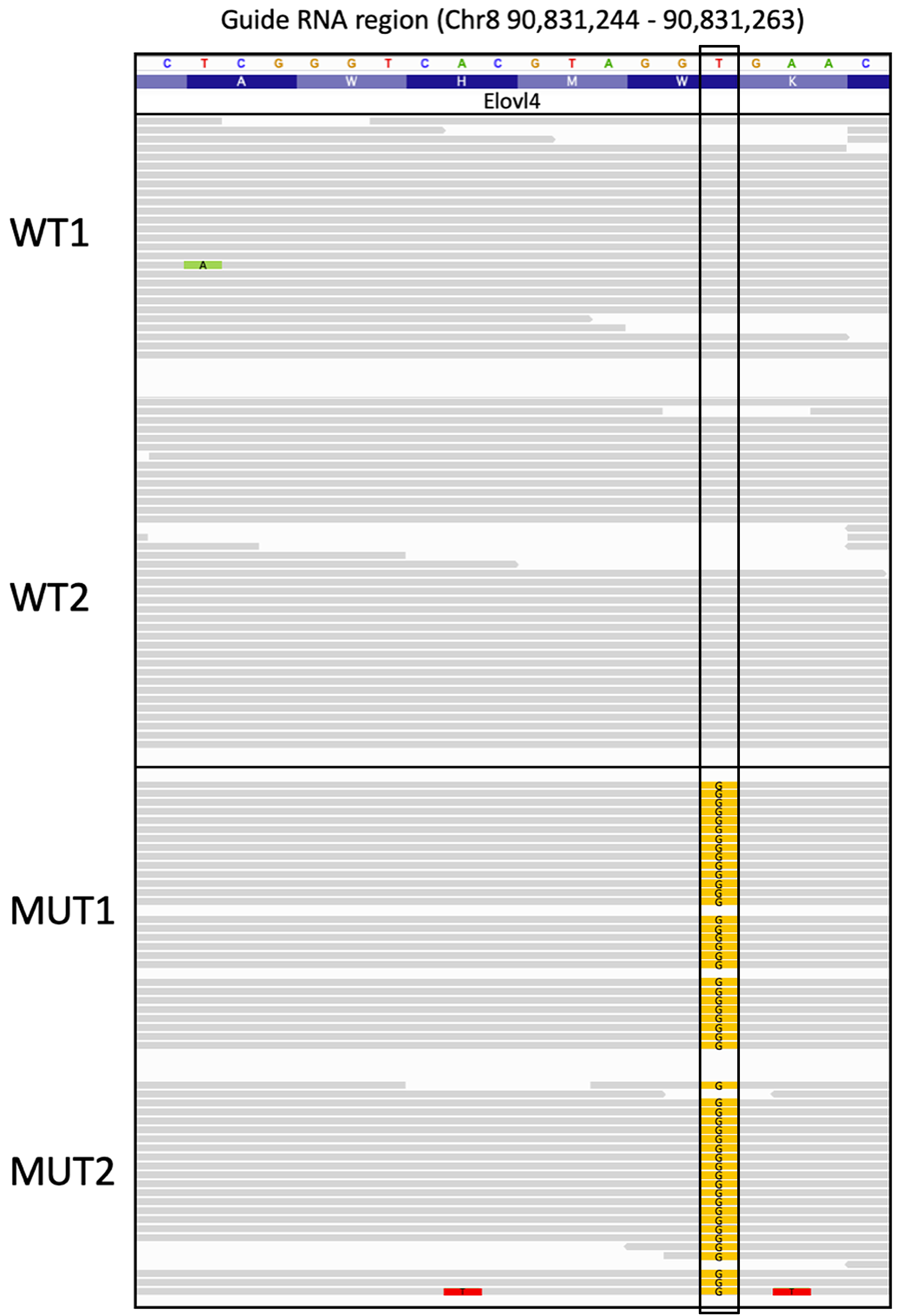

Supplement: Supplementary file 5 — Whole genome sequence analysis. Reading from the 3’-5 primer direction (right to left on the figure), whole genome sequencing of WT and MUT rats confirms knockin of the 736 T>G, p.W246G mutant Elovl4 without any major off target effects in MUT rats (MUT). The box highlights the position of the 736 T>G mutation. Each gray bar represents a NextGen sequence. Colored bases differ from the WT sequence. Bases matching the WT are shown in gray to highlight only mutant bases. Examples of whole genome sequencing from two WT (WT 1 and WT2) and two MUT (MUT1 and MUT2) rats are shown. (From: Agbaga et al., 2020[34]). (PNG 385 kb) [file 12035_2021_2439_Fig12_ESM.png]

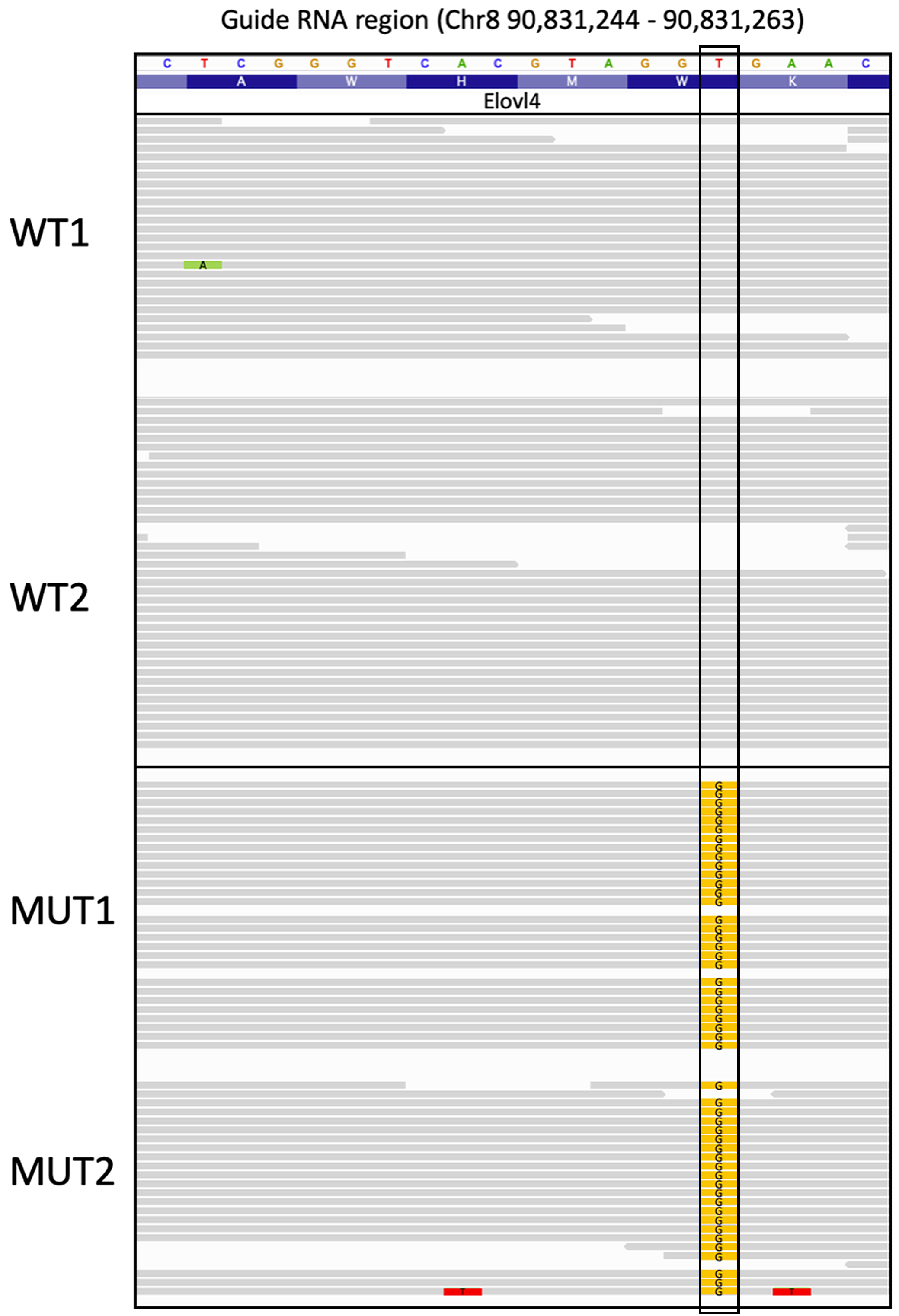

Supplement: Supplementary file 6 — High resolution image (TIF 1852 kb) [file 12035_2021_2439_MOESM3_ESM.tif]

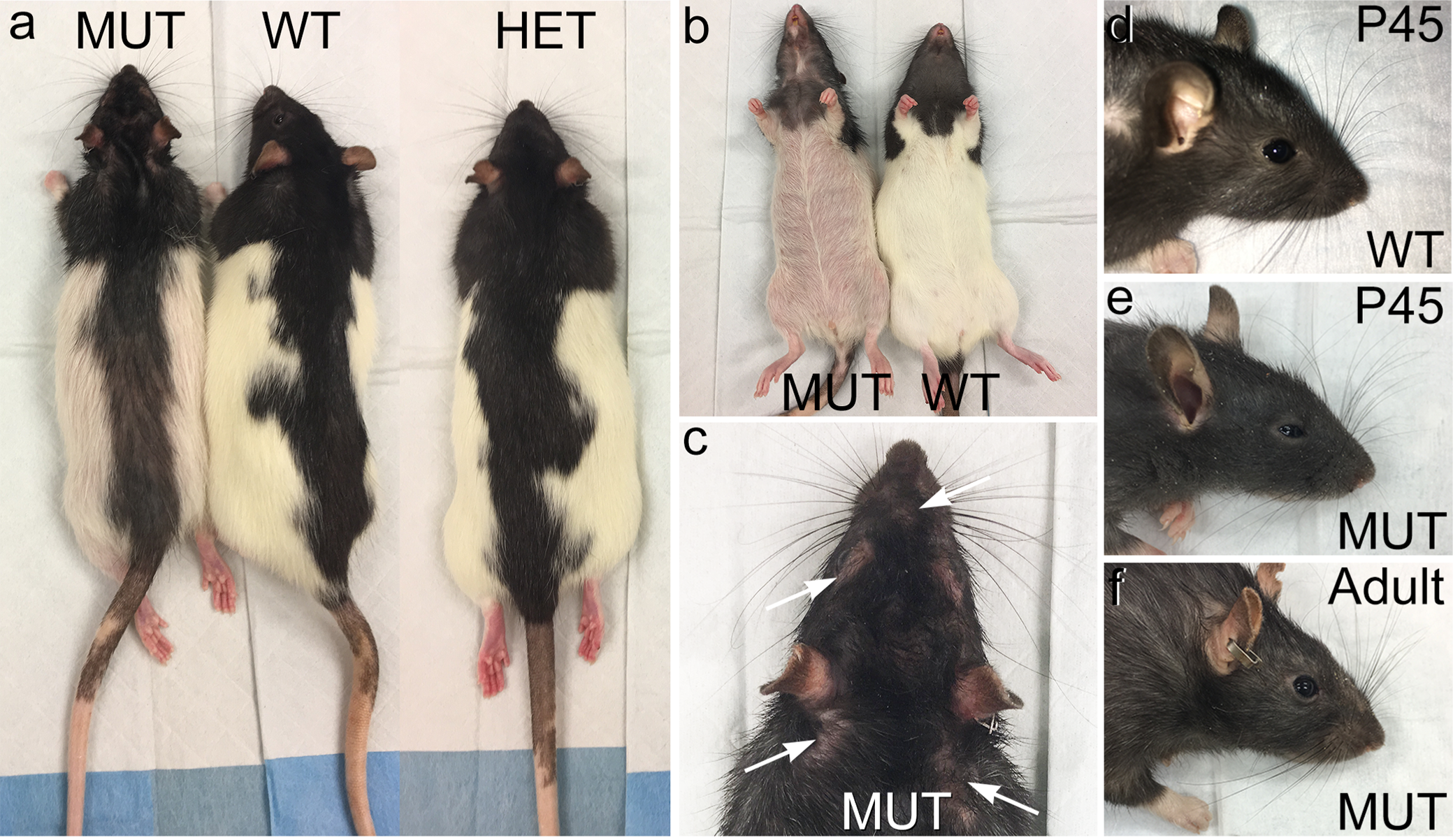

Supplement: Supplementary file 7 — Gross physical phenotype of SCA34-KI rats. a. Gross appearance of WT and HET SCA34-KI rats is similar. MUT SCA34-KI rats show hair loss and erythrokeratodermia variabilis (EKV). b. Comparison of the underside of WT and MUT SCA34-KI rats showing marked hair loss and EKV. c. Hair loss around the eyes nose and ears (arrows) on a MUT SCA34-KI rat. d. WT rat pups show normal eyelids with full opening (P45 shown). e. MUT SCA34-KI rats show stiff, swollen eyelids at early ages that open incompletely (P45 shown), which resolves by about P60. Hair loss around the eye is also common in MUT rats. f. Adult MUT rats show complete opening of the lids. (From: Agbaga et al., 2020[34]). (PNG 3491 kb) [file 12035_2021_2439_Fig13_ESM.png]

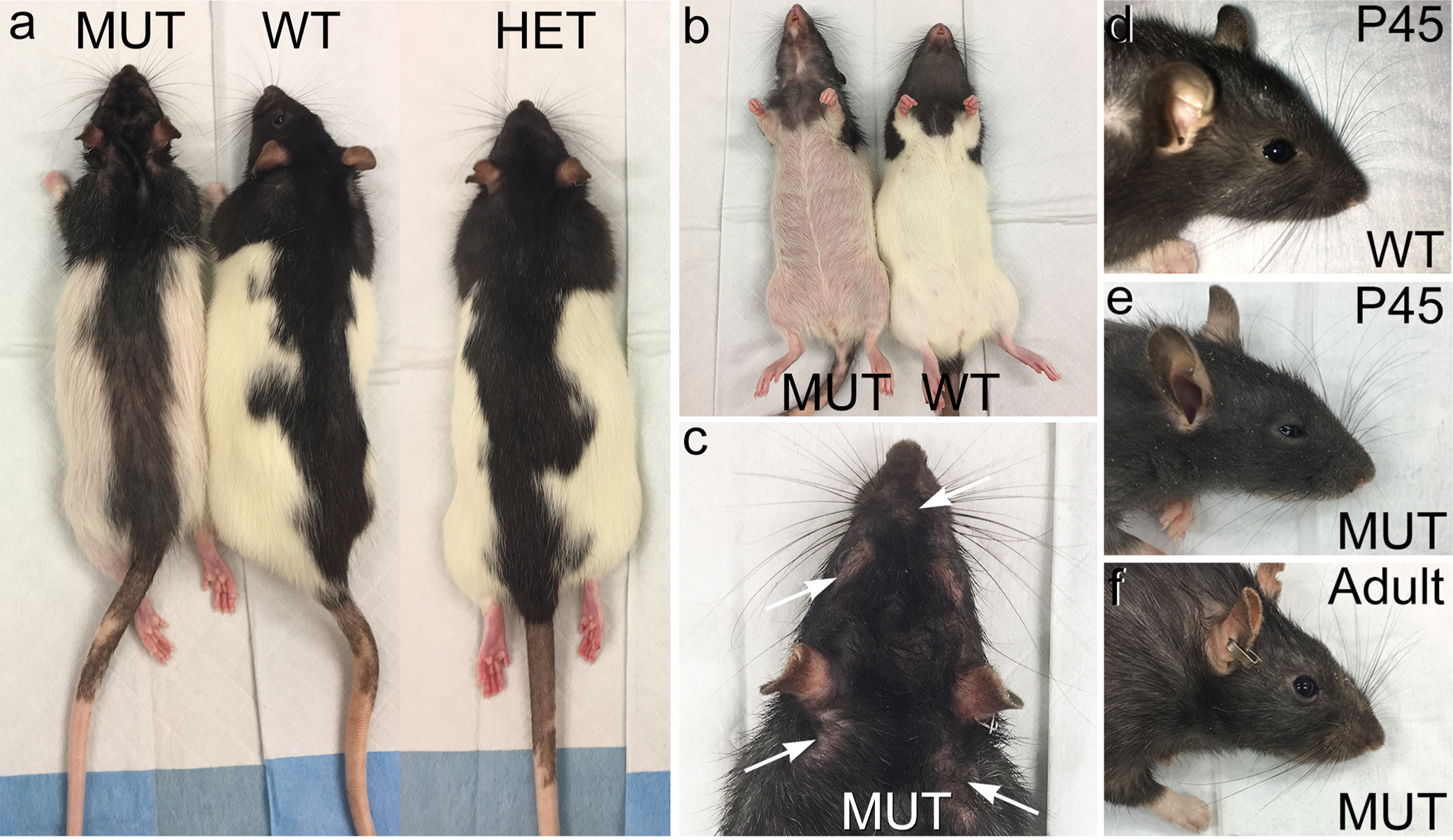

Supplement: Supplementary file 8 — High resolution image (TIF 4144 kb) [file 12035_2021_2439_MOESM4_ESM.tif]

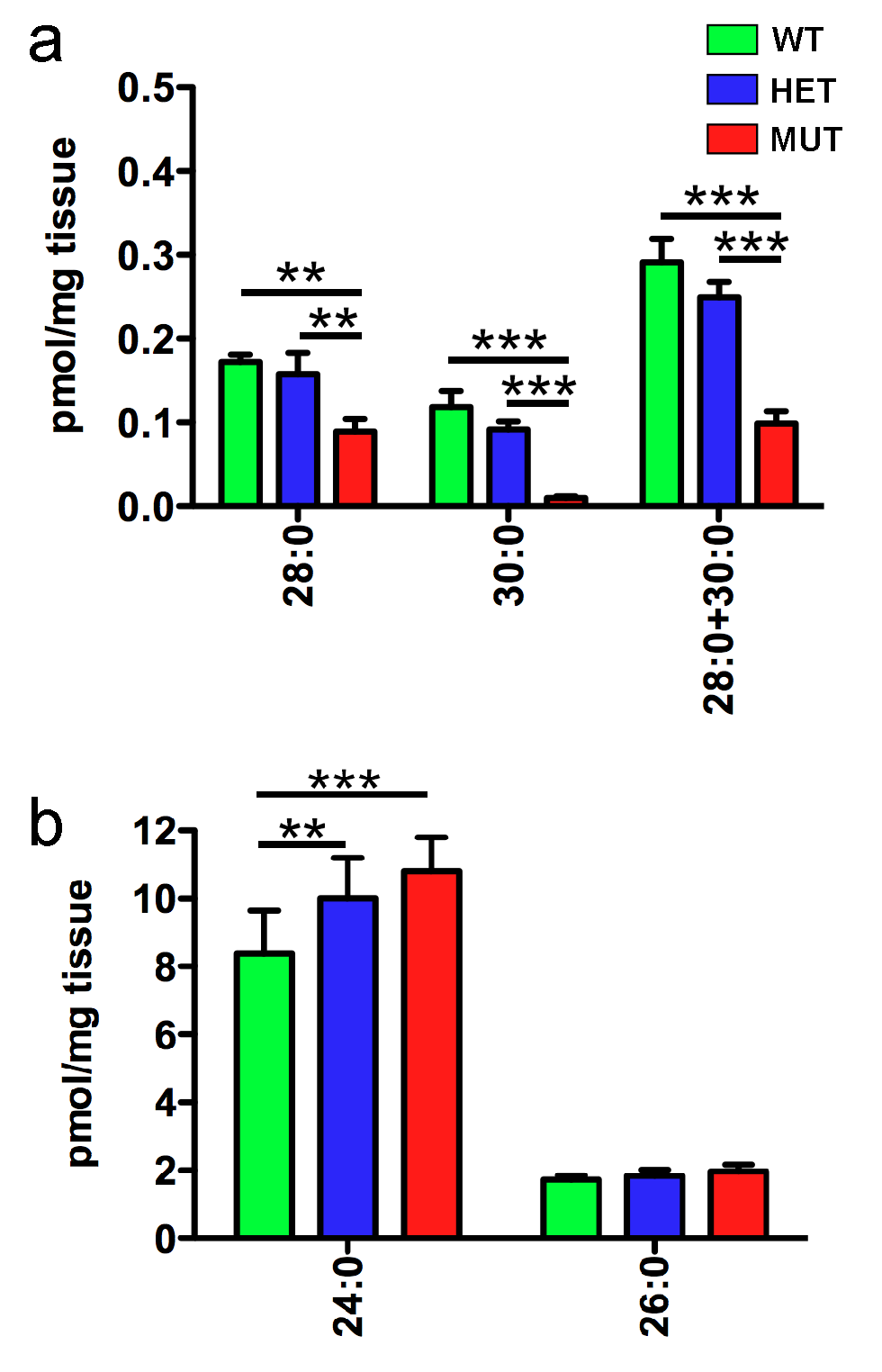

Supplement: Supplementary file 9 — The W246G mutation in ELOVL4 impairs VLC-SFA synthesis. a. Analysis of VLC-SFA in skin. Levels of VLC-SFA (28:0 and 30:0) and total VLC-SFA (28:0+30:0) were significantly reduced in the skin of MUT rats compared with WT and HET rats. b. Levels of 26:0, the direct precursor for VLC-SFA synthesis, did not differ significantly across genotypes. However, levels of 24:0 were significantly elevated in the skin of HET and MUT rats compared with WT rats. (Data are shown as mean +/- St. Dev. Analysis by 1-way ANOVA with Tukey's post-hoc test. *, p<0.05; **, p<0.01: ***, p<0.001). (From: Agbaga et al., 2020[34]). (PNG 64 kb) [file 12035_2021_2439_Fig14_ESM.png]

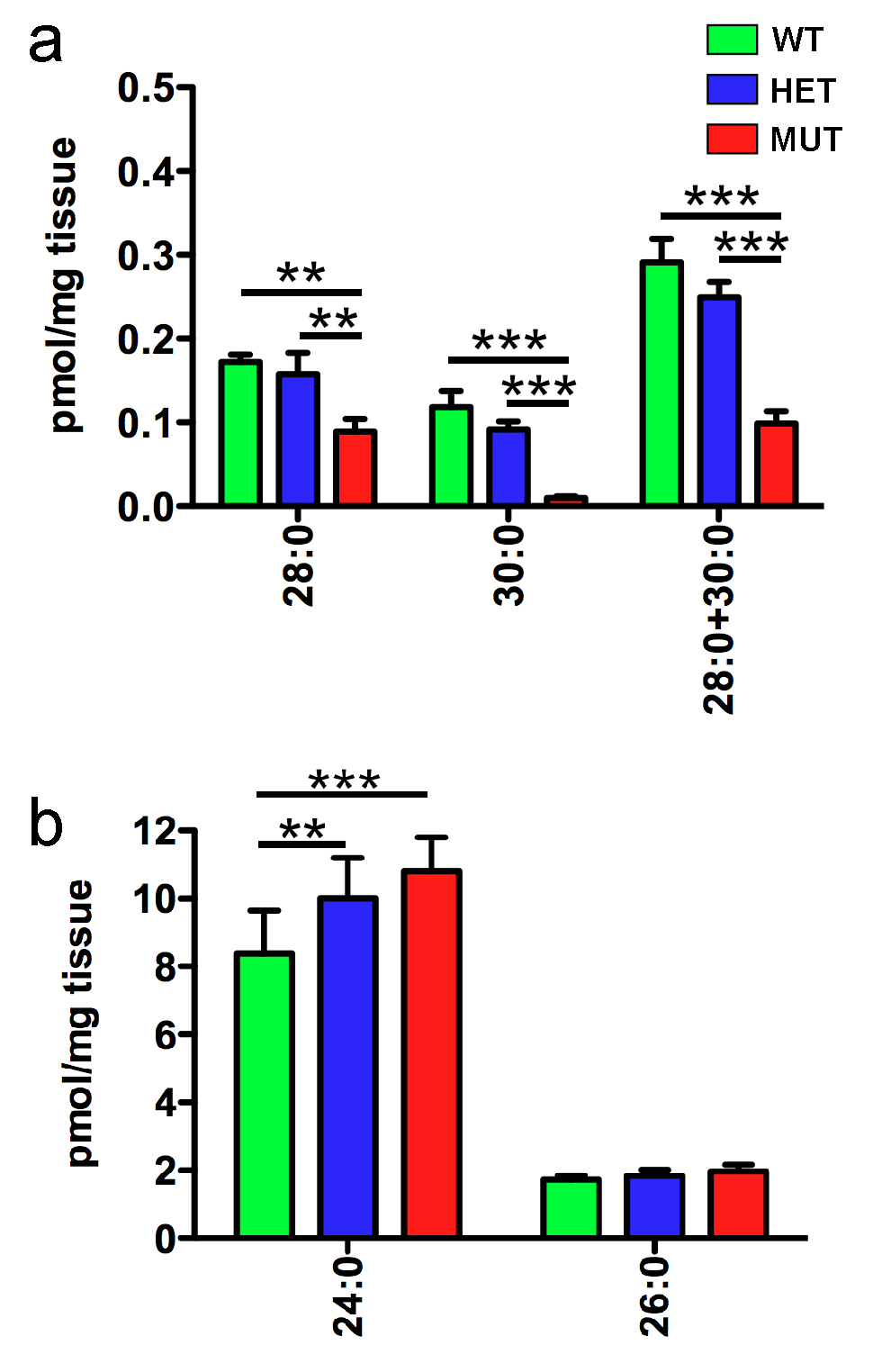

Supplement: Supplementary file 10 — High resolution image (TIF 255 kb) [file 12035_2021_2439_MOESM5_ESM.tif]

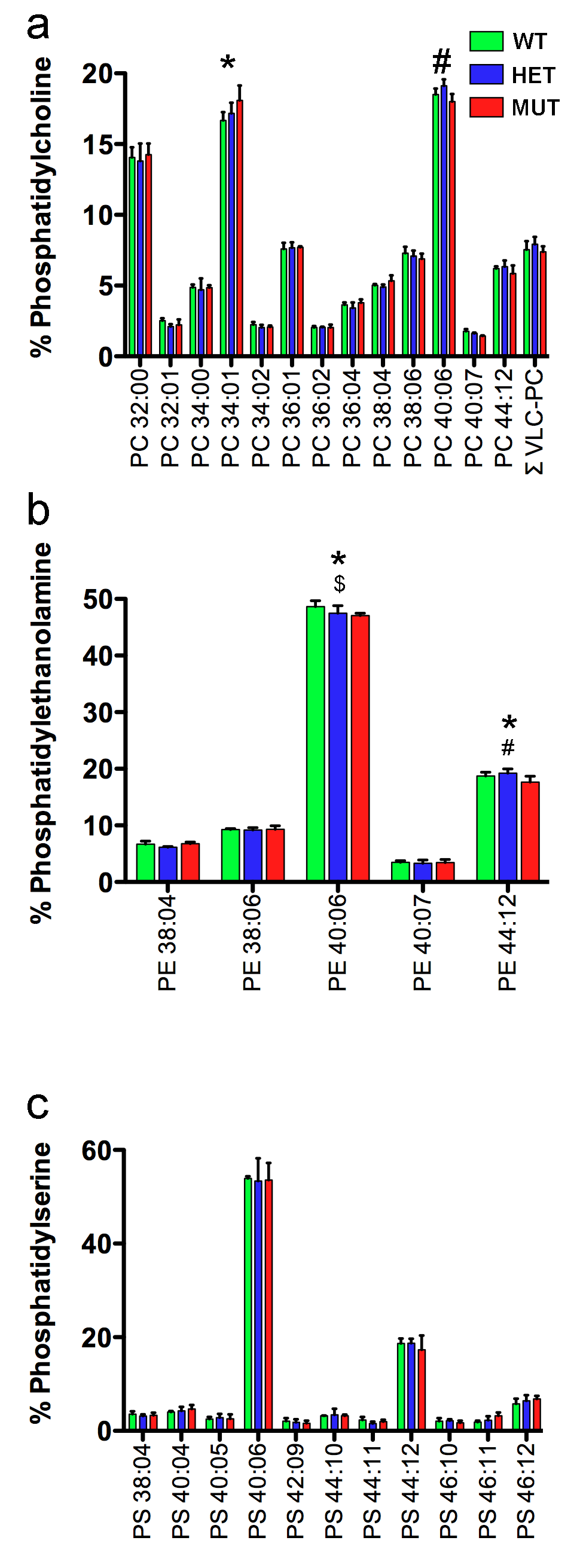

Supplement: Supplementary file 11 — W246G ELOVL4 retains the ability to synthesize VLC-PUFA. Analysis of retinal glycerophospholipids from WT, HET, and MUT SCA34-KI rats shows that the W246G mutant form of ELOVL4 retains the capacity to synthesize VLC-PUFA at normal levels. No differences in total retinal levels of VLC-PUFA were present among WT, HET, and MUT rats. a: VLC-PUFA were detected specifically in the phosphatidylcholine fraction (PC), but total VLC-PUFA levels showed no differences among WT, HET, and MUT rat retina. However, significant differences were detected in non-VLC-FA (PC 34:01 and PC 40:06) among genotypes. b: No VLC-PUFA were detected in the phosphatidylethanolamine (PE) fraction of WT, HET, or MUT rat retina. However, statistically significant differences were detected in PE 40:06 and PC 44:12. c: No VLC-PUFA were detected in the phosphatidylserine (PS) fraction of WT, HET, or MUT rat retina. No significant differences were detected in any lipid species in the PS fraction. (Analysis by 1-way ANOVA with Tukey's post-hoc test. Data are shown as mean +/- St. Dev. *, MUT differs from WT; $, HET differs from WT; #, MUT differs from WT). (From: Agbaga et al., 2020[34]). (PNG 123 kb) [file 12035_2021_2439_Fig15_ESM.png]

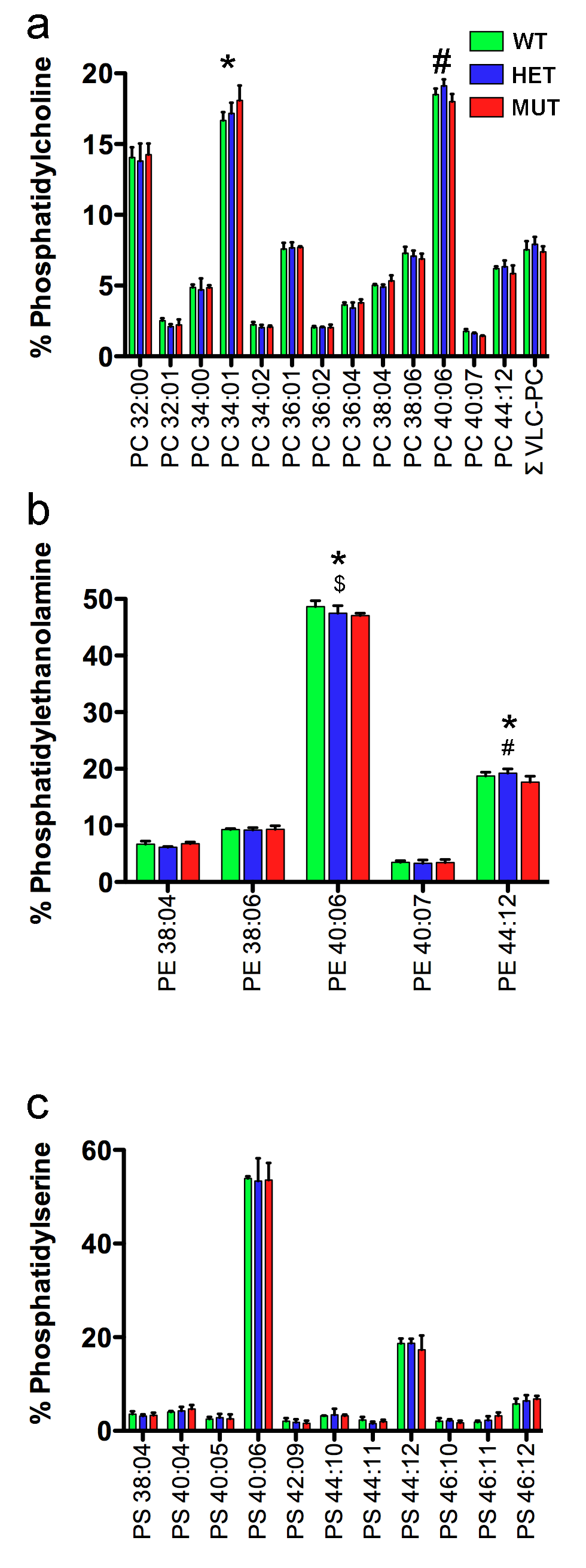

Supplement: Supplementary file 12 — High resolution image (TIF 527 kb) [file 12035_2021_2439_MOESM6_ESM.tif]

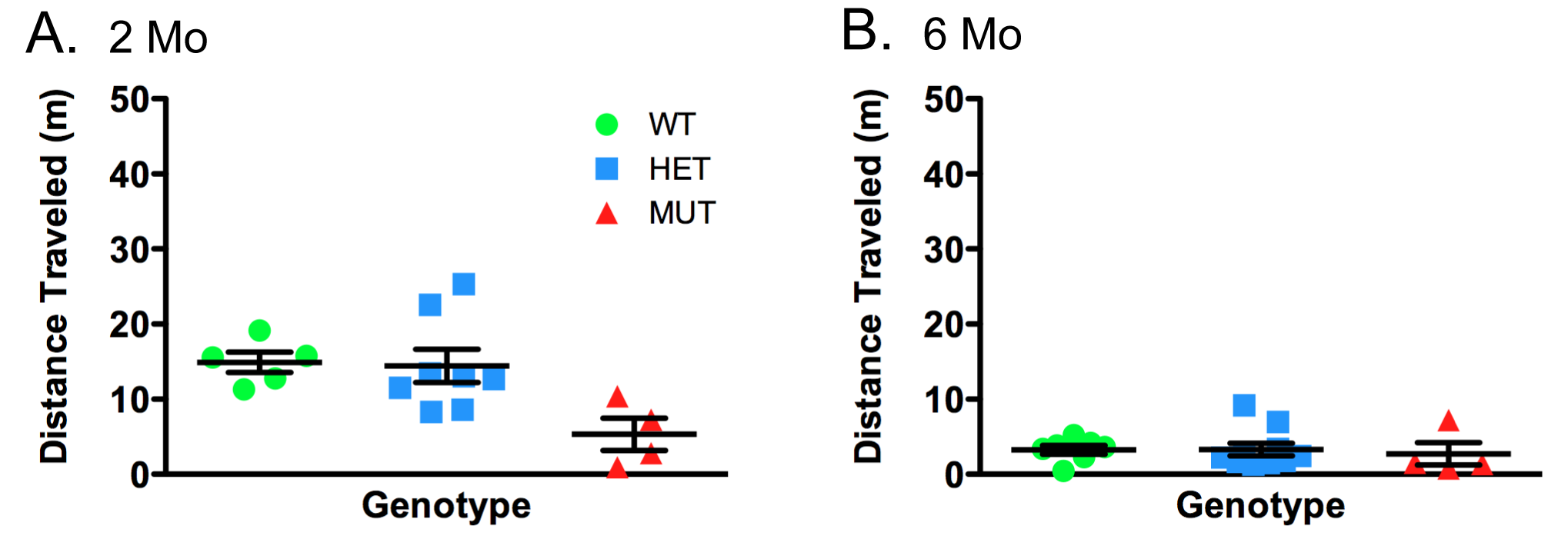

Supplement: Supplementary file 13 — Male WT, HET, and MUT rats all show poor performance on the rotarod test (distance traveled variable shown). (A) Rotarod performance of male WT, HET, and MUT rats at 2 months of age. (B) Rotarod performance of WT, HET, and MUT male rats at 6 months of age. (n= 5 WT, 7 HET, 4 MUT). Analysis by 1-way ANOVA with Tukey’s post-hoc test showed no significant difference across genotypes. (PNG 114 kb) [file 12035_2021_2439_Fig16_ESM.png]

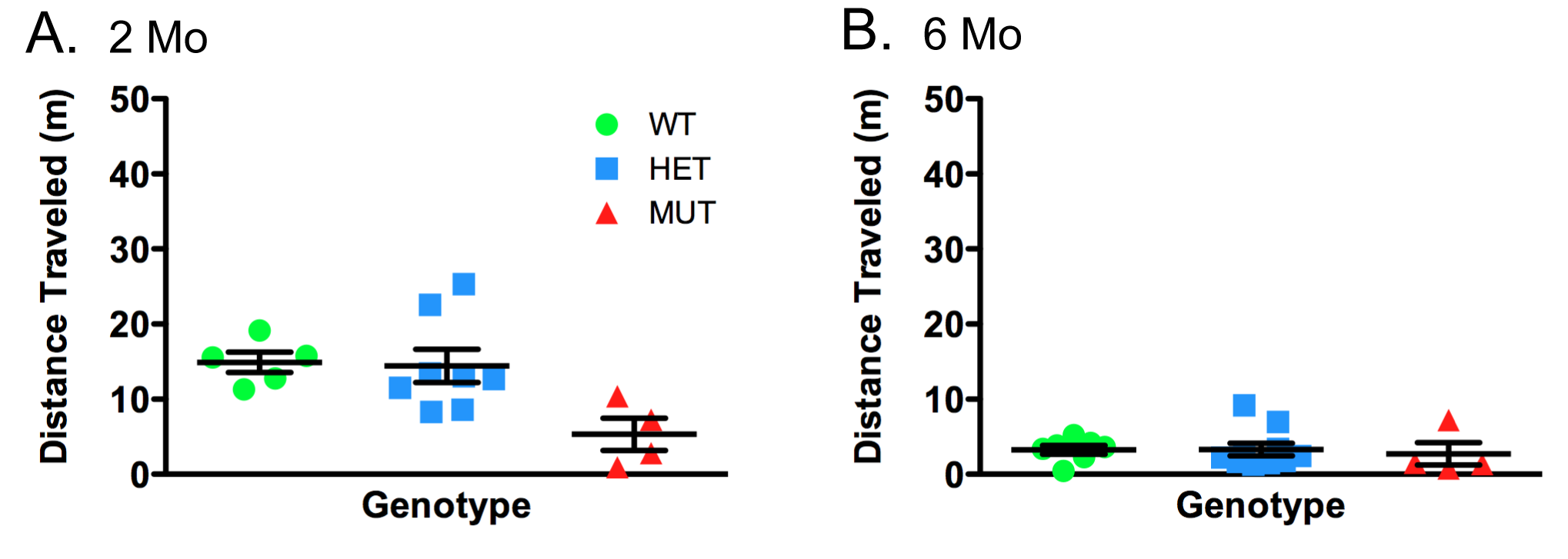

Supplement: Supplementary file 14 — High resolution image (TIF 247 kb) [file 12035_2021_2439_MOESM7_ESM.tif]

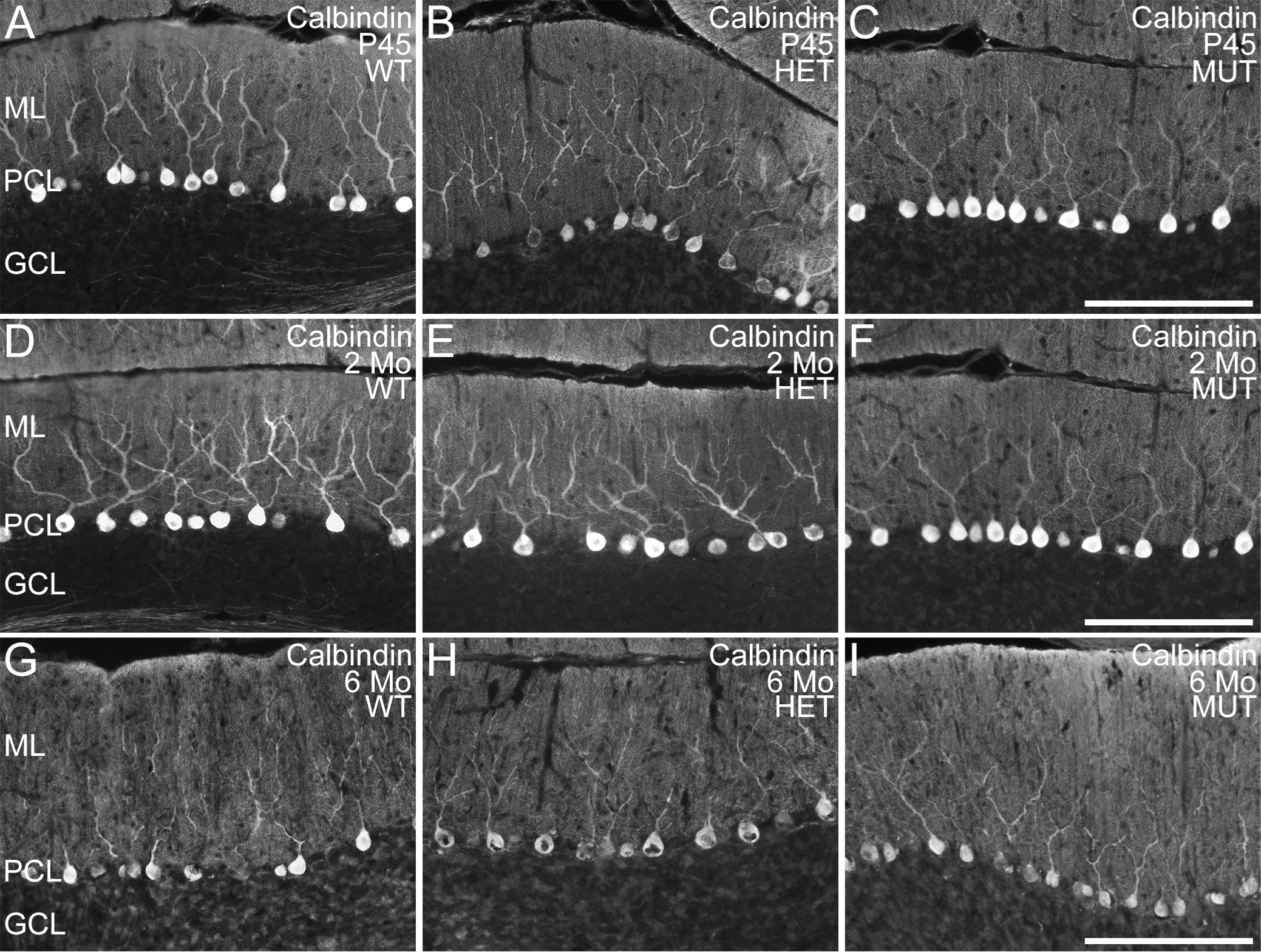

Supplement: Supplementary file 15 — Purkinje cell organization in WT, HET, and MUT rat cerebellum is comparable from P45 to 6 months. Immunolabeling for calbindin shows that Purkinje cells form a monolayer in the Purkinje cell layer (PCL), as appropriate, and have comparable organization across age-matched WT, HET, and MUT rats. (A-C) Postnatal day 45 (P45). (D-F) 2 months of age. (G-I) 6 months of age. Panels G-I of this figure match panels A-C shown in Figure 5. ML, molecular layer; GCL, granule cell layer. Scale bars = 200 μm for each row. (PNG 1872 kb) [file 12035_2021_2439_Fig17_ESM.png]

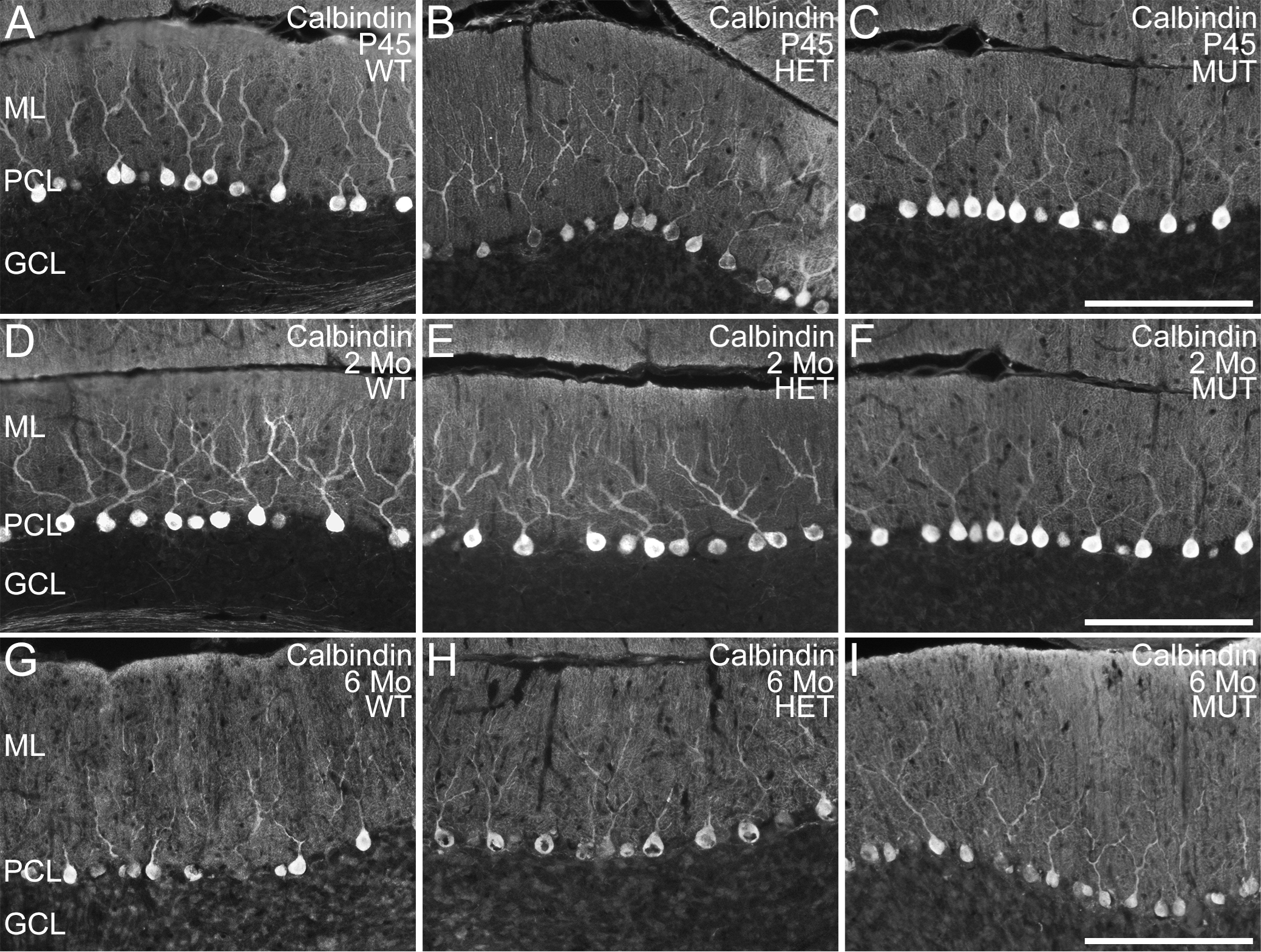

Supplement: Supplementary file 16 — High resolution image (TIF 2787 kb) [file 12035_2021_2439_MOESM8_ESM.tif]

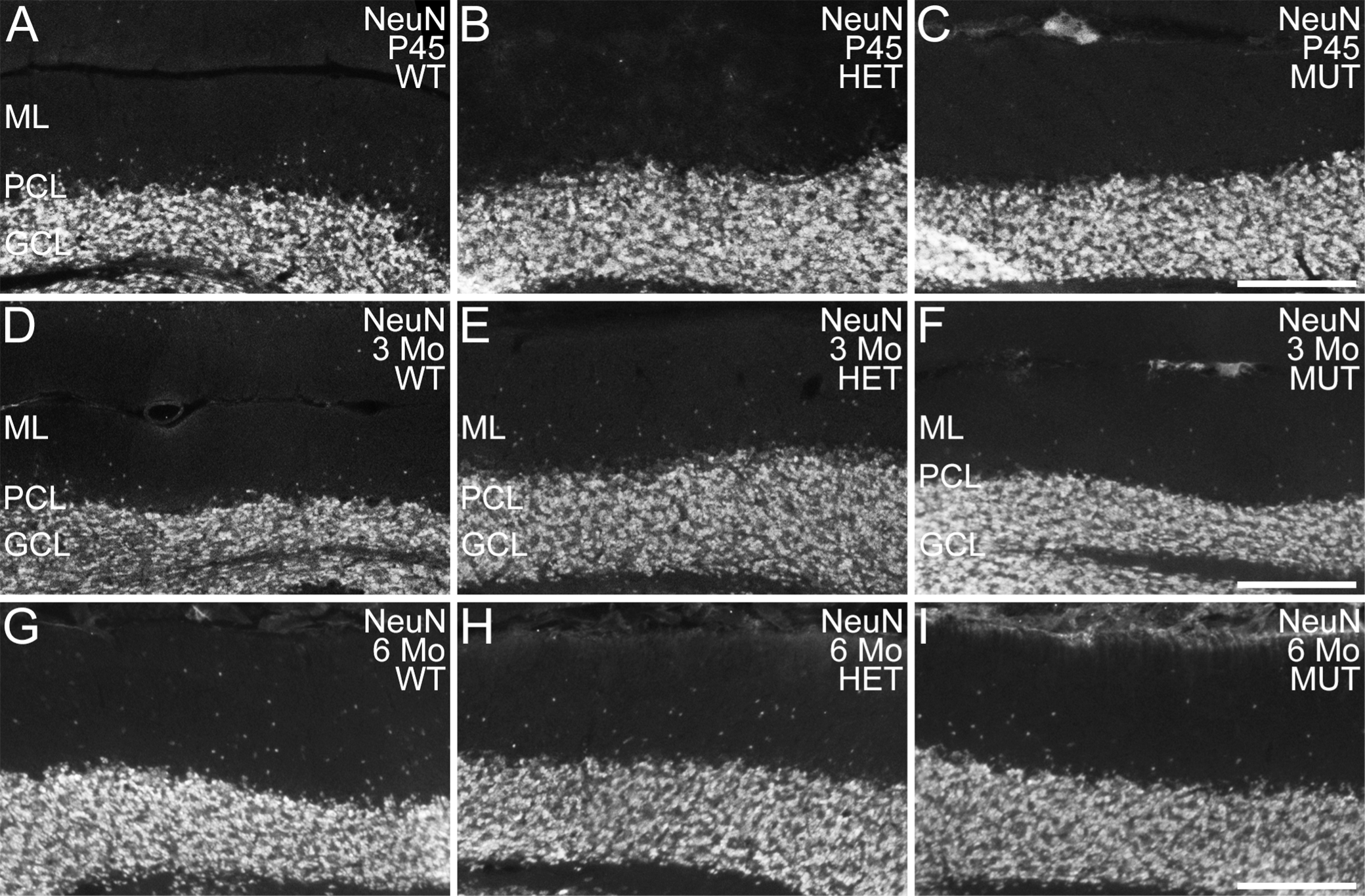

Supplement: Supplementary file 17 — Granule cells in WT, HET, and MUT rat cerebellum are comparable from P45 to 6 months. Immunolabeling for NeuN, a granule cell marker, shows that granule cells are distributed appropriately in the granule cell layer (GCL) in WT, HET, and MUT rat cerebellum from P45 to 6 months of age. A small population of NeuN-positive cells is also present in the molecular layer (ML), as appropriate. (A-B) P45. (D-F) 2 months. (G-I) 6 months. Panels G-I of this figure match panels G-I shown in Figure 5. PCL, Purkinje cell layer. Scale bars = 200 μm for each row. (PNG 1441 kb) [file 12035_2021_2439_Fig18_ESM.png]

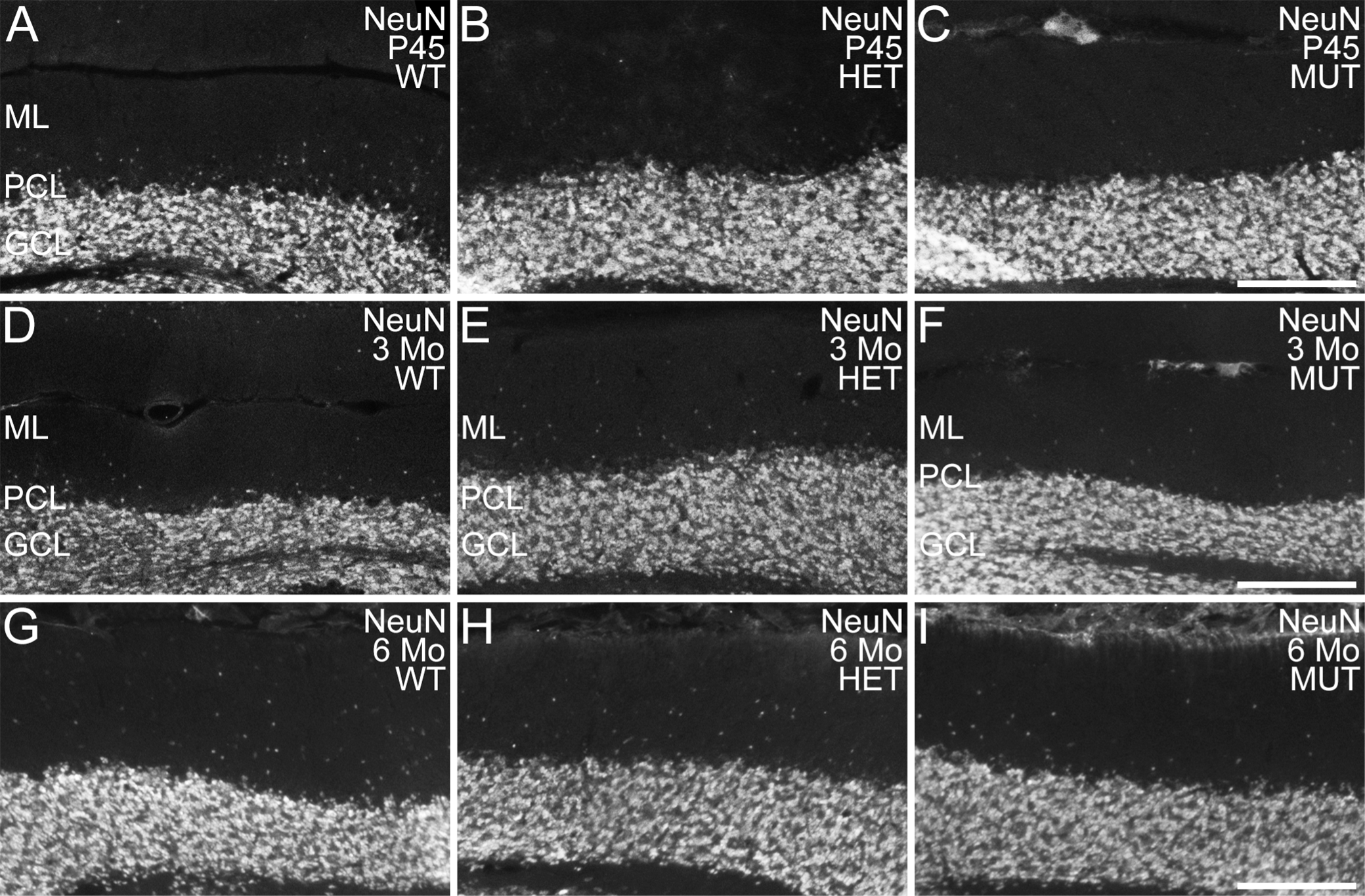

Supplement: Supplementary file 18 — High resolution image (TIF 1941 kb) [file 12035_2021_2439_MOESM9_ESM.tif]

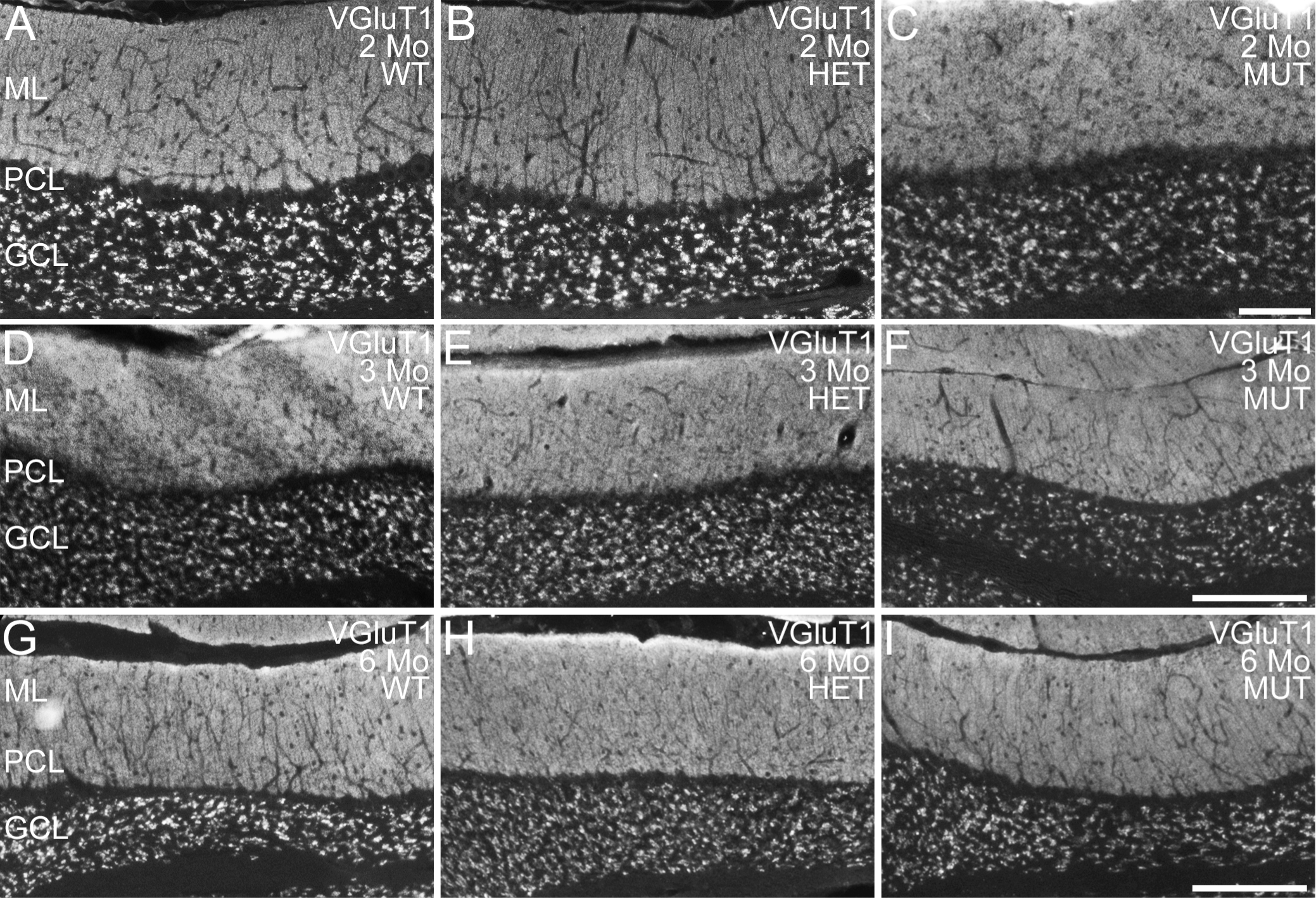

Supplement: Supplementary file 19 — Immunolabeling for Vesicular Glutamate Transporter 1 (VGluT1) in WT, HET, and MUT rat cerebellum is comparable from P45 to 6 months of age. Immunolabeling for VGluT1 shows that mossy fiber inputs project to the glomeruli of the granule cell layer (GCL) and that PFs are distributed throughout the entire molecular layer (ML) of WT, HET, and MUT cerebellum from P45 to 6 months of age, as appropriate. (A-C) P45. (D-F) 2 months. (G-I) 6 months. Panels G-I of this figure match panels J-L shown in Figure 5. PCL, Purkinje cell layer. Scale bars = 200 μm for each row. (PNG 1781 kb) [file 12035_2021_2439_Fig19_ESM.png]

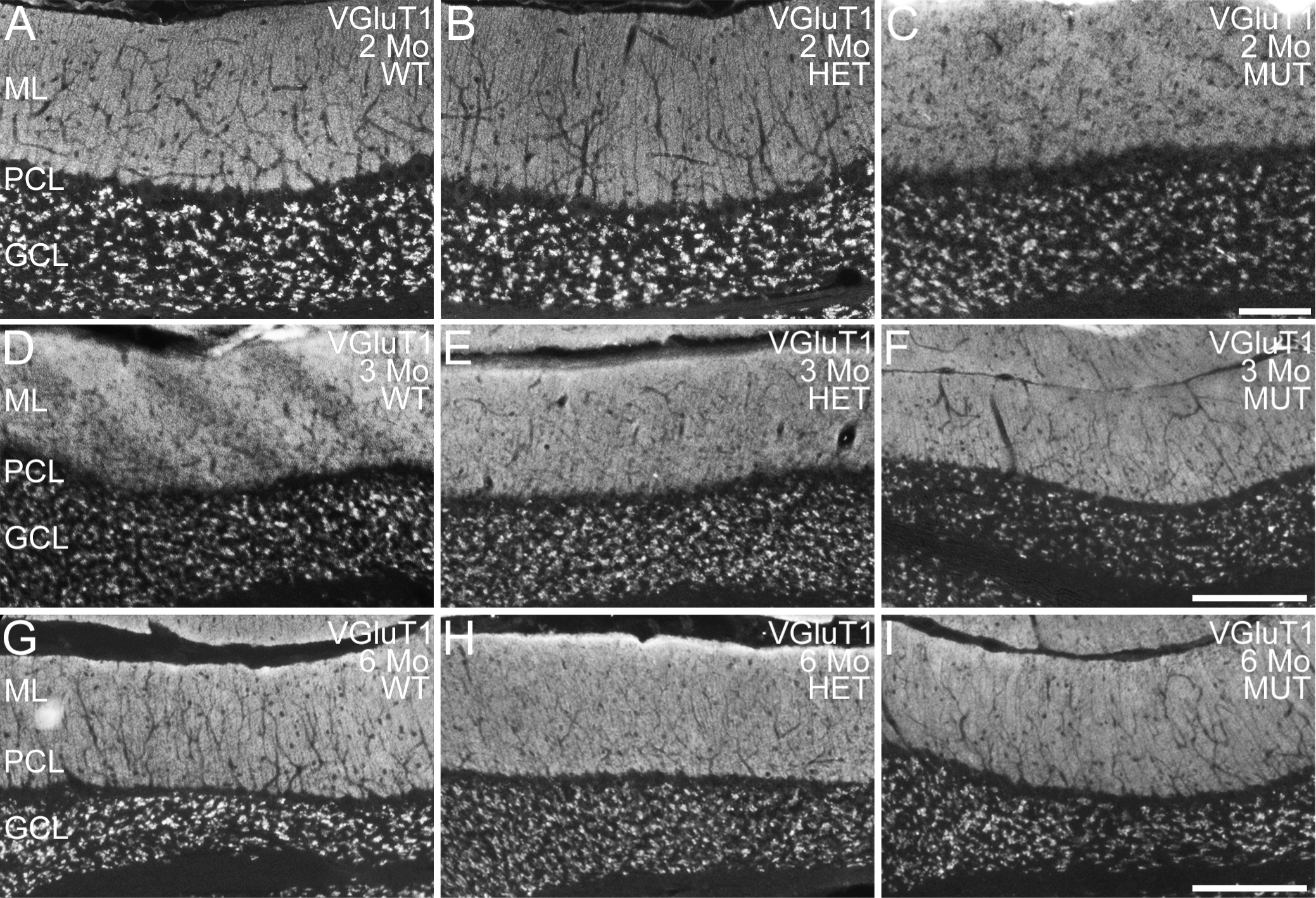

Supplement: Supplementary file 20 — High resolution image (TIF 2514 kb) [file 12035_2021_2439_MOESM10_ESM.tif]

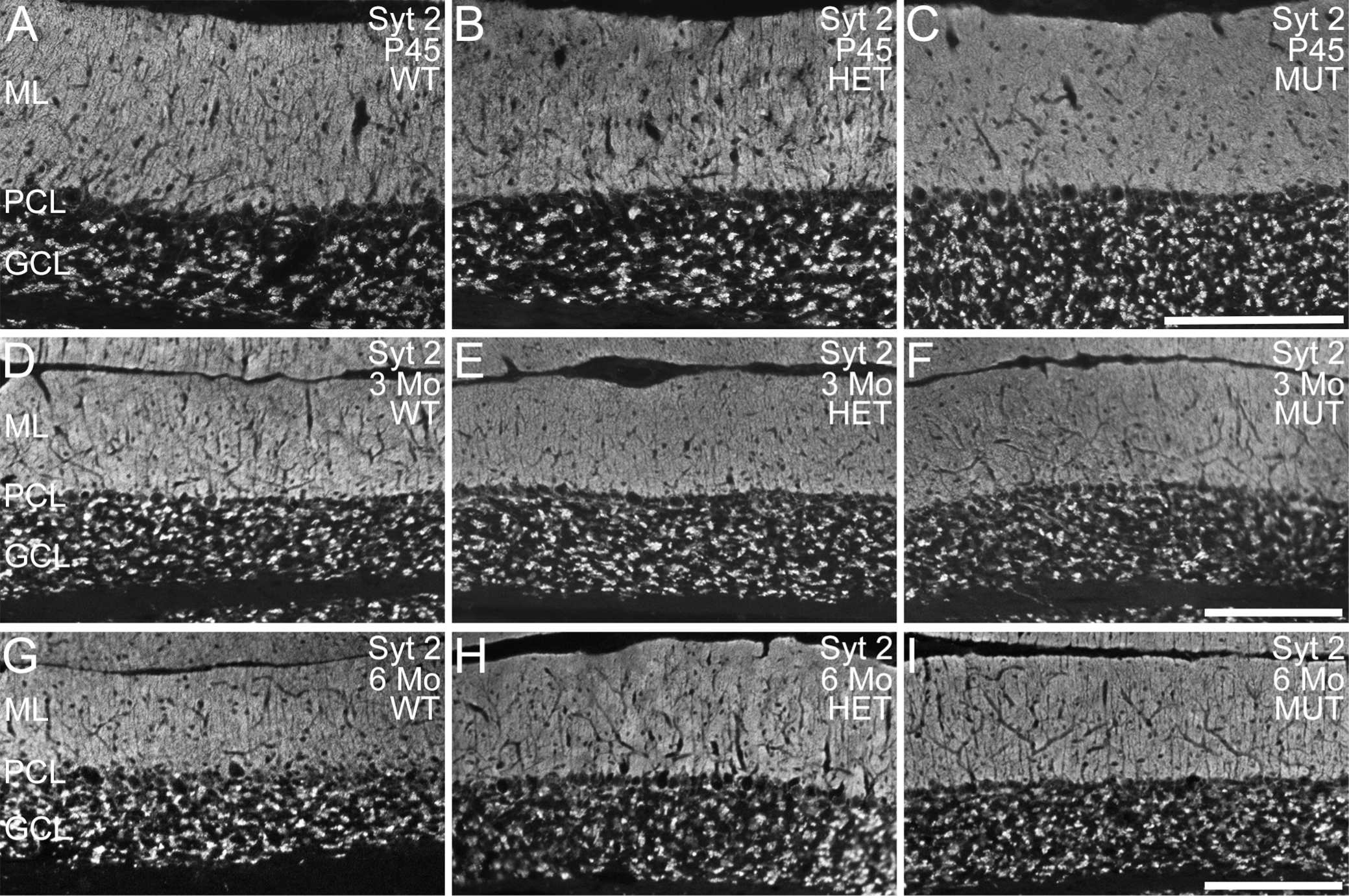

Supplement: Supplementary file 21 — Immunolabeling for synaptotagmin 2 (Syt2) in WT, HET, and MUT rat cerebellum is comparable from P45 to 6 months of age. Syt2 is present in the glomeruli of the granule cell layer (GCL) and throughout the molecular layer (ML), as appropriate. (A-C) P45. (D-F) 2 months. (G-I) 6 months. Panels G-I of this figure match panels M-O shown in Figure 5. PCL, Purkinje cell layer. Scale bars = 200 μm for each row. (PNG 2581 kb) [file 12035_2021_2439_Fig20_ESM.png]

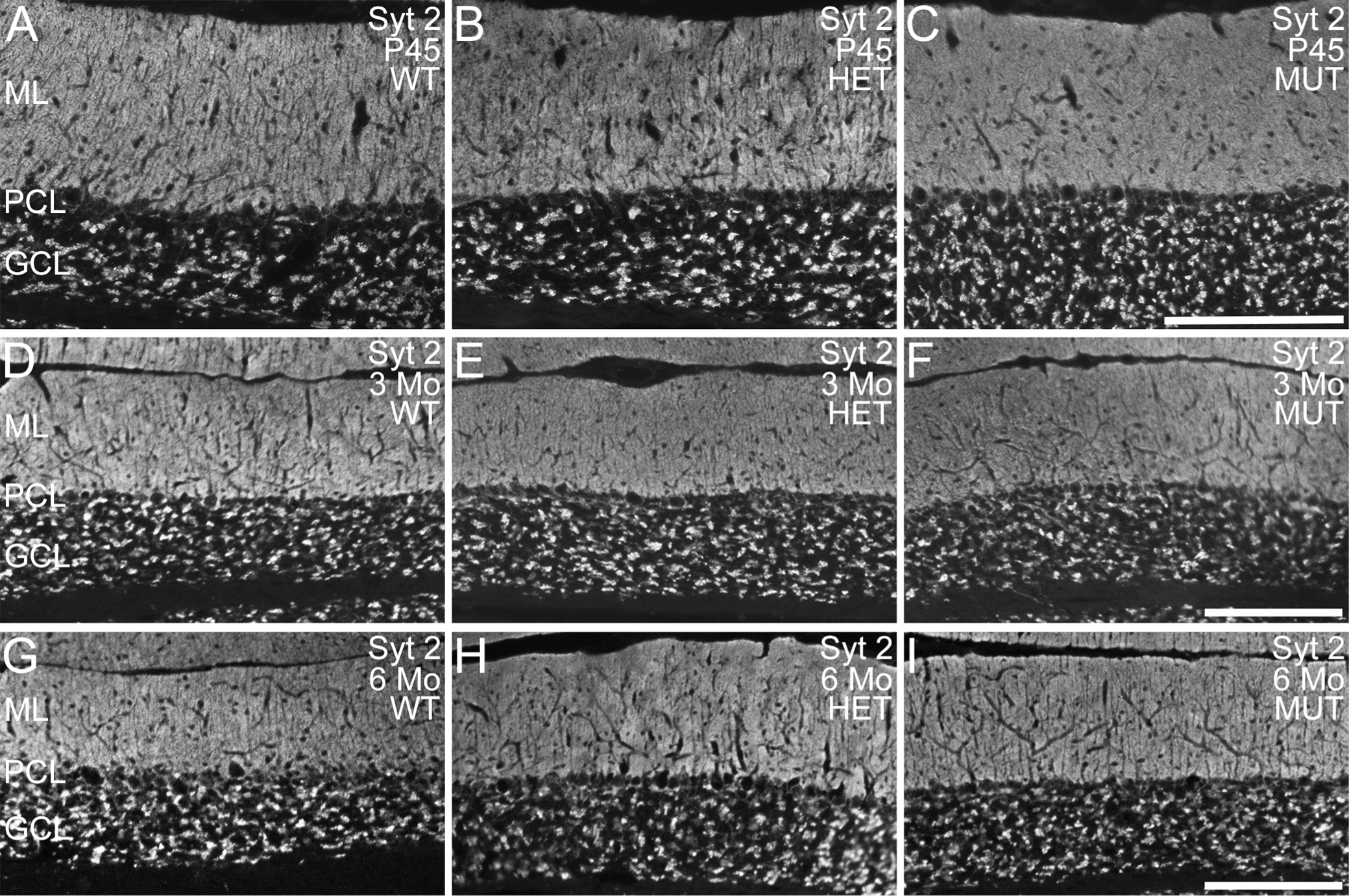

Supplement: Supplementary file 22 — High resolution image (TIF 3672 kb) [file 12035_2021_2439_MOESM11_ESM.tif]

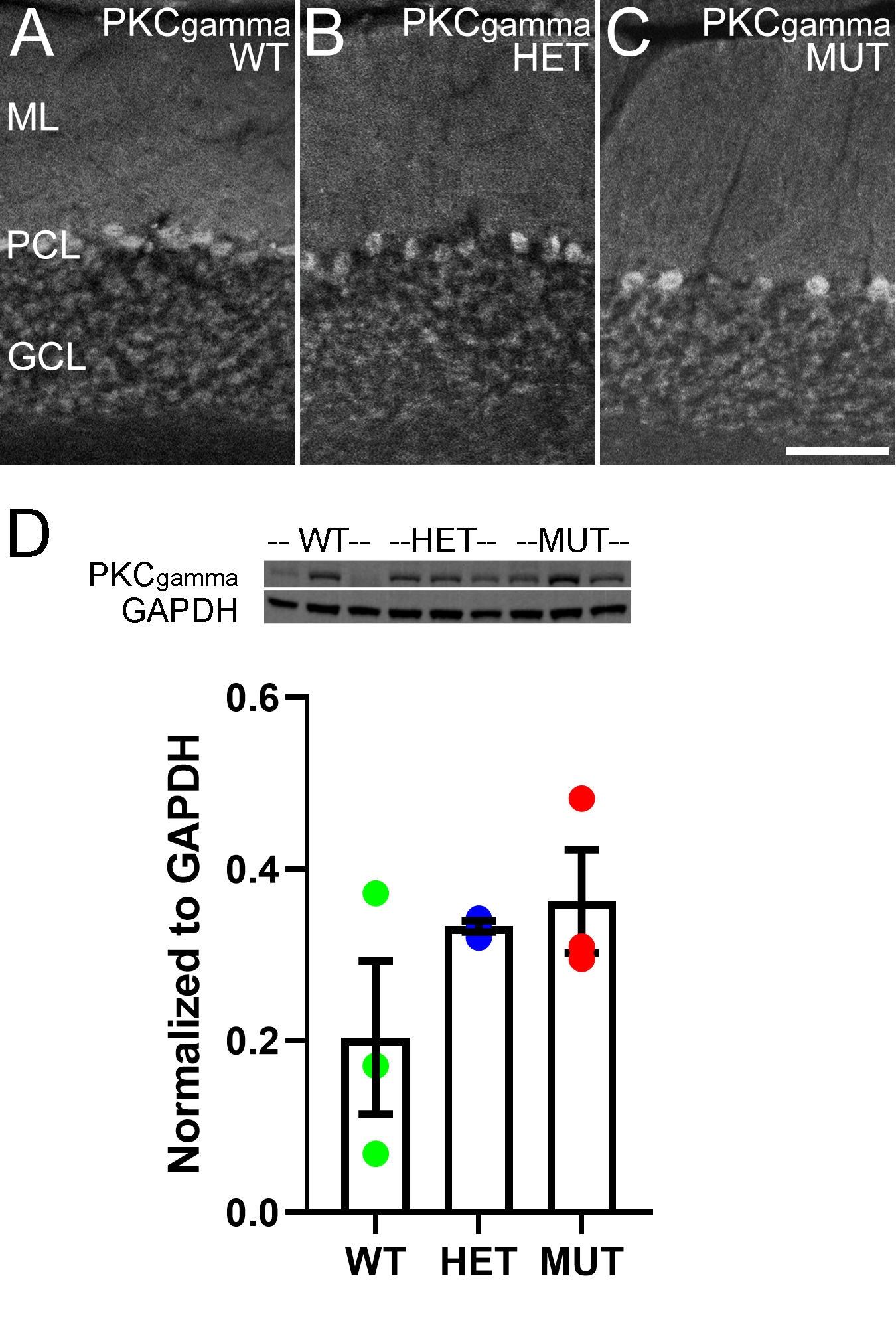

Supplement: Supplementary file 23 — (A-C) Distribution of Protein Kinase Cɣ (PKCɣ) immunolabeling is comparable in WT (A), HET (B), and MUT (C) cerebellum. Scale bar = 100 μm for all panels. (D) Western blotting revealed no significant difference in PKG levels in WT, HET, and MUT cerebellum. (PNG 997 kb) [file 12035_2021_2439_Fig21_ESM.png]

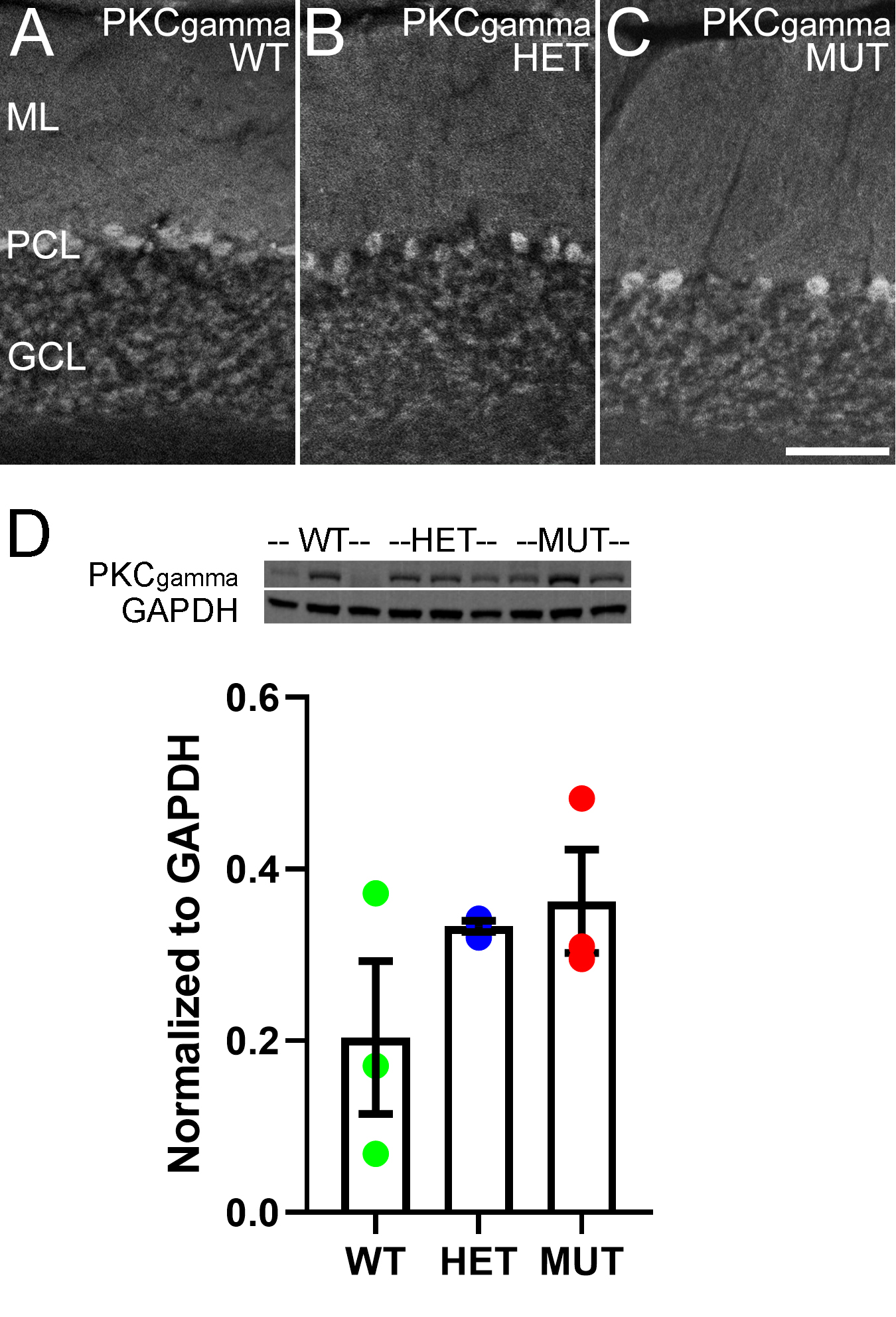

Supplement: Supplementary file 24 — High resolution image (JPG 925 kb) [file 12035_2021_2439_MOESM12_ESM.jpg]

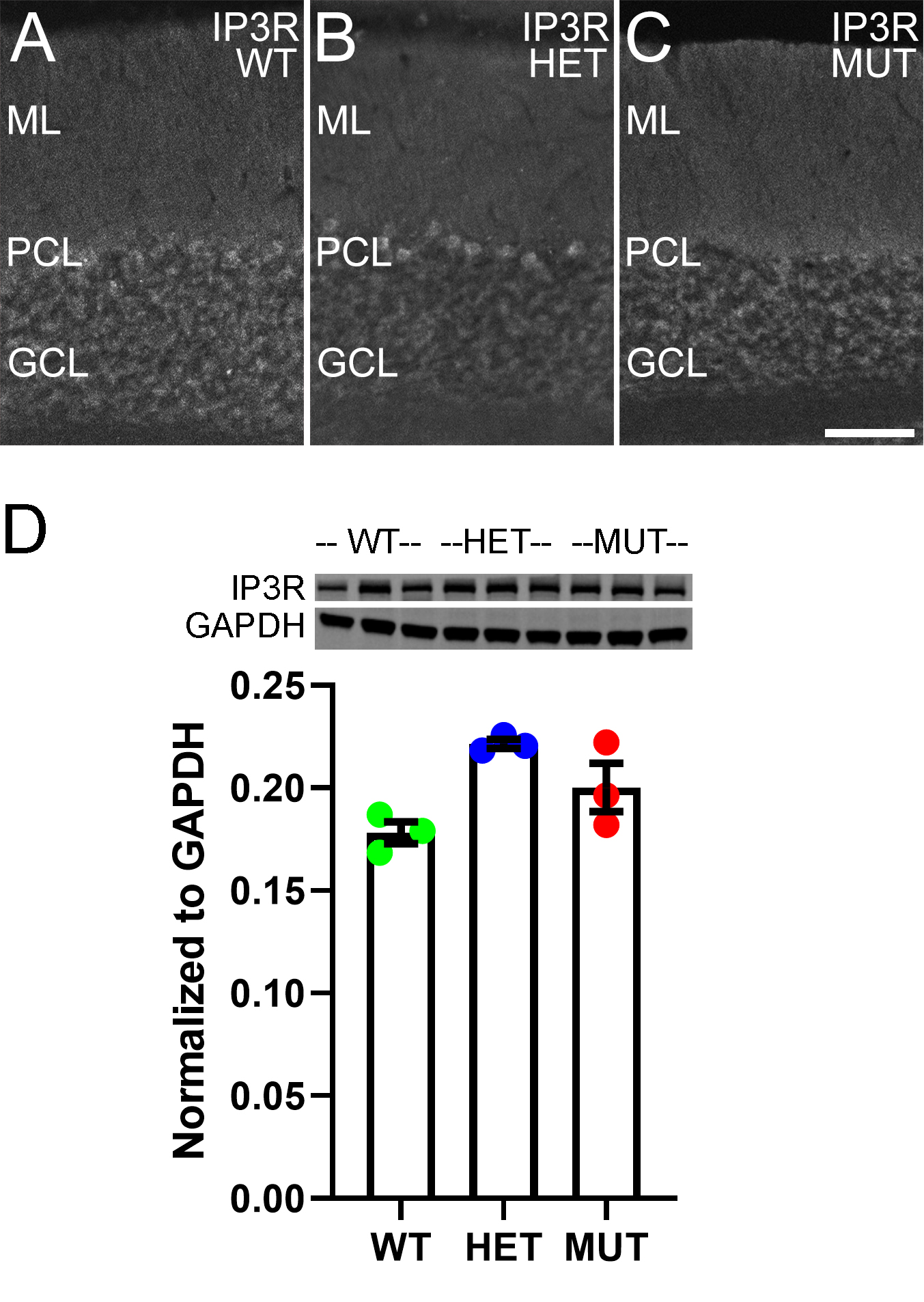

Supplement: Supplementary file 25 — (A-C) Distribution of Inositol Phosphate 3 Receptor (IP3R) immunolabeling is comparable in WT (A), HET (B), and MUT (C) cerebellum. Scale bar = 100 μm for all panels. (D) Western blotting revealed no significant difference in IP3R levels in WT, HET, and MUT cerebellum. (PNG 933 kb) [file 12035_2021_2439_Fig22_ESM.png]

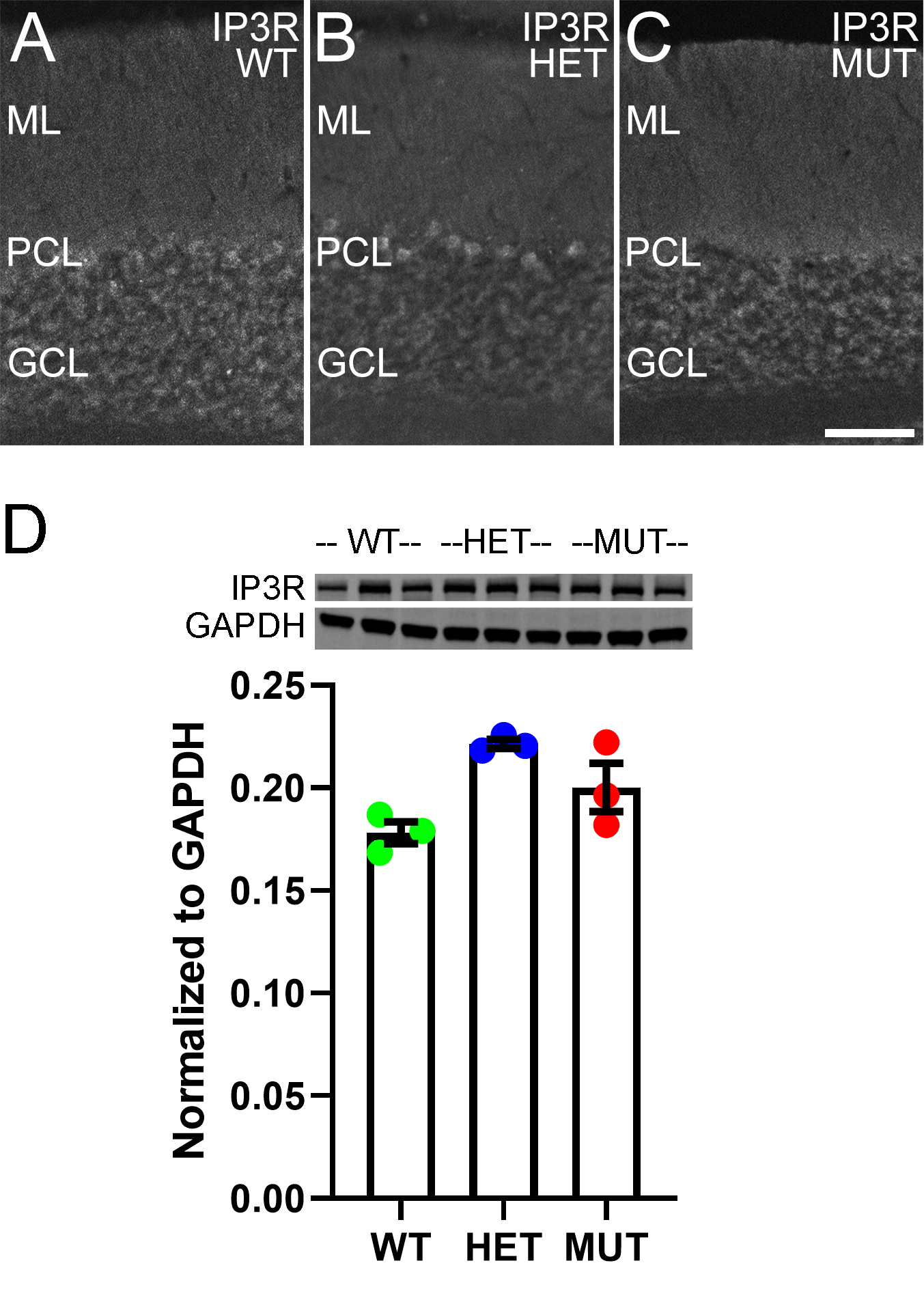

Supplement: Supplementary file 26 — High resolution image (JPG 845 kb) [file 12035_2021_2439_MOESM13_ESM.jpg]

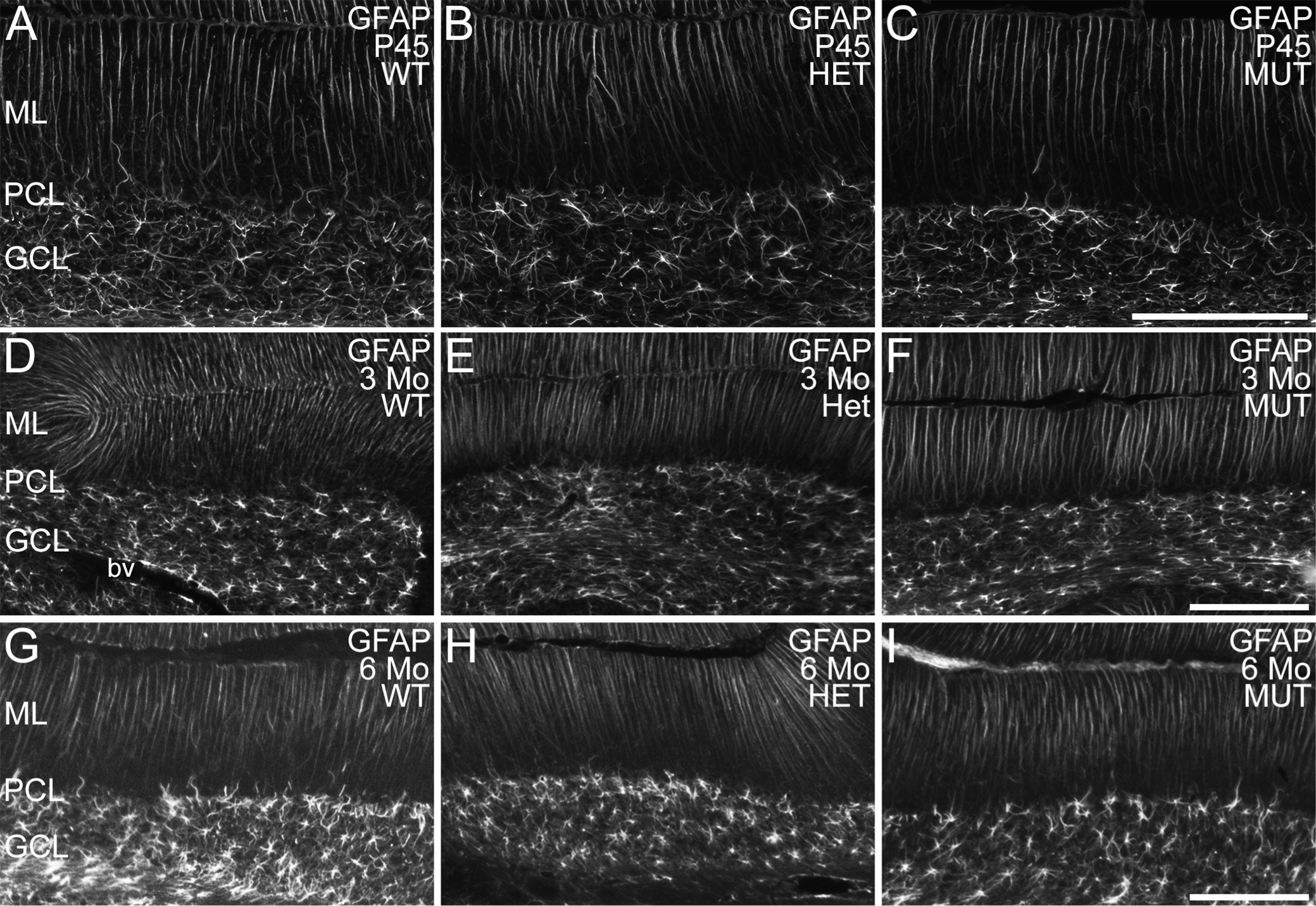

Supplement: Supplementary file 27 — Immunolabeling for Glial Fibrillary Acidic Protein (GFAP) in WT, HET, and MUT rat cerebellum is comparable from P45 to 6 months of age. Immunolabeling for GFAP shows that astrocytes are distributed appropriately, with no evidence of gliosis, in the WT, HET, and MUT rat cerebellum. (A-C) P45. (D-F) 2 months. (G-I) 6 months. ML, molecular layer; PCL, Purkinje cell layer; GCL, granule cell layer; bv, blood vessel. Scale bars = 200 μm for each row. (PNG 1572 kb) [file 12035_2021_2439_Fig23_ESM.png]

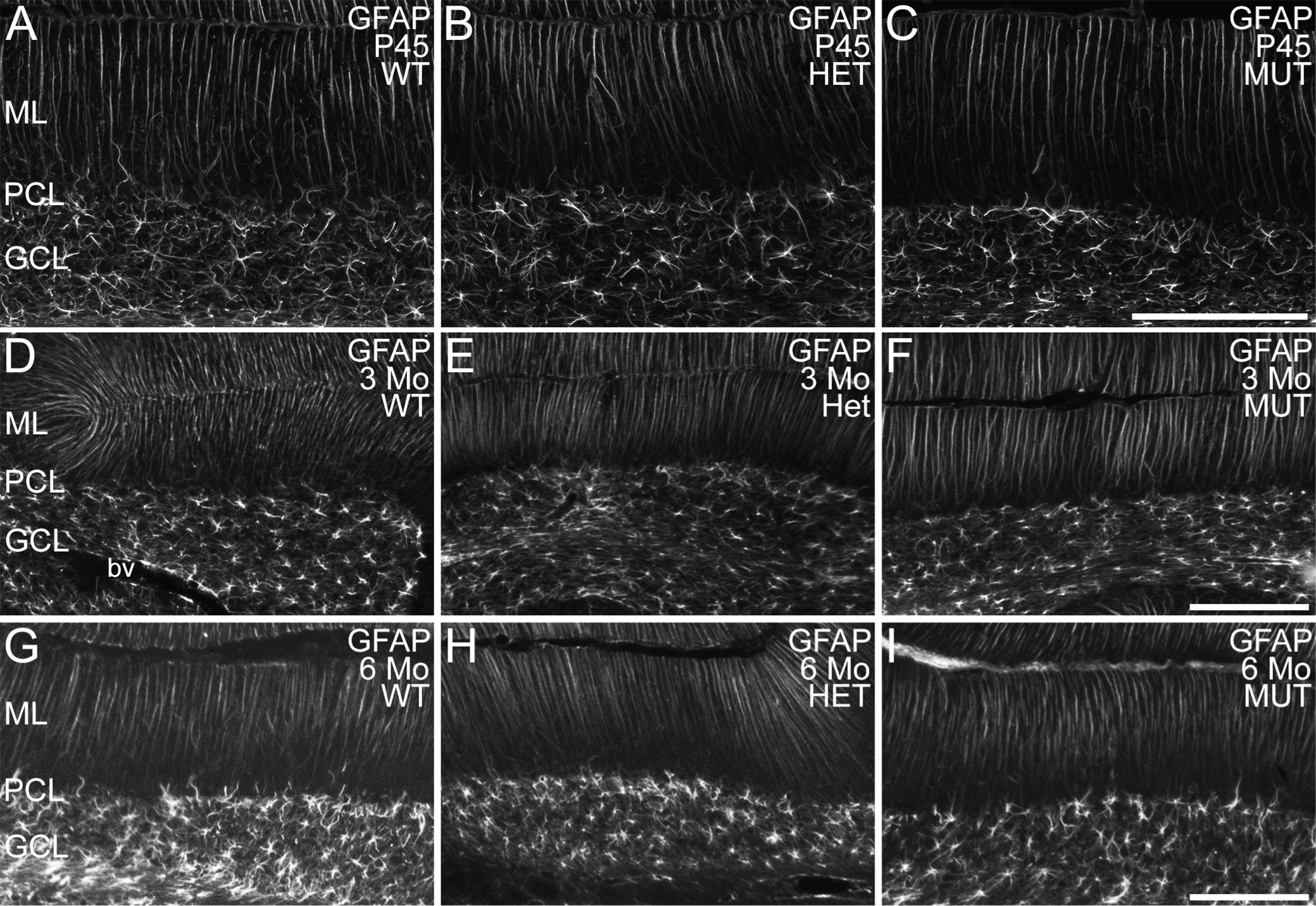

Supplement: Supplementary file 28 — High resolution image (TIF 2274 kb) [file 12035_2021_2439_MOESM14_ESM.tif]

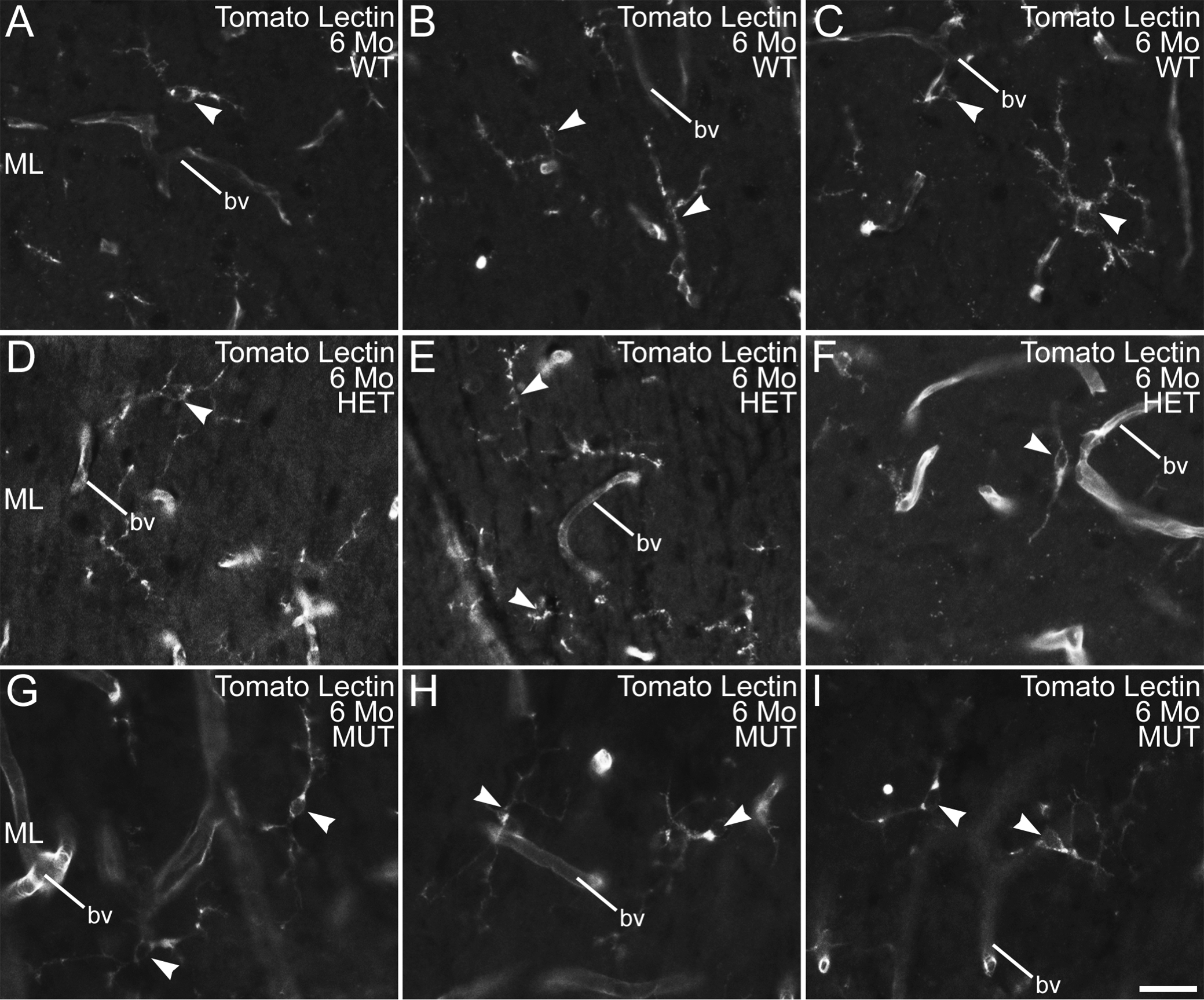

Supplement: Supplementary file 29 — Microglia show “quiescent” ramified morphology in WT, HET, and MUT rat cerebellum. Tomato lectin (TL) labels blood vessels (bv) and microglial cells (arrowheads). Microglia show ramified morphology with symmetrically distributed processes typical of quiescent glia performing surveillance functions in the cerebellum of WT, HET, and MUT rats. (A-C) WT. (D-F) HET. (G-I) MUT. Molecular layer (ML). All images shown at 6 months of age. Scale bar = 200 μm for all panels. (PNG 1519 kb) [file 12035_2021_2439_Fig24_ESM.png]

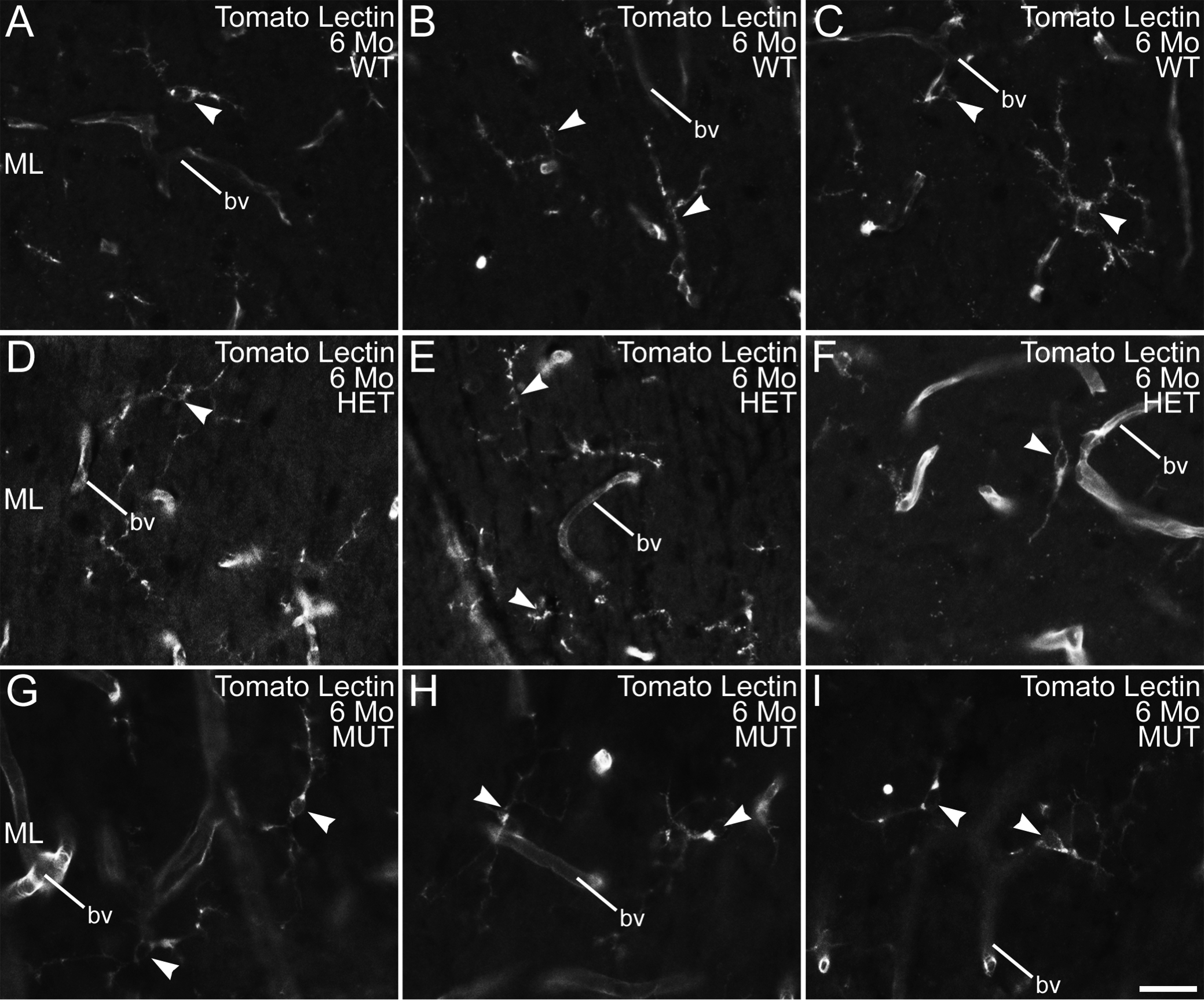

Supplement: Supplementary file 30 — High resolution image (TIF 1690 kb) [file 12035_2021_2439_MOESM15_ESM.tif]

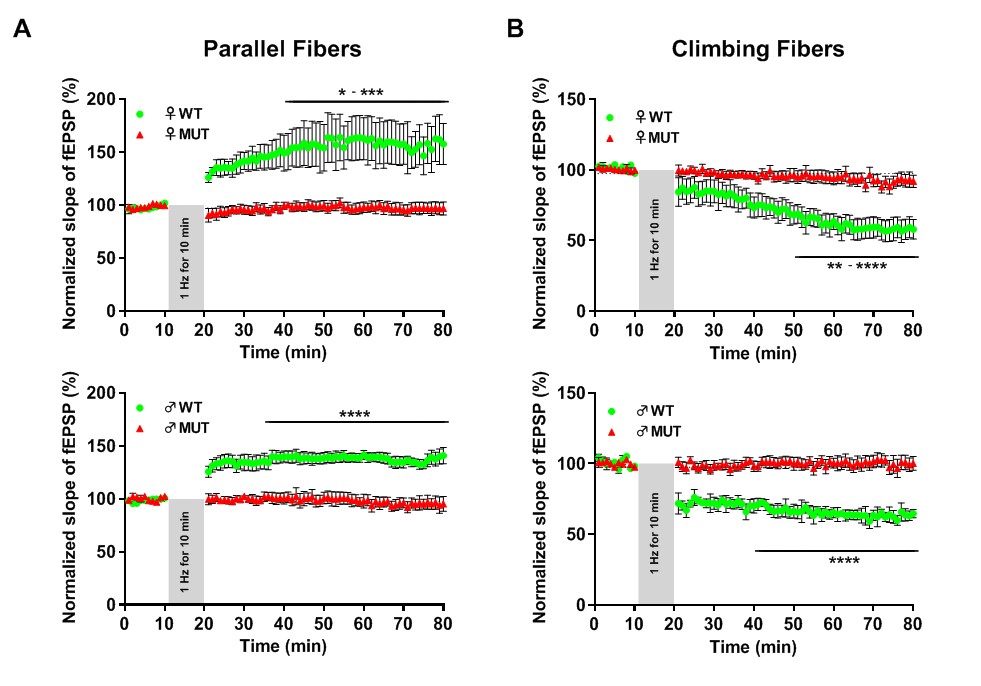

Supplement: Supplementary file 31 — Impaired synaptic plasticity in both male and female mutant rats. (A) LTP induced by stimulation of the PFs in the molecular layer at 1 Hz for 10 min induces LTP in female WT rat cerebellar slices (upper panel, green circles, ♀WT = 155.17 ± 17.38% normalized slope, n = 6 slices from 3 rats), but not in cerebellar slices from female MUT rats (upper panel, red triangles, ♀MUT = 97.87 ± 7.42% normalized slope, n = 8 slices from 3 rats). Similarly, the stimulation of the PFs at 1 Hz induces LTP in male WT rat cerebellar slices (lower panel, green circles, ♂WT = 140.45 ± 8.18% normalized slope, n = 8 slices from 3 rats), but not in cerebellar slices from male MUT rats (lower panel, red triangles, ♂MUT = 93.12 ± 5.94% normalized slope, n = 8 slices from 3 rats). (B) Stimulating the CFs in the granule cell layer at 1 Hz for 10 min induces LTD in female WT cerebellar slices (upper panel, green circles, ♀WT = 59.72 ± 6.66% normalized slope, n = 6 slices from 3 rats), but not in cerebellar slices from female MUT rats (upper panel, red triangles, ♀MUT = 92.13 ± 4.54% normalized slope, n = 8 slices from 3 rats). Similarly, the stimulation of the CFs at 1 Hz induces LTD in male WT rat cerebellar slices (lower panel, green circles, ♂WT = 63.13 ± 4.85% normalized slope, n = 4 slices from 2 rats), but not in cerebellar slices from male MUT rats (lower panel, red triangles, ♂MUT = 92.12 ± 8.14% normalized slope, n = 6 slices from 3 rats). Each data point represents the average of two successive test responses. The vertical gray bar indicates the period of 1 Hz stimulation. Data are shown as mean ± SEM. * p < 0.05, ** p < 0.01, ***p < 0.001, **** p < 0.0001, two-way repeated measures ANOVA with Bonferroni post-hoc test. (PNG 110 kb) [file 12035_2021_2439_Fig25_ESM.png]

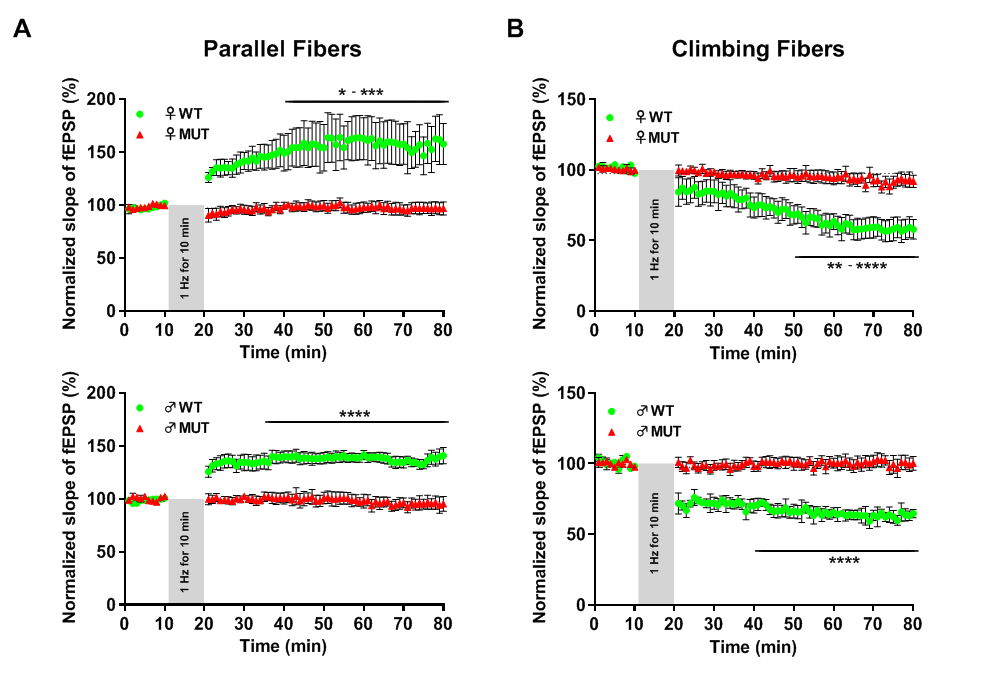

Supplement: Supplementary file 32 — High resolution image (TIF 174 kb) [file 12035_2021_2439_MOESM16_ESM.tif]

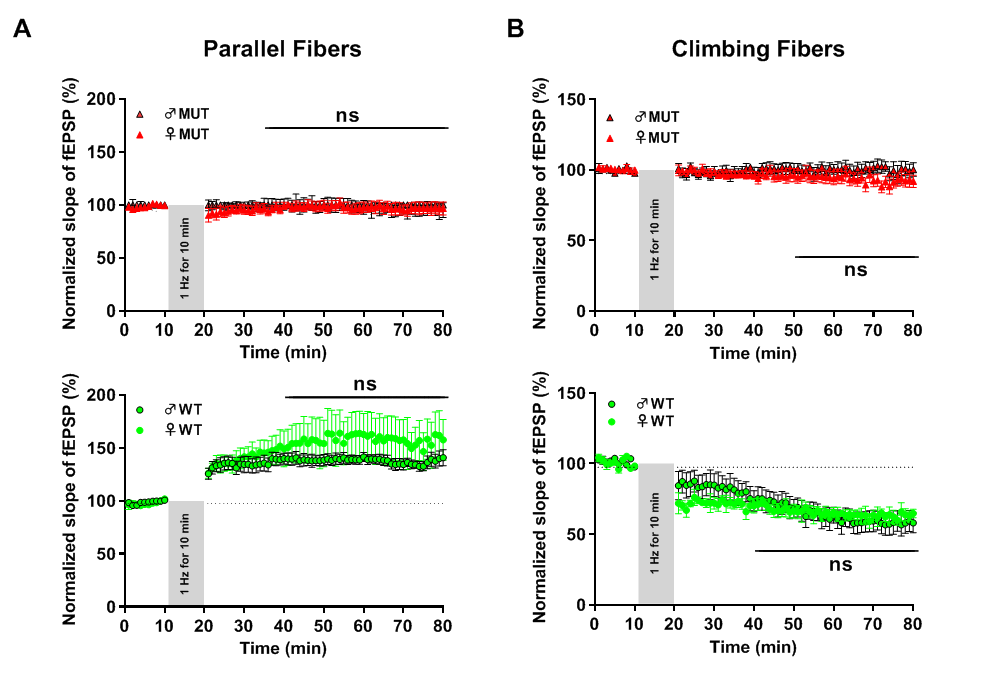

Supplement: Supplementary file 33 — Synaptic plasticity responses do not differ in female and male rats. (A) LTP induced by stimulation of the PFs in the molecular layer at 1 Hz for 10 min does not induce LTP in female MUT rats (upper panel, red triangles, ♀MUT = 97.87 ± 7.42% normalized slope, n = 8 slices from 3 rats) nor in cerebellar slices from male MUT rats (upper panel, red and black triangles, ♂MUT = 93.12 ± 5.94% normalized slope, n = 8 slices from 3 rats). The stimulation of the PFs at 1 Hz induces LTP in female WT rat cerebellar slices (lower panel, green circles, ♀WT = 155.17 ± 17.38% normalized slope, n = 6 slices from 3 rats), and also in male WT rat cerebellar slices (lower panel, green and black circles, ♂WT = 140.45 ± 8.18% normalized slope, n = 8 slices from 3 rats). (B) Stimulating the CFs in the granule cell layer at 1 Hz for 10 min does not induce LTD in cerebellar slices from female MUT rats (upper panel, red triangles, ♀MUT = 92.13 ± 4.54% normalized slope, n = 8 slices from 3 rats) nor in cerebellar slices from male MUT rats (upper panel, red and black triangles, ♂MUT = 92.12 ± 8.14% normalized slope, n = 6 slices from 3 rats). The stimulation of the CFs at 1 Hz induces LTD in female WT cerebellar slices (lower panel, green circles, ♀WT = 59.72 ± 6.66% normalized slope, n = 6 slices from 3 rats), and also in male WT rat cerebellar slices (lower panel, green and black circles, ♂WT = 63.13 ± 4.85% normalized slope, n = 4 slices from 2 rats). Each data point represents the average of two successive test responses. The vertical gray bar indicates the period of 1 Hz stimulation. Data are shown as mean ± SEM. p>0.05 for all panels, two-way repeated measures ANOVA with Bonferroni post-hoc test. (PNG 99 kb) [file 12035_2021_2439_Fig26_ESM.png]

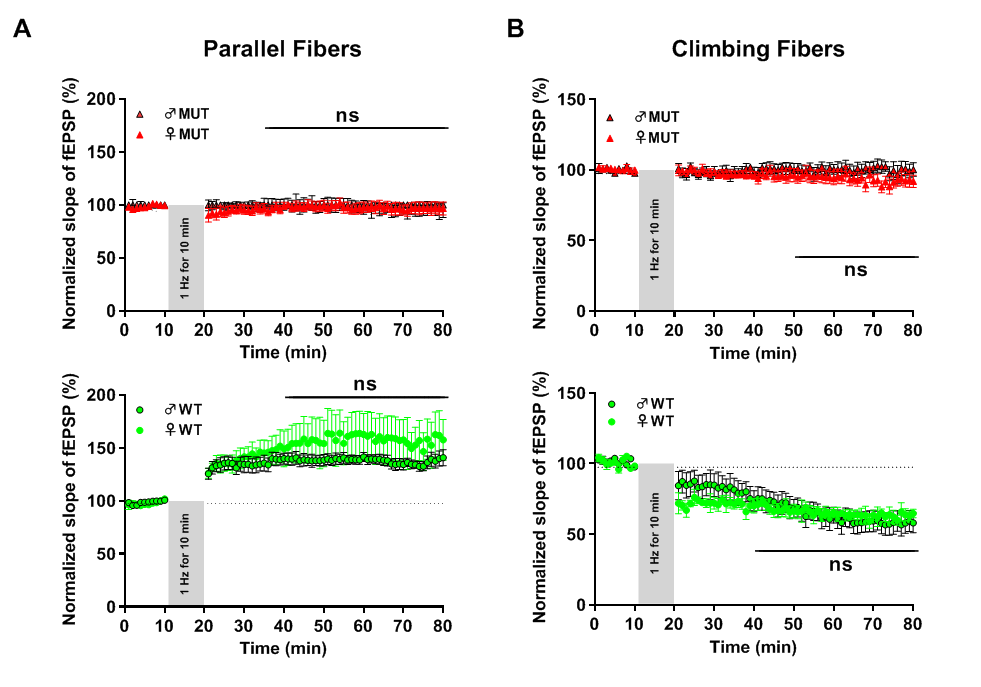

Supplement: Supplementary file 34 — High resolution image (TIF 162 kb) [file 12035_2021_2439_MOESM17_ESM.tif]
